# A transmembrane protein confers the *Yr26*/*Yr24*/*YrCH42*/*YrG22*-mediated stripe rust resistance in wheat

**DOI:** 10.1038/s41467-026-76091-5

**Published:** 2026-08-24

**Authors:** Shengjie Liu, Keqing Mu, Shuqing Yang, Jianhui Wu, Mingjie Xiang, Xiangrui Cheng, Xiaoting Wang, Yibo Zhang, Ying Zhao, Jiangna Han, Shuo Huang, Haitao Dong, Jiwen Zhao, Fengping Yuan, Raghvendra Sharma, Michael Norman, Peidu Chen, Aizhong Cao, Weijun Zheng, Chunlian Li, Jaroslav Doležel, Kateřina HolušováJ, John Rathjen, Evans Lagudah, Xiaojie Wang, Zhensheng Kang, Sambasivam Periyannan, Dejun Han, Qingdong Zeng

**Affiliations:** 1 State Key Laboratory of Crop Stress Resistance and High-Efficiency Production, College of Plant Protection, Northwest A&F University, Yangling, PR China; 2 State Key Laboratory of Crop Stress Resistance and High-Efficiency Production, College of Agronomy, Northwest A&F University, Yangling, PR China; 3 Key Laboratory of Plant Design, Chinese Academy of Sciences, CAS Center for Excellence in Molecular Plant Sciences, Shanghai Institute of Plant Physiology and Ecology, Chinese Academy of Sciences, Shanghai, PR China; 4 Agriculture and Food, Commonwealth Scientific and Industrial Research Organisation, Canberra, ACT Australia; 5 College of Agronomy of Nanjing Agricultural University/State Key Laboratory of Crop Genetics & Germplasm Enhancement and Application /JCIC-MCP, Nanjing, PR China; 6 Institute of Experimental Botany of the Czech Academy of Sciences, Centre of Plant Structural and Functional Genomics, Olomouc, Czechia; 7 Research School of Biology, Australian National University, Canberra, ACT Australia; 8 School of Science, Engineering and Digital Technologies, Centre for Crop Health, University of Southern Queensland, Toowoomba, QLD Australia

**Keywords:** Plant breeding, Plant genetics, Biotic, Molecular evolution

## Abstract

Stripe or yellow rust (YR), caused by *Puccinia striiformis* Westend. f. sp. *tritici* (*Pst*), is a devastating fungal disease of wheat. Here, we report the map-based cloning of *Yr26* (synonyms: *Yr24*, *YrCH42*, and *YrG22*), a major stripe rust resistance gene, and its validation by gene silencing, mutation analysis and transgenic complementation. *Yr26*, which firstly arises in emmer wheat and recently has introgressed into bread wheat, is a newly evolved gene encoding a transmembrane protein. AlphaFold v3.0 predictes a C-terminal four-helical bundle domain that is critical for triggering Ca^2+^-dependent cell death, a hallmark of the hypersensitive response. The cloning of *Yr26* offers not only a genetic resource for pyramiding stripe rust resistance genes in wheat breeding but also an opportunity to study host–pathogen co-evolution.

## Abstract (web summary)

Yellow rust (YR) is a devastating fungal disease of wheat. Here, the authors report the cloning of *Yr26* as a transmembrane protein and reveal its evolves within the *Triticeae* lineage as an orphan gene.

## Introduction

Bread wheat (*Triticum aestivum*) accounts for ~20% of global calorie intake and provides the basis of global food security. Stripe rust, or yellow rust (YR), caused by *Puccinia striiformis* Westend. f. sp. *tritici* (*Pst*), poses a major threat to wheat production in most wheat-growing regions^1^. Deploying diversified resistance genes in cultivars is the most practical, environmental-friendly and economical way to control this devastating disease^2^. Thus, it is important to clone diverse resistance genes and determine the underlying mechanisms of action.

So far, 87 *Yr* genes cataloged^3^ and many additional genes are temporarily designated. In addition, more than 400 quantitative trait loci (QTL) in 83 QTL-rich clusters^4,5^ have been reported in wheat and its wild relatives. However, only 14 *Yr* genes have been cloned due to the size and complexity of the wheat genome^6^. These include three adult plant stage partial resistance genes: *Yr18* (encoding a putative ATP-binding cassette (ABC) transporter)^7^, *Yr36* (encoding a Kinase-START)^8^ and *Yr46* (encoding an altered hexose transporter)^9^. By contrast, all-stage resistance (ASR) genes typically encode nucleotide-binding site (NBS) and leucine-rich repeat (LRR) domain-containing proteins (NLRs). Examples include *Yr6*^4,10,11^, *Yr9*^12–14^, *Yr27*^15,16^, *Yr28*/*YrAS2388*^17^, *Yr84*^18,19^ and *Yr87*^6^. Some NLRs contain integrated domains (NLR-IDs): *Yr5a*, *Yr5b* (*YrSP*) and *Yr7* encode NLR proteins with BED domains^20^, and *YrU1* encodes an NLR protein with an N-terminal ankyrin-repeat and a C-terminal WRKY domain^21^. These additional domains often function as integrated decoys to detect pathogen effectors and activate immune signaling. *Yr15*^22^ encodes a putative kinase-pseudo-kinase protein (wheat tandem kinase 1, WTK1), representing a new class of resistance genes^23^. *Yr10*/*YrNAM* harbors NAM and ZnF-BED domains, both of which are required for resistance^24,25^.

The stripe rust resistance gene, variously named as *Yr24*, *Yr26*, *YrG22* and *YrCH42*, was mapped to the centromeric region of chromosome 1B in durum wheat (genome AABB) and synthetic hexaploid wheat (AABBDD) derived from an amphiploid hybrid of durum wheat and *Aegilops tauschii*^26^. *Yr24* originated from a synthetic wheat line AUS18911 generated by crossing *T. durum* accession K733 with *Ae. tauschii* (DD) and subsequent analysis showed that *Yr24* was derived from K733^27^. *Yr26* was initially identified in translocation line 92R137 (pedigree: Yangmai 5/4/*T. durum* cv. *γ*80-1/*Haynaldia villosa*//Ningmai 6/3/Yangmai 2), a 6AS·6VS translocation line developed to transfer powdery mildew resistance gene *Pm21* from *Dasypyrum villosum* to wheat^28^. Early observations revealed that several lines carrying *Pm21* were resistant to stripe rust, leading to an assumption that *Yr26* was in the same alien chromosome segment^29^. Subsequent genetic analysis located *Yr26* to chromosome arm 1BL and identified the *T. durum* parent *γ*80-1 as the likely donor^30^. Similarly, *YrG22* was identified in the *D. villosum*-derived line Guinong 22, with resistance inherited from its *T. durum* parent cv. Sauwene^31^. *YrCH42* was identified in Chinese wheat cultivar Chuanmai 42, a derivative of one of many CIMMYT synthetic wheat lines introduced to China during the late 1980s and 1990s^26,32^. Seedling tests of lines carrying the *Yr24*, *Yr26*, and *YrCH42* sources using 26 *Pst* isolates generated identical host response arrays indicative of a common resistance gene^32^. This hypothesis was supported by previous fine-mapping by SNP-based bulk segregant analysis and haplotype characterization using flanking Kompetitive allele-specific polymerase chain reaction (KASP) markers *WRS435* and *WRS312*^31^. However, it remained unclear whether these four genes represent the same gene or different alleles.

In this work, we report the cloning of *Yr26* and *Yr24* and confirm they are identical to *YrCH42* and *YrG22*. As most experiments reported here use the *Yr26* donor line 92R137, we hereafter designate this locus as *YR26*. The gene is *Pst* race-specifically inducted, and triggers cell death in wheat protoplasts and *Nicotiana benthamiana* leaves. AlphaFold v3.0 predicts that *Yr26* encodes a transmembrane protein featuring a C-terminal four-helical bundle domain, which is essential for mediating cell death. Evolutionary and phylogenetic analyses indicate that the functional resistance allele *Yr26* is a recently evolved orphan gene in the *Triticeae* and likely first arises in emmer wheat.

## Results

### Stripe rust responses of plants containing *Yr26*

**Fig. 1:**
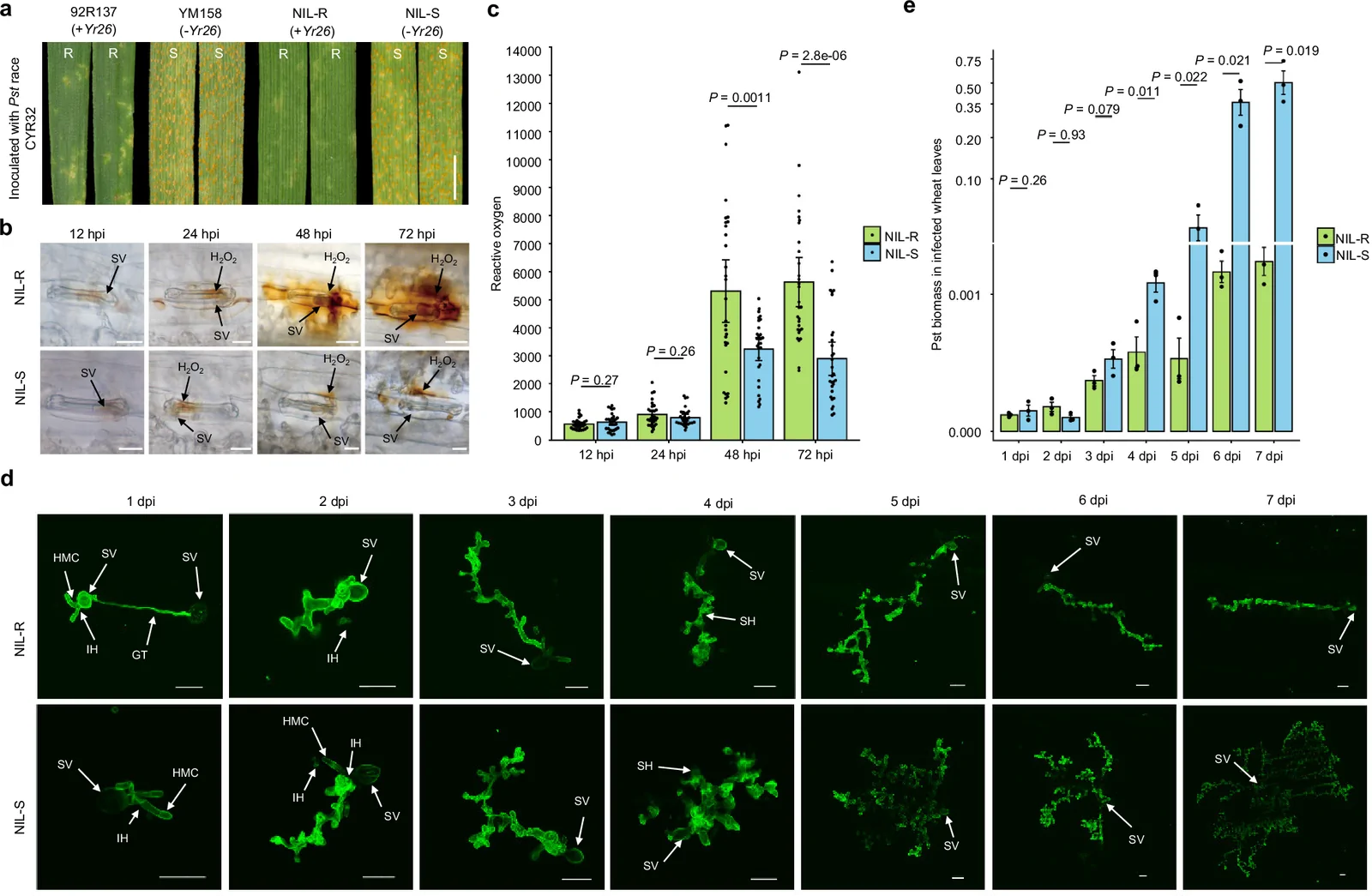
Responses of Yr26 to avirulent *Puccinia striiformis* f. sp. *tritici* (*Pst*) race CYR32. **a** Wheat lines 92R137 (+*Yr26*), YM158 (-*Yr26*), and the near-isogenic line pair NIL-R: +*Yr26*; NIL-S: -*Yr26* infected with avirulent *Pst* race CYR32 at 14 days post inoculation (dpi). R: resistant, S: susceptible. Scale bar, 1 cm. **b**, **c** Histology and statistics of reactive oxygen production in *Yr26* near-isogenic lines inoculated with CYR32 at 12 hpi, 24 hpi, 48 hpi and 72 hpi. SV substomatal vesicle. Data are means ± s.d. (*n* = 30 independent infection sites); each infection site as a single point. Scale bars, 20 μm. **d** Histology and cytology of fungal development during incompatible (resistant) and compatible (susceptible) interactions at 1–7 dpi. S, urediniospore; SV substomatal vesicle, GT germ tube, IH infection hypha, HMC haustorial mother cell, H haustoria. *n* > 30 independent infection sites. Scale bars, 40 μm. **e** Biomass of *Pst* race CYR32 in infected leaves of *Yr26* near-isogenic lines. Data are means ± s.d. (*n* = 3 biological replicates); each replicate as a single point. For all experiments shown in this figure, randomization or blinding was applied; no data were excluded. Statistical significance was determined using two-tailed Student’s *t*-tests (rs*t*atix v0.7.3); *P* > 0.05, n.s. Source data are provided as a Source Data file.

To characterize *Yr26*-mediated resistance, seedlings of lines 92R137 (+*Yr26*), Yangmai 158 (YM158, *-Yr26*), and near-isogenic lines NIL-R (+*Yr26*)/NIL-S (*-Yr26*) were inoculated with *Pst* race CYR32, which is avirulent to *Yr26* (Supplementary Data 1). At 14 days post inoculation (dpi), 92R137 and NIL-R exhibited hypersensitive responses (HR) without visible sporulation, whereas YM158 and NIL-S were susceptible showing abundant uredinia (Fig. 1a). Diaminobenzidine (DAB) staining confirmed strong reactive oxygen species (ROS) accumulation at infection sites in NIL-R compared to NIL-S at 48 and 72 h post inoculation (hpi), but no significant difference at 12 hpi and 24 hpi (Fig. 1b, c).

Microscopy and *Pst* biomass quantification revealed comparable fungal development (substomatal vesicles, primary infection hyphae, haustorial mother cells, and haustoria) between NIL-R and NIL-S during early infection (1–4 dpi) (Fig. 1d, e). However, by 5–7 dpi, NIL-R showed a markedly reduced number of haustorial mother cells, attenuated hyphal growth, and fewer haustoria compared to the extensive colonization observed in NIL-S. These results indicated that *Yr26* inhibited post-infection fungal growth.

### Map-based cloning of *Yr26*

**Fig. 2:**
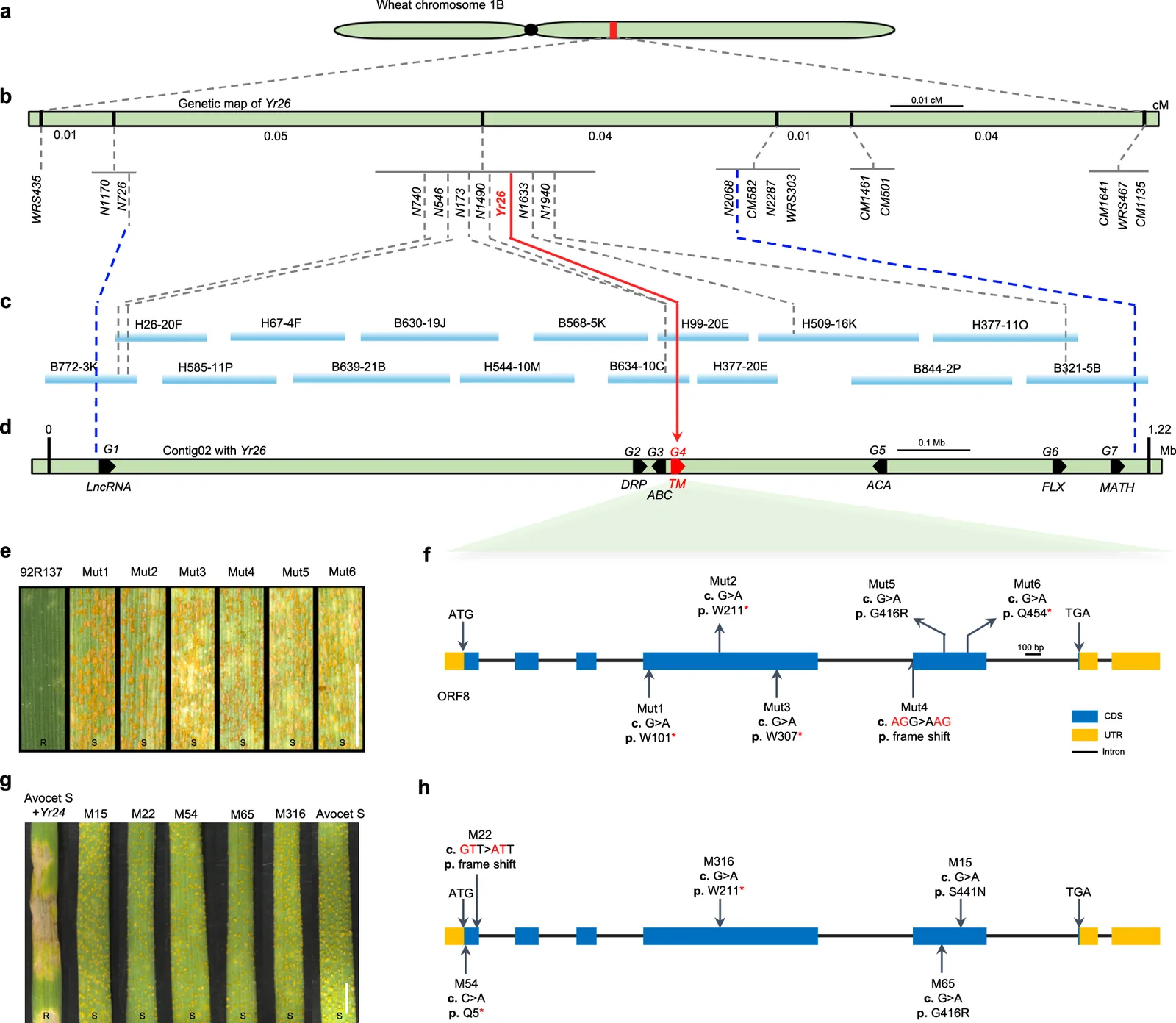
Map-based cloning of Yr26. **a** Schematic representation of *Yr26* (red rectangle) in wheat chromosome 1B. Solid black circle, centromere. **b** A high-resolution genetic linkage map of *YR26* constructed using 8046 F_2_ individuals from cross NIL-R/NIL-S. Numbers between DNA markers are genetic distances (cM, centiMorgans). **c** Relative positions of 15 bacterial artificial chromosome (BAC) clones covering the *Yr26* target interval. Blue and gray dotted lines link the BACs with markers in the genetic map. Red line represents the *YR26* position in the BAC clones. Light blue bars represent each BAC clone. Characters consisting of a letter and number above bars are IDs of corresponding BAC clones. **d** Contig02 in 92R137 constructed by 15 BAC clones. Length of contig02 is 1.22 Mb. Pentagons represent transcripts (*G1*-*G7*) identified using RNA-seq data from 92R137. LncRNA, long non-coding RNA; DRP, DNA-directed RNA polymerase subunit beta; ABC, ATP-binding cassette A1, ACA Adenylyl cyclase-associated protein, FLX Protein FLX-like 3, MATH MATH domain containing protein. *Yr26* candidate gene *G4* is highlighted in red. **e** Reactions of 92R137 and six independent susceptible mutant lines. R resistant, S susceptible. Scale bar, 1 cm. **f** Gene structure of *Yr26* candidate gene *G4*. Positions in the corresponding deoxyribonucleotide and amino acid changes in the six loss-of-function mutants (*Yr26*) are indicated. c cDNA, p protein. *; terminated translation. **g** Reactions of Avocet S + *Yr24* and five independent susceptible mutant lines. R resistant, S susceptible. Scale bars, 1 cm. **h** Gene structure of *Yr24* candidate gene *G4*. Positions in the corresponding deoxyribonucleotide and amino acid changes in the five loss-of-function mutants (*Yr24*) are indicated. c cDNA, p protein. *; terminated translation.

To clone *Yr26*, we developed an F_2_ population (8,046 plants) derived from a cross of NIL-R and NIL-S and identified 31 recombinants using KASP markers *WRS435* and *WRS312*^31^ (Supplementary Data 2). Using newly developed markers, the *YR26* locus was subsequently narrowed to a 0.002 cM interval (326.01-327.35 Mb, IWGSC CS RefSeq v1.0 flanked by markers *N726* and *N2068* (Fig. 2a, b, Supplementary Fig. 1a, b). Further refinement of the *Yr26* candidate interval was precluded by suppression of recombination within the mapped region. All remaining recombination events were asymmetrically clustered: on the proximal flank, all events mapped within a ~ 14 kb interval between markers *N726* and *N740*; on the distal flank, all events mapped within a ~ 127 kb interval between markers *N1940* and *N2068* (Supplementary Fig. 1c). No recombination was detected across the entire candidate interval spanning 326.01–327.35 Mb IWGSC CS RefSeq v1.0. This asymmetric clustering, together with the absence of recombination across the physical interval is characteristic of centromeric recombination suppression. Structural variation (e.g., chromosomal inversion) was excluded as an alternative explanation, as high collinearity in this region is preserved across diverse wheat genome assemblies (Supplementary Fig. 2). Consequently, the *Yr26* interval could not be resolved beyond 0.002 cM by genetic mapping alone.

Screening of the *Yr26*-carrying wheat line 92R137 BAC library yielded a 1.22 Mb contig02 assembled from 15 BAC clones covering the candidate region of *Yr26* (Fig. 2c). Annotation based on 92R137 transcriptomic data from *Pst*-infected leaf tissues identified seven transcripts (*G1*-*G7*) (Fig. 2d). *G1* was a long non-coding RNA (LncRNA) and *G2*-*G7* corresponded to the genes *TraesCS1B01G179900* (DNA-directed RNA polymerase subunit beta, DRP), *TraesCS1B01G351900LC* (ATP-binding cassette A1, ABC), *TraesCS1B01G180000* (unidentified), *TraesCS1B01G352100LC* (Adenylyl cyclase-associated protein, ACA), *TraesCS1B01G180300* (Protein FLX-like 3, FLX), and *TraesCS1B01G180400* (MATH domain containing protein, MATH) in CS (Supplementary Data 3). DNA and amino acid variations in these genes were then analyzed using RNA-seq and DNA re-sequencing data from the parental lines. There was no variation in *G1* and *G6* relative to the parental genotypes; *G2* had three SNPs, none of which caused an amino acid change; and *G3* had a missense mutation. Although *G5* and *G7* carried a common missense mutation, 92R137 and NIL-R had the same DNA sequence as CS, which was susceptible to CYR32 at the seedling stage and lacked *Yr26* linked DNA markers. *G4*, a transcript that does not exist in IWGSC CS RefSeq v1.0 (Supplementary Data 3), shared only 51.31% nucleotide sequence identity with *TraesCS1B01G180000*, and the *yr26* allele was not expressed in YM158 or NIL-S based on RNA-seq data. A MutRNAseq analysis showed that all six ethylmethane sulfonate (EMS)-caused susceptible mutants carried SNPs in *G4* (Supplementary Fig. 3). In contrast, the other six genes in the *Yr26* interval were unchanged compared with wild type 92R137. The mutations in *G4* were confirmed by polymerase chain reaction (PCR) and subsequent Sanger sequencing. We therefore identified *G4* as the sole candidate gene for *Yr26*.

We performed isoform sequencing of 92R137 to determine the full-length coding sequence (CDS) of *G4* using the PacBio long-read sequencing platform. A total of four *G4* isoform variants (hereafter, *IF1* – *IF4*) were identified (Supplementary Fig. 4). The dominant isoform *IF1* (90.48% abundance) encoded the longest open reading frame (ORF8, 481 amino acids) (Supplementary Fig. 5). All six susceptible mutants had SNPs that caused changes in the protein (Fig. 2f), including premature stop codons (Mut1-3, Mut6), a splice site alteration (Mut4), and a Gly416Arg substitution (Mut5), confirming *ORF8* as the functional *Yr26* CDS.

### MutChromSeq and MutRNAseq-based cloning of *Yr24*

Candidate gene identification for *Yr24* was performed by MutChromSeq in parallel with *Yr26* cloning using five susceptible mutants generated in a population of EMS-treated Avocet S + *Yr24* (Fig. 2g). In MutChromSeq analysis of chromosome 1B sequences of the wildtype and mutant lines, three mutants (M15, M22 and M316) had carried SNPs in *TraesCS1B01G179900*, *TraesCS1B01G351900LC*, *TraesCS1B01G180000*, *TraesCS1B01G352000LC*, and *TraesCS1B01G322800LC*. To refine the candidate list, whole-transcriptome sequencing was performed on the above three mutants, along with two additional mutants, M54 and M65. Comparative single nucleotide variant (SNV) analysis revealed that only *TraesCS1B01G180000* consistently harbored mutations across all five mutant lines.

Based on the RNA-seq data, the CDS of *TraesCS1B01G180000* was reconstructed, and inspection of the CDS identified a serine-to-asparagine substitution (S441N) in M15, a splice site alteration in intron1 in M22, a premature stop codon (Q5*) in M54, a glycine-to-arginine substitution (G416R in common with Mut5) in M65, and a tryptophan-to-stop substitution (W211*) in common with Mut2 in M316 (Fig. 2h). Collectively, the presence of independent, functionally disruptive mutations affecting conserved amino acid residues, together with the genomic localization, strongly supported *TraesCS1B01G180000* as the candidate gene for *Yr24*. Sanger sequencing confirmed that the 2945 bp coding region, 2099 bp promoter, and 758 bp terminator sequences of *TraesCS1B01G180000* in 92R137 and Avocet S + *Yr24* were identical, demonstrating that *Yr24* and *Yr26* are the same gene.

### Functional validation of *Yr26*

**Fig. 3:**
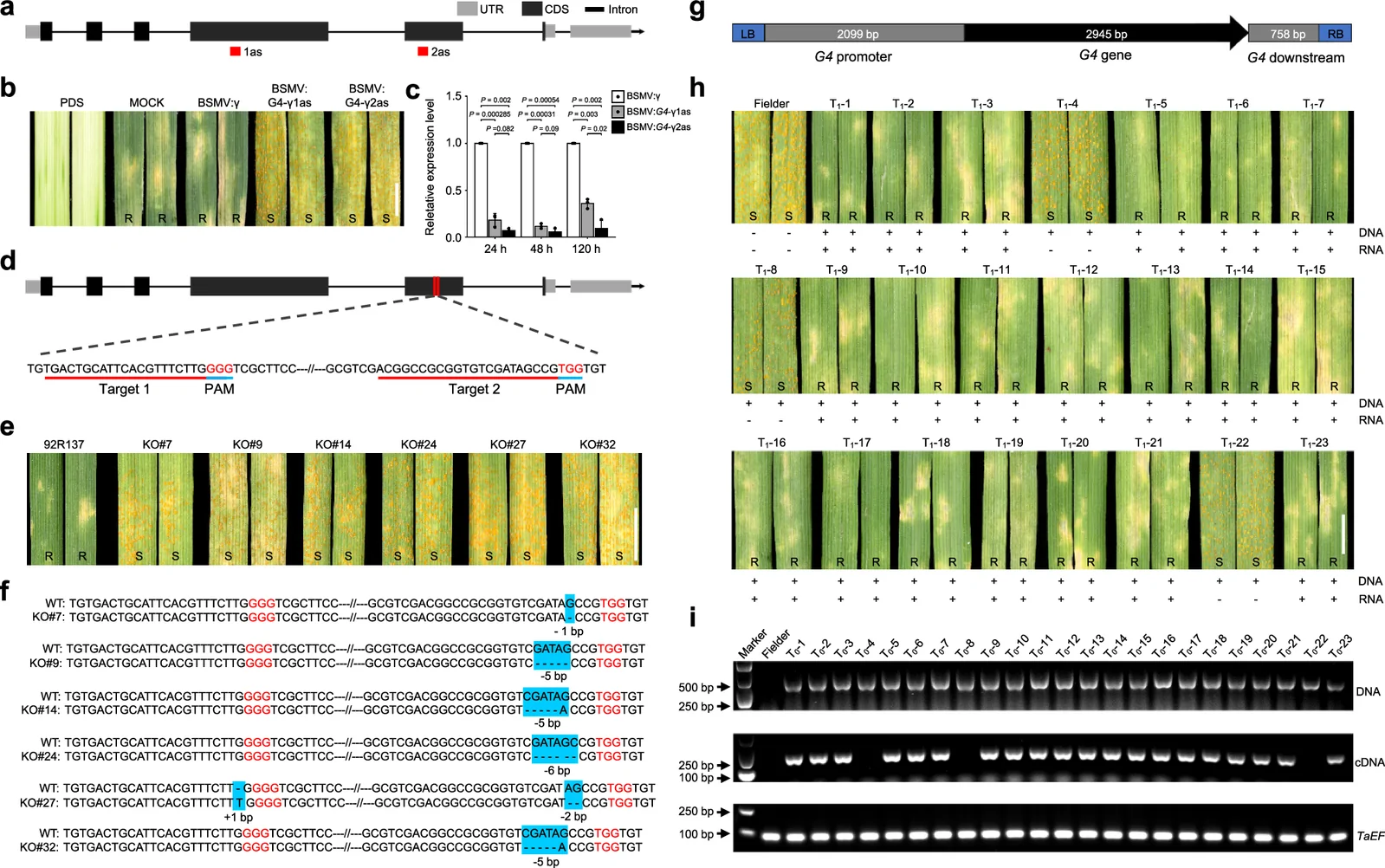
BSMV-mediated gene silencing, CRISPR/Cas9-mediated targeted mutagenesis and transgene complementation of Yr26. **a** Details of the two independent target segments (1as and 2as) for silencing *G4* by barley stripe mosaic virus (BSMV)-based virus-induced gene silencing (VIGS). **b** Representative VIGS phenotypes. Wheat leaves were inoculated with BSMV:*TaPDS*-γas (positive control for silencing), BSMV:γ (empty vector control), BSMV: *G4* -γ1/2as (two independent silencing constructs that target *G4*), and 1× FES buffer (mock), respectively. BSMV:*TaPDS*-γas phenotypes were recorded at 10 dpi. Reactions of *G4*-knockdown, mock-inoculated and control plants to *Pst* race CYR32 were recorded at 14 dpi. R, resistant, S susceptible. Scale bars, 1 cm. **c** Quantification of silencing efficiency for *G4* in VIGS plants. *TaEF-1α* was used for normalization. The transcript level of *G4* in the control leaves at 0 hpi was set as 1. Data are means ± s.d. (*n* = 3 biological replicates); each replicate as a single point. Asterisks indicate significant differences compared to the control (BSMV:γ) as assessed by two-tailed Student’s *t* tests (rstatix package v0.7.3 in R v4.2.0). *P* > 0.05, n.s. Randomization or blinding was applied; no data excluded. **d** Two CRISPR/Cas9 targets for *G4* knockout (KO). PAM motifs (5’-NGG-3’) in red and underlined in blue; target sequences are underlined in red. **e** Responses of homozygous mutant lines and 92R137 to CYR32. R resistant, S susceptible. Scale bars, 1 cm. **f** Genotypes of homozygous T_1_ mutants. PAM motifs in red; mutations in blue shading. –/+; deletion/insertion. **g** Construct for transformation of *G4*. The 5802-bp genomic DNA fragment consisted of the promoter, gene and downstream regions of *G4*. LB/RB, left/right border. **h** Reactions of T_1_ transgenic families and Fielder control in response to CYR32. +/-, presence/absence of *G4*. R, resistant; S, susceptible. Scale bars, 1 cm. **i** Detection of *G4* in T_0_ transgene plants using agarose gel electrophoresis. Fielder and *TaEF-1α* gene as controls. *n* = 3 biological replicates. White band indicates G4 presence. Source data are provided as a Source Data file.

Two *G4*-specific fragments were selected for gene silencing by barley stripe mosaic virus (BSMV) to further validate functionality of the candidate gene (Fig. 3a). Mild chlorotic mosaic symptoms appeared on BSMV-inoculated plants, whereas BSMV: *T. aestivum Phytoene Desaturase* (*TaPDS*)-as-infected plants exhibited strong photobleaching at 10 dpi (Fig. 3b), indicating that the BSMV-mediated gene silencing system was functioning correctly. The fourth leaf of each wheat plant was then challenged with an isolate of *Pst* race CYR32. At 14 dpi, control plants (mock-treated and BSMV:*γ*-infected) maintained HR without sporulation, whereas BSMV:*G4*-*γ*1/2as-silenced plants showed abundant uredinial development and profuse sporulation (Fig. 3b). Quantitative reverse transcription-PCR (qRT-PCR) verified effective *G4* knockdown (>50% reduction, *P* < 0.001) (Fig. 3c). To establish causality, we produced *G4* knockout lines by clustered regularly interspaced short palindromic repeat (CRISPR)-associated protein 9 (Cas9) gene-editing using two single-guide RNAs (sgRNAs) targeting different exonic regions (Fig. 3d). Among 31 transgenic plants, six had homozygous or biallelic frameshift mutations (1 to 6 bp indels) (Fig. 3e, f). T_1_ generation mutants were susceptible to stripe rust, indicating that knockout of *G4* caused loss of resistance.

Transgenic complementation assays were conducted to confirm that *G4* was sufficient for resistance. A 5.8-kb genomic fragment encompassing the native promoter, coding sequence, and terminator of *G4* was introduced into susceptible cv. Fielder by *Agrobacterium*-mediated transformation (Fig. 3g). Twenty-three independent T_0_ plants were obtained, 20 expressing *G4* and 3 null plants identified based on *G4*-specific DNA and cDNA markers (Fig. 3h, i). The *G4*-positive T_1_ lines were resistant whereas the null lines and wild type Fielder were susceptible (Fig. 3h). These experiments confirmed that *G4* encodes the functional Yr26 resistance protein.

### Race-specific resistance and expression dynamics of *Yr26*

**Fig. 4:**
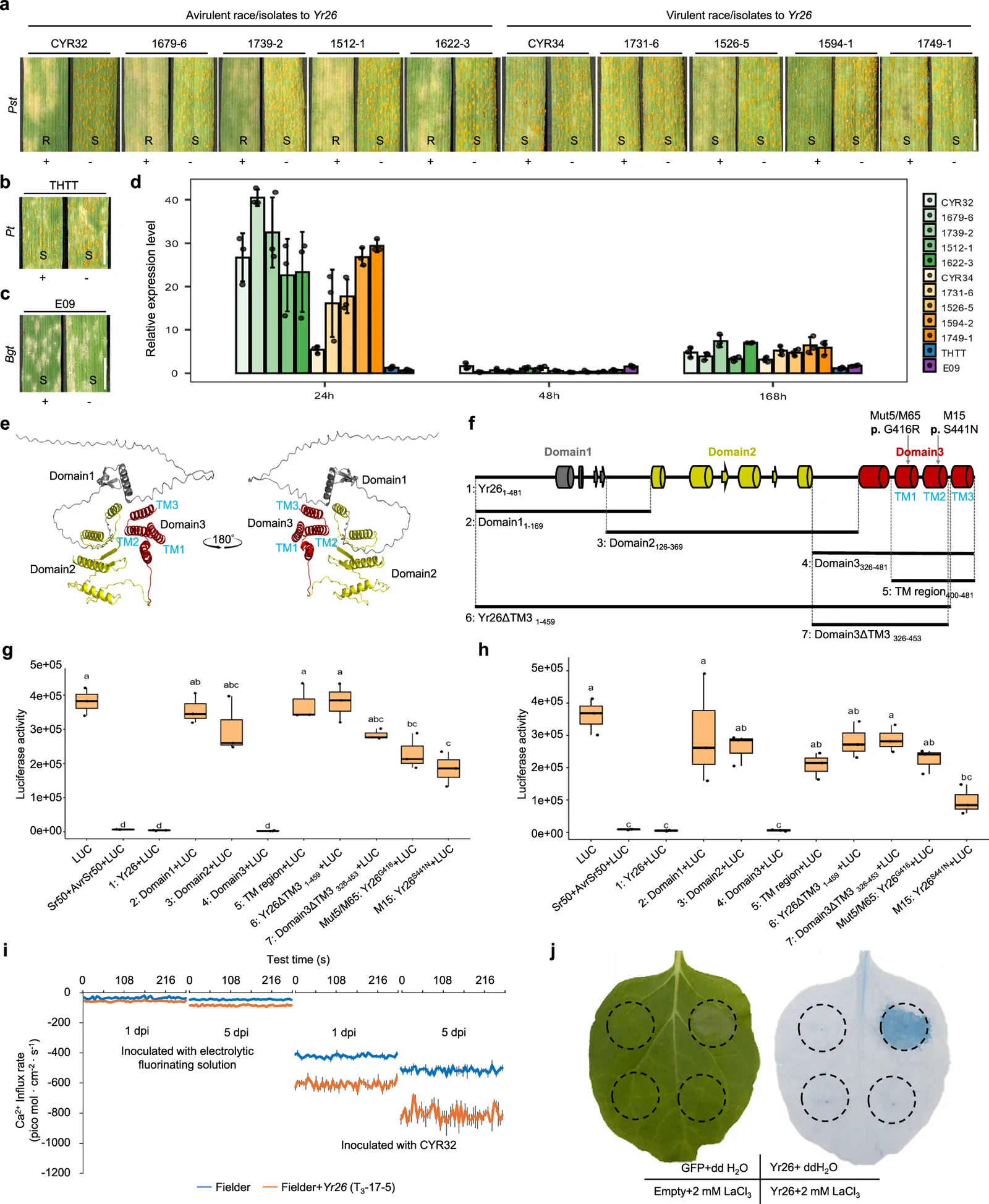
Race-specific resistance, expression and protein structure prediction of Yr26, and associated cell necrosis. Responses of the transgenic line T_3_-17-5 (Fielder + *Yr26*) and WT Fielder to ten *Pst* races/isolates (**a**), leaf rust (*Pt*) race THTT (**b**), and powdery mildew (*Bgt*) isolate E09 (**c**). “+”, T_3_-17-5; “–”, Fielder. R resistan, S susceptible. Scale bar, 0.5 cm. **d** Transcript levels of *Yr26* during 0-168 hpi in response to *Pst*, *Pt*, and *Bgt* by qRT-PCR (mock as control). RQ = 2^−ΔΔCq^, normalized to *TaEF-1α*, with CYR32 at 0 hpi as calibrator. Means ± s.d. (*n* = 3 biological replicates); each replicate as a single point. Green/orange, avirulent/virulent *Pst*; Blue, *Pt* THTT; Purple, *Bgt* E09. **e** Yr26 structure predicted by AlphaFold v3.0. Gray, N-terminal disordered (Domain1); yellow, central parallel helix array (Domain2); red, C-terminal four-helix bundle (Domain3). TM1-3, transmembrane helices. **f** *Yr26* secondary structure. Cylinders, α-helices; arrows, β-sheets. Domain colors as above. Arrows mark two EMS mutations. Bars and “Δ“ indicate constructs/domain deletions for protoplasts assays. Luciferase activity in wheat protoplasts 16 h after co-transformation with indicated constructs and luciferase reporter, in resistant line 92R137 (**g**) and susceptible line Fielder (**h**). Co‑transformation of *Sr50* and *AvrSr50* as necrosis control. Means ± s.d. (*n* = 3); Box plots: median (central line), interquartile range (edges) and whiskers (minimum to maximum); each reaction as a single point. One‑way ANOVA with Tukey’s test (rstatix v0.7.3); *P* > 0.05, n.s. **i** Ca^2+^ influx in T_3_-17-5 and WT Fielder leaves infected with CYR32 at 1dpi and 5 dpi. Electrolytic fluorinating solution as mock. Rates monitored for ~5 min per treatment by NMT. Data are mean (blue/orange lines) ± s.d. (black bars; *n* = 4); *P* > 0.05, n.s. **j** LaCl₃ suppresses Yr26-induced cell death in *N. benthamiana*. Agrobacteria carrying GFP, empty vector, and *Yr26* infiltrated; 8 h later, water or 2 mM LaCl₃ were applied. Trypan blue staining at 2 dpi (blue = dead). Yr26 alone induced death, blocked by LaCl₃; no death in controls. *n* = 3. Randomization/blinding; no data excluded. Source data are provided as a Source Data file.

Among 166 *Pst* isolates collected from the field in China from 2023 to 2025, 26 (15.66%) were virulent (IT = 7–9) on 92R137 (Supplementary Data 4), indicating that *Yr26* confers race-specific resistance. Homozygous resistant T_3_ transgenic line T_3_-17-5 (Fielder+*Yr26*) and wild-type Fielder were inoculated with 10 *Pst* isolates (including races CYR32 and CYR34) where five each were avirulent and virulent on 92R137, as well as *P. triticina* (*Pt*, the leaf rust pathogen) race THTT and *Blumeria graminus* f. sp. *tritici* (*Bgt*, the powdery mildew pathogen) isolate E09 (Fig. 4a–c). 92R137 and Guinong22 (donor of *YrG22*) were susceptible to *Pst* race CYR34. Fielder was susceptible to all tested isolates. T_3_-17-5 was resistant only to the five *Pst* isolates avirulent on 92R137, but susceptible to virulent *Pst* isolates, *Pt* race THTT, and *Bgt* isolate E09, confirming that *Yr26* confers race-specific resistance.

Transcriptional profiling of 92R137 infected with race CYR32 revealed abundant *Yr26* transcripts compared to mock controls (Supplementary Fig. 6). Real-time qRT-PCR confirmed that *Yr26* was significantly upregulated at both 24 and 168 hpi upon *Pst* infection, regardless of avirulence or virulence, whereas no significant induction was observed after inoculation with *Pt* or *Bgt* (Fig. 4d). These findings established *Yr26* as a *Pst*-specific inducible resistance gene.

### Structural features, subcellular localization, and hypersensitive response of Yr26

Sequence comparison of the Yr26 protein against all publicly available protein structure databases using BlastN and BlastP produced no hit. Two or three transmembrane (TM) structures were predicted in the C-terminus of the Yr26 protein using TMHMM v2.0 and Protter (Supplementary Fig. 7), implying that Yr26 is a TM protein. Structural predictions of Yr26 using AlphaFold v3.0 revealed an N-terminal disordered region (Domain1), a bundle-helix arrangement in parallel structure in the middle region (Domain2), and a C-terminal four-helix bundle structure (Domain3) (Fig. 4e, f). The predicted template modeling score (pTM = 0.18) and the highest ranking of 0.57 for the model indicated low to moderate model reliability at the global fold level (Supplementary Fig. 8). Most residues exhibited very low confidence (pLDDT < 50). In contrast, residues within Domain3 showed relatively higher confidence (pLDDT > 50), indicating that this region is comparatively well-defined.

Subcellular localization analysis revealed the presence of the full-length Yr26 protein in the plasma membrane, confirming that it is a TM protein (Supplementary Fig. 9). The Domain3 fragment was also localized to the plasma membrane, whereas the Domain1+ Domain2 fragment was detected in the cell membrane, cytoplasm, and nucleus, indicating that the Domain3 region harboring the predicted TM structures is necessary and sufficient for proper plasma membrane localization (Supplementary Fig. 9).

To investigate the mechanistic link between Yr26 and a hypersensitive reaction (HR), we conducted cell death assays in wheat protoplasts from resistant line 92R137 and susceptible line Fielder. As expected, co-transformation of *Sr50* and *AvrSr50* (positive control) induced evident necrosis in wheat protoplasts, whereas no necrotic phenotype was observed in protoplasts transformed with the LUC alone (negative control) (Fig. 4g, h). Full-length Yr26 or Domain3 alone triggered strong necrosis in protoplasts of two wheat varieties, whereas removal of the TM3 helix eliminated this cell death induction (Fig. 4g, h). Similar results were obtained in *N. benthamiana* leaves (Supplementary Fig. 10b, c). Consistent with this, among the EMS-induced loss-of-function mutants, Mut6 carries a premature stop codon that truncates 27 C-terminal residues encompassing part of the TM3 region, abolishing resistance (Fig. 2f). Two additional missense mutations in Domain3-G416R (Mut5/M65) and S441N (M15) also eliminated or markedly reduced cell death activity (Fig. 4g, h; Supplementary Fig. 10b, c). These independent genetic lesions, obtained through unbiased mutant screens, converge on Domain3 and collectively demonstrate that an intact C-terminal four-helix bundle structure is essential for Yr26-mediated HR and pathogen containment.

Given that calcium ions (Ca^2+^) function as classic early second messenger in the plant immune signaling pathway and play a crucial role in regulating cell death, we investigated the role of Ca^2+^ in Yr26-mediated immunity. Ca^2+^ influx was measured in mesophyll tissue of the *Yr26* transgenic line T_3_-17-5 and WT Fielder at 1 and 5 dpi with *Pst* race CYR32. In mock-inoculated controls (fluorinated liquid), the Ca^2+^ influx rate in T_3_-17-5 (−48.8 and −88.4 picomol cm^−2^ s^−1^ at 1 and 5 dpi, respectively) was slightly higher than that in the WT (−38.4 and −59.9 picomol cm^−2^ s^−1^), but both remained at relatively low levels (Fig. 4i and Supplementary Data 5). Upon CYR32 inoculation, Ca^2+^ influx rate increased dramatically in both T_3_-17-5 (−519.3 and −813.0 picomol cm^−2^ s^−1^ at 1 and 5 dpi) and the WT (−425.4 and −612.5 picomol cm^−2^ s^−1^), indicating that *Pst* infection strongly induces Ca^2+^ influx. Compared with the WT, T_3_-17-5 exhibited approximately 22.08% and 32.73% higher Ca²⁺ influx rates at 1 dpi and 5 dpi, respectively, suggesting that *Yr26* activates Ca²⁺ influx upon *Pst* challenge. Furthermore, co-application of the Ca^2+^ channel blocker LaCl_3_ (2 mM) during transient expression of *Yr26* in *N. benthamiana* abolished the cell death typically triggered by Yr26, confirming that Ca^2+^ plays an essential role in the Yr26-mediated immune response (Fig. 4j).

### Natural variation and diagnostic marker for *Yr26*

**Fig. 5:**
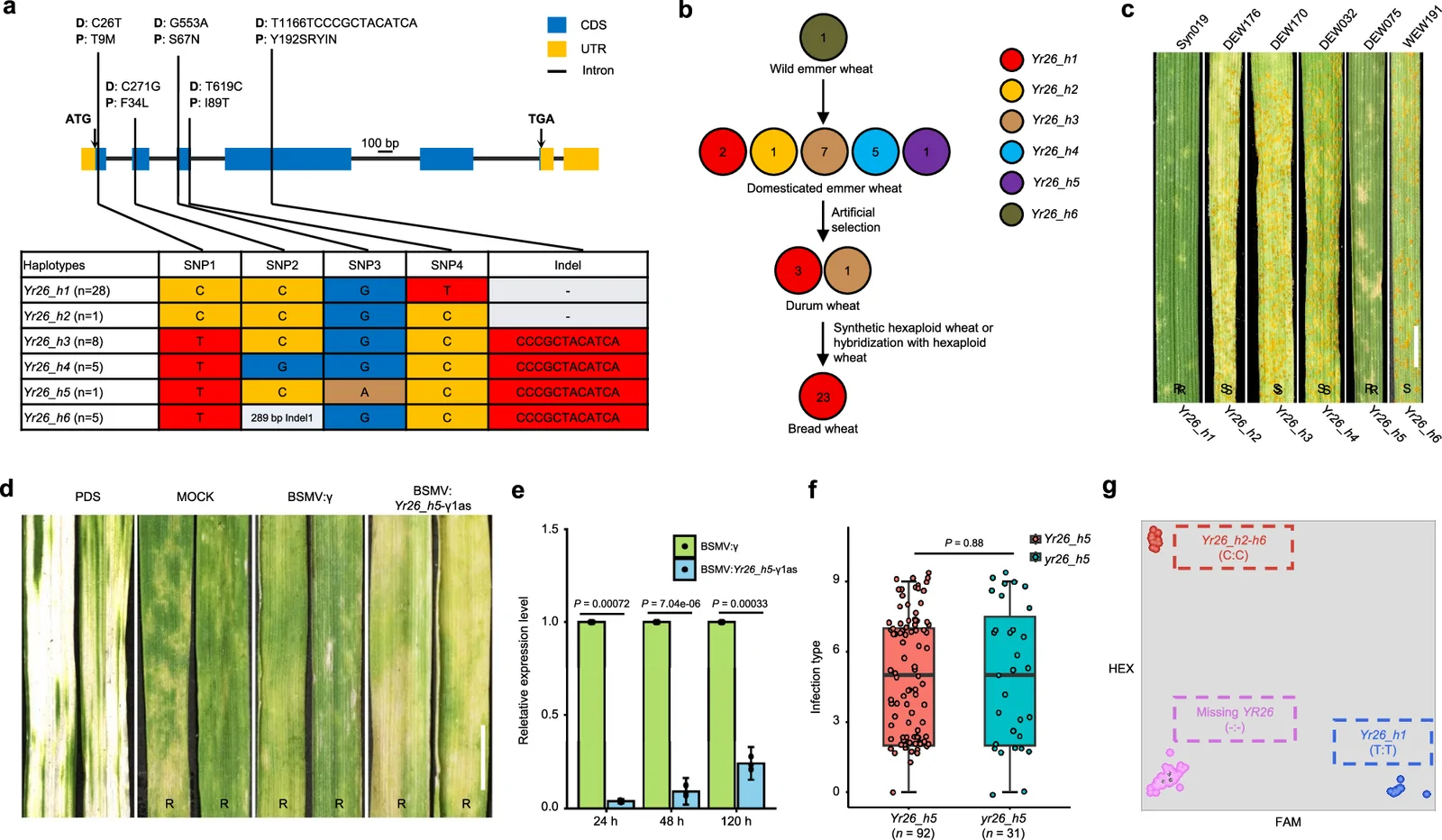
Natural variation and YR26 haplotypes. **a** Natural variations and haplotypes of *YR26* in a wheat accession panel consisting of 392 wild emmer wheat (WEW), 225 domesticated emmer wheat (DEW), 294 durum wheat accessions, and 105 bread wheat genotypes. Arrows indicate the start and stop codons of *YR26*. *Yr26_h1 – _h6*, six haplotypes. SNP1-4 and Indel, the five variations among *Yr26_h1 – _h5*. D; DNA; P, protein; -, missing. **b** Distribution of *Yr26* haplotypes in WEW, DEW, durum, and bread wheat. Numbers in the colored circles represent the numbers of accessions for each haplotype. **c** Responses of exampled accessions from each haplotype to *Pst* race CYR32. R resistant, S susceptible. Scale bar, 1 cm. **d** Representative images showing results of the VIGS experiment. Wheat leaves inoculated with BSMV:*TaPDS*-γas (positive control for silencing), BSMV:γ (empty vector control), BSMV:*Yr26_h5*-γ1as (silencing constructs that target *Yr26_h5*), and 1× FES buffer (mock), respectively. Phenotypes for BSMV:*TaPDS*-γas plants were recorded at 10 dpi. Reactions of *Yr26_h5*-knockdown, mock-inoculated, and control plants to *Pst* race CYR32 were recorded at 14 dpi. R resistant, S susceptible. Scale bars, 0.5 cm. **e** Quantification of silencing efficiency for *Yr26_h5* in VIGS plants. Data are means ± s.d. (*n* = 3 biological replicates); each replicate as a single point. *TaEF-1α* was used for normalization. The transcript level of *Yr26_h5* in the control leaves at 0 hpi was set as 1. Randomization or blinding applied; no data excluded. **f** Genotypic effects of *YR26_h5* on CYR32 infection types in the DEW075/D026 F₂ population. The sample size (*n*) was 92 and 31 for genotypes *Yr26_h5* and *yr26_h5*, respectively. Box plots show median (central line), interquartile range (edges) and whiskers (minimum to maximum); dots represent individual data points. **g** Genotyping of the *Yr26_h1*-specific diagnostic KASP marker in a diversity panel. Statistical significance was determined using two-tailed Student’s *t* tests (rstatix v0.7.3). *P* > 0.05, n.s. Source data are provided as a Source Data file.

To confirm the relationship of *Yr26* with *YrCH42* and *YrG22*, we cloned the *G4* sequence including coding region, promoter and terminator sequences from the respective carrier lines Chuanmai 42 (*YrCH42*) and Guinong 22 (*YrG22*). The *G4* sequences in 92R137, Avocet S + *Yr24*, Chuanmai 42, and Guinong 22 were identical, confirming that these lines carry the same stripe rust resistance gene. A *YR26* locus-specific marker *DM2* based on DNA sequence variation between publicly available assembled wheat genomes (Supplementary Figs. 11 and 12) was designed and screened on a diverse panel of 392 wild emmer (WEW), 225 cultivated emmer (DEW), 294 durum, and 105 accessions of synthetic wheat genotypes with durum parents. Forty-eight accessions were identified as carriers of *YR26* (Supplementary Data 6). Sanger sequencing of *YR26* in these accessions identified six haplotypes, including the standard *Yr26_h1* and five new haplotypes, designated *Yr26_h2 – _h6* (Fig. 5a). Four SNPs (SNP1-4) and one 12 bp indel were identified among *Yr26_h1 – _h6*, and a 289 bp indel encompassing SNP2 and four additional SNPs distinguished *Yr26_h1* from *Yr26_h6* (Supplementary Fig. 13). *Yr26_h6* was exclusively present in WEW accessions. *Yr26_h1 –_ h5* were present in DEW but absent in WEW accessions. *Yr26_h1* was exclusively present in durum and hexaploid accessions known to be synthetic wheat lines or derivatives, or directly transferred to hexaploid wheat from durum (Fig. 5b).

To confirm a stripe rust resistance function of lines with each *Yr26* haplotype, the stripe rust responses of the 48 genotypes carrying the *YR26* locus were determined using *Pst* races CYR32 (avirulent *Yr26*) and CYR34 (virulent). However, the heterogeneous backgrounds involving other resistance genes possibly masked the effect of each haplotype. Nevertheless, all *Yr26_h1* containing accessions were resistant to race CYR32 and mostly susceptible to CYR34. Lines with *Yr26_h2 – _h4* and *Yr26_h6* were mostly susceptible to both races, indicating that these haplotypes do not confer the resistance specificity associated with *Yr26_h1* (Supplementary Data 6, Fig. 5c). The single accession carrying *Yr26_h5* (DEW075) was resistant to both races, however, based on a single accession, the contribution of *Yr26_h5* to resistance could not be conclusively determined (Fig. 5c). A VIGS experiment was conducted on DEW075 using BSMV:*G4*-γ1as vector to test whether *Yr26_h5* functions in resistance. No significant difference in stripe rust response to race CYR32 was observed between the silenced plants and the mock or control groups (Fig. 5d, e). In parallel, an association analysis was performed using an F_2_ population derived from a cross between DEW075 and the susceptible accession D026. Based on the *YR26* diagnostic marker *DM2*, no significant difference in stripe rust reaction was detected between the *Yr26_h5* (*n* = 92) and *yr26_h5* (*n* = 31) lines (Fig. 5f and Supplementary Data 7). These findings indicate that *Yr26_h5* was not required for resistance to CYR32 in DEW075, and the broad-spectrum resistance observed in this accession was likely conferred by one or more different genes.

*Yr26_h1* and *Yr26_h2*, which are associated with contrasting stripe rust resistance responses, differ only at SNP4 (Fig. 5a). This single nucleotide polymorphism results in an amino acid substitution from isoleucine (I) to threonine (T), indicating that SNP4 is likely the key mutation conferring resistance in *Yr26_h1*. Based on SNP4 we designed and tested a *Yr26_h1* allele-specific marker in 2541 Chinese bread wheat accessions, among which *Yr26_h1* was detected in 148 genotypes (Fig. 5g). These latter genotypes were mainly derived from cultivars grown in Guizhou and Sichuan provinces (90.5%) where 92R137 (*Yr26*), Chuanmai 42 (*YrCH42*) and Guinong 22 (*YrG22*) were widely used as resistance donors in breeding programs (Supplementary Data 8).

### Origin and evolution of *Yr26*

**Fig. 6:**
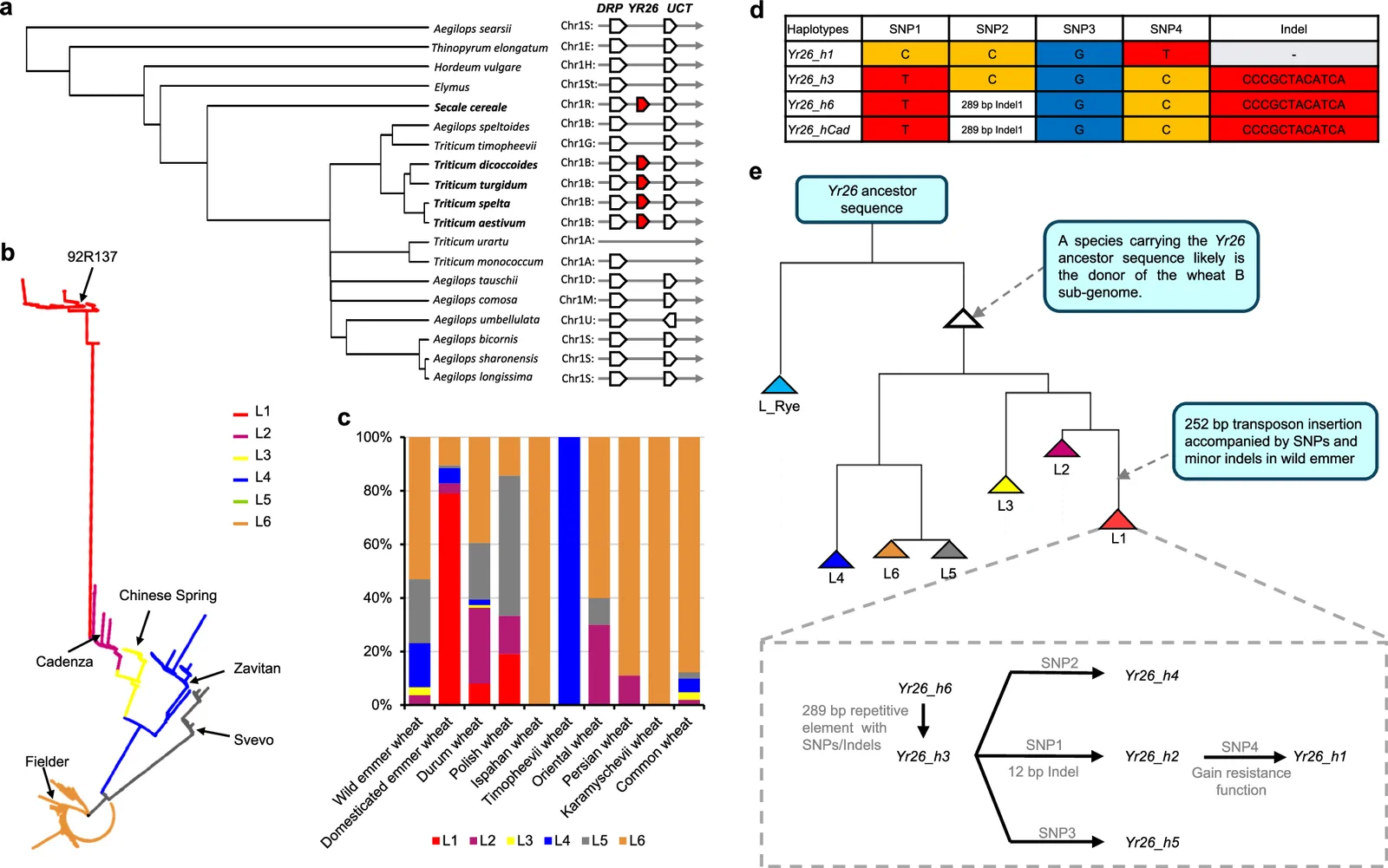
Evolution of YR26. **a** Micro-collinearity of the *YR26* region between wheat and its relatives. Phylogenetic tree shows the evolutionary relationship among different species (https://timetree.org/). *YR26* and its homologs are highlighted in red. *DRP* (DNA-directed RNA polymerase subunit beta) and *UCT* (ubiquitin carboxyl-terminal hydrolase) are high confidence genes flanking *YR26*. **b** Phylogenetic tree of 2,483 wheat accessions using SNPs in the *YR26* gene based on re-sequenced genotype data. The tree was constructed by the neighbor-joining method. L1-6 represent six independent lineages. Arrows indicate the position of six representative assembled accessions in the tree. **c** Distribution of the six lineages in different ‘taxonomic species’ of tetraploid and hexaploid wheat. **d** Genotypes of *YR26* haplotypes and *Yr26_hCad* at SNP1-4 and the 12‑bp-indel. *Yr26_hCad* is a *Yr26* homolog in wheat cultivar Cadenza. **e** Hypothesized origin of *Yr26_h1* evolving from an ancestral sequence. L_Rye, lineage of *Yr26* in rye. SNP1-4 and 12 bp Indel are the five variations found among *YR26* haplotypes.

*Yr26* is an orphan gene lacking detectable sequence similarity to all genes from outside the *Triticeae*, except for a potential homolog located at the *YR26* locus in wheat reference assemblies. To investigate its evolutionary origin, we performed comprehensive BLAST searches across 36,259 non-redundant genomes spanning plants, animals, fungi, bacteria, and archaea. As expected, homologs were identified in wheat relatives including wild and domesticated emmer, durum, and bread wheat as well as in chromosome arm 1RL of rye (*Secale cereale*) accessions Weining and Lo7 (Fig. 6a and Supplementary Data 9). This restricted phylogenetic distribution suggests the *YR26* ancestral sequence likely emerged before divergence of *S. cereale* and modern *Triticeae* crops (about 5.94 MYA). Furthermore, micro-collinear conservation at the *YR26* locus was observed across 19 *Triticeae* genomes and was flanked by evolutionarily stable genes *DRP* and *UCT*, demonstrating that *YR26* unlikely originated by horizontal gene transfer or chromosomal inversion, which are principal mechanisms for orphan gene emergence (Fig. 6a). The *YR26* homologs present in homeologous group 1 chromosomes of rye and B sub-genomes of polyploid wheat, but not in eight diploid grasses phylogenetically proximate to wheat, further support its recent emergence within the *Triticeae*. These findings suggest that the ancestral donor lineage of *YR26* may have undergone genomic extinction during *Triticeae* radiation.

It is beneficial to obtain as many independent intermediate homologs as possible for clarifying a gene’s origin. Therefore, we downloaded re-sequenced genotypic data of 342 tetraploid and 2141 hexaploid wheat accessions and aligned them to IWGSC CS RefSeq v2.1 (Supplementary Data 10). A phylogenetic tree constructed together with assembled wheat accessions using 149 SNPs divided the accessions into six main lineages (L1-6) (Fig. 6b) and at least one assembled wheat accession was grouped in each lineage, such that the public wheat genomes covered all six lineages of the *YR26* locus. 92R137 with *Yr26* was placed at L1 in the phylogenetic tree. L2 including assembled wheat Cadenza was closest to L1, suggesting that it was the precursor of L1. Both L1 and L2 were absent in wild emmer, but present in cultivated emmer, indicating that the *Yr26* functional allele first appeared in cultivated emmer or an unsampled wild emmer as suggested by the distribution of *Yr26*_*h1 –* _*h6* within the *Triticeae* (Fig. 6c and Supplementary Data 11).

To investigate the evolutionary trajectory of *Yr26*, we retrieved homologous sequences from assembled genomes of 47 diverse wheat cultivars for comparative analysis using cereal rye as an outgroup (Supplementary Fig. 14). Phylogenetic reconstruction revealed distinct clustering patterns: rye accessions Lo7 and Weining occupied the outermost branches, suggesting retention of ancestral sequence features. Synthetic wheat derived cultivars Chuanmai 42 and Chuanmai 104 formed a monophyletic clade with 92R137, indicative of shared evolutionary origins. The Cadenza genome, though not assembled to chromosome-level resolution, contained the closest homolog (*Yr26_hCad*, unannotated gene), localized to scaffold_037108 (20,674–23,121 bp) with 78.2% nucleotide identity to *Yr26* (Supplementary Data 9). *Yr26_hCad* belongs to L2 which is more closely related to L_Rye in evolution than L1-carrying *YR26* alleles, indicating that *Yr26_hCad* appeared earlier than *Yr26_h1 – _h6*.

Among *Yr26_h1 – _h6*, *Yr26_hCad* showed the highest sequence similarity with *Yr26_h6* and carried the same 289 bp indel 1 (from 215 to 503 bp), suggesting that *Yr26_h6* arose first (Supplementary Fig. 15). *Yr26_h6* shared identical genotypes at the variable sites distinguishing the *Yr26_h1 – _h5* haplotypes with *Yr26_h3*, indicating that *Yr26_h3* likely emerged subsequently (Fig. 6d). A comparative analysis of *Yr26_hCad*, *Yr26_h1* and *Yr26_h6* revealed a multi-stage evolutionary pathway (Supplementary Fig. 15). Firstly, *Yr26_hCad* served as the non-functional progenitor allele leading to *Yr26_h6* through a structural rearrangement involving indel 2 (positions 1,827 *–* 2,082 bp), which originated from a transposable element inserted via genomic transposition, accompanied by SNPs and minor indels in wild emmer (Fig. 6e). Then, a de novo start codon arose by ATT to ATG mutation, enabling protein translation (Supplementary Fig. 15). Thirdly, indel 1 (from 215 to 503 bp) arose from tandem duplication of a downstream 289 bp segment with 70.07% identity (Supplementary Fig. 16). Fourthly, functional refinement based on sequential nucleotide changes generated transitional alleles (*Yr26_h3*: C to G [SNP2]; *Yr26_h5*: G to A [SNP3]; *Yr26_h2*: T to C [SNP1] + 12-bp deletion). Finally, a C to T substitution at SNP4 in the *Yr26_h2* background likely represents the key gain-of-function mutation, giving rise to the resistant *Yr26_h1* allele (Fig. 6f).

## Discussion

Here, we isolated the stripe rust resistance gene *Yr26* by a combination of map-based cloning, MutChrom, and MutRNA-seq approaches, and validated its function using VIGS, mutational analysis, CRISPR-Cas9 knockout, and transgenic complementation. We demonstrate that *Yr26* encodes a transmembrane protein whose C-terminal four-helix bundle domain is essential for induction of a hypersensitive response. Furthermore, we show that *Yr26* is a recently evolved orphan gene within the *Triticeae*, define its stepwise evolutionary trajectory from a non-functional ancestral sequence, and develop a diagnostic marker for breeding applications.

*Yr26* was fine-mapped to a recombination-suppressed interval in the near centromeric region of chromosome 1B. In such regions, combining target region assembly with mutant analysis has proven effective for gene isolation. For example, a chromosome-scale assembly of the South African bread wheat cultivar Kariega, combined with EMS-induced mutational analysis, enabled the isolation of *Yr27*^15^, which encodes a coiled-coil NLR immune receptor. In this study, we assembled the *Yr26* target region using a BAC-by-BAC strategy, as demonstrated for *Sr33*^33^. Transcript-based annotation and RNA-seq analysis of six independent EMS-derived susceptible mutants identified *G4* as the sole candidate gene. In parallel, MutChromSeq analysis of five susceptible mutants derived from the Avocet S + *Yr24* background independently identified the same gene, confirming that *Yr24* and *Yr26* are identical. More broadly, mutant-based cloning strategies have become invaluable for isolating resistance genes in recombinationally inert genomic regions, particularly in crops with complex or incomplete reference genomes. Notable examples include MutRenSeq for *Yr5*^20^, MutIsoSeq for *Yr9*^14^, and sequencing trait-associated mutations (STAM) for *Yr10*^24^ in polyploid wheat. The cloning of *Yr26* further demonstrates the power of integrating genome assembly with systematic mutant analysis to overcome the challenges posed by centromeric recombination suppression.

Structural analysis identified *Yr26* as a resistance gene harboring two to three transmembrane (TM) helices, thereby differing from classical NLR-type resistance genes_._ Emerging evidence increasingly implicates TM proteins as critical components in plant disease resistance. In *Arabidopsis*, the broad-spectrum mildew resistance protein Rpw8^34^ and the *Plasmodiophora brassicae* resistance gene WeiTsing (WTS)^35^ both contain TM domains and localize to pathogen-derived membranes to restrict infection. In barley, the leaf rust resistance gene *Rph3*^36^ encodes a TM protein and is strictly induced by pathogenic fungi during infection. In wheat, several TM-domain resistance proteins have been characterized, including TaMlo^37^ (powdery mildew resistance), Lr34/Yr18/Sr57/Pm38^7^, Lr67/Yr46/Sr55/Pm46^9^ and WAI-B2^38^ (broad-spectrum multiple pathogen resistance), and Lr14a^39^ (leaf rust resistance). Additional TM-containing resistance proteins, such as Pm4^40^, Stb16q^41^, and WTK7-TM^23^, possess TM domains that are essential for their membrane localization and function. In this study, an EMS mutant (Mut6) carrying a premature stop codon upstream of the C-terminal TM helices produced a truncated protein lacking an intact Domain3, resulting in loss of resistance. This genetic evidence confirms that the TM domain is indispensable for Yr26 function. The identification of Yr26 further expands the repertoire of TM resistance proteins in wheat and underscores the central role of such proteins in plant-pathogens interactions.

Calcium ions (Ca²⁺) act as an essential second messenger in plant immune signaling, often functioning upstream of HR-associated cell death^42^. Recent studies revealed that several plant resistance proteins function as Ca²⁺-permeable channels or mediate Ca²⁺ influx to execute defense signaling. The NLR resistosome^43^, a pentameric protein complex formed upon effector recognition, directly mediates Ca²⁺ influx to trigger cell death. The *Arabidopsis* TM resistance protein WTS^35^ forms a pentameric cation channel permeable to Ca²⁺, as demonstrated by cryo-electron microscopy and electrophysiological analyses, and its channel activity is essential for immune signaling. In wheat, Lr14a has been implicated in Ca²⁺-dependent signaling^44^. Our functional analyses indicate that *Yr26*-mediated cell death is Ca²⁺-dependent. *Pst* infection induced a stronger Ca²⁺ influx in *Yr26*-transgenic wheat leaf tissue compared with wild-type controls, and co-application of the Ca²⁺ channel blocker LaCl₃ abolished *Yr26*-induced cell death in *N. benthamiana*. AlphaFold v3.0 predicted a four-helix bundle within Domain 3 that is necessary and sufficient for Yr26-mediated cell death and shows some topological resemblance to *WTS*^35^ transmembrane architecture. These observations raise the possibility that *Yr26* functions as part of an ion channel complex facilitating Ca²⁺ influx, analogous to WTS. However, the overall AlphaFold predicted template modeling score is low (pTM = 0.18), and direct structural and electrophysiological evidence is required to test this hypothesis. In addition to Domain3, AlphaFold v3.0 predicted an N-terminal disordered region (Domain1) and a central parallel helix bundle (Domain2), but both were predicted with low confidence. The contributions of Domain1 and Domain2 to *Yr26*-mediated resistance remain unknown and represent an important direction for future investigation.

Expression of *Yr26* was specifically induced by *Pst* infection, irrespective of whether the infecting race was virulent or avirulent. Transient overexpression of *Yr26* triggered cell necrosis in susceptible and resistant wheat, as well as in *N. benthamiana* leaves, suggesting that the cell death pathway activated by *Yr26* is functionally conserved across accessions and plant species. Although virulent *Pst* races induced *Yr26* expression, they failed to trigger HR, suggesting that HR is suppressed during compatible interactions, and may require additional pathogen effectors. Due to the dikaryotic nature of *Pst* and the technical challenges of genetic manipulation in this obligate biotroph, no avirulence effector corresponding to a *Yr* resistance gene has been cloned^45^. Therefore, identifying the Avr effector targeting *Yr26* will be essential for understanding the molecular basis of recognition and avirulence.

Large-scale genome alignments revealed that *Yr26* homologous sequences were present only in *Triticeae* species. No significant homology was detected across 36,259 non-redundant genomes spanning species, animals, fungi, bacteria, and archaea, indicating that *Yr26* is an orphan gene. Detailed evolutionary analyses indicate that *Yr26* evolved independently within the *Triticeae* lineage through a series of sequential genomic events, including the emergence of a novel start codon, transposable element insertion, tandem duplication, and accumulation of SNPs and indels. These modifications collectively transformed a non-functional ancestral sequence into a functional resistance gene. In recent years, the functional characterization of orphan genes in plant-pathogen interactions has gained increasing attention^46^. For instance, *TaFROG*, a *Pooideae*-specific orphan gene, is well-known in plant-pathogen interactions^47^. This gene is induced during the early response of wheat to deoxynivalenol toxin produced by *Fusarium graminearum* and interacts with *SnRK1* and NAC-like transcription factor *TaNACL-D1* to enhance host resistance to the fungus^48^. Another secreted orphan protein OSP24 from *F. graminearum* promotes *TaSnRK1α* degradation, weakening host resistance^49^. However, TaFROG competes with OSP24 for TaSnRK1α binding, stabilizing TaSnRK1α and enhancing resistance. In rice, the *Oryza*-specific orphan gene *Xio1* is induced by *Xanthomonas oryzae* pv. *oryzae* infection and confers resistance, albeit with growth penalties^50^. Another rice orphan gene, *Xa7*^51^, triggers resistance by perceiving *AvrXa7* through the transcription activator-like effector (TALE) binding element (EBE) in its promoter. *Xa7* is absent in most rice varieties but present in a related wild grass species. These findings illustrate how orphan genes diversify resistance to pathogens.

Evolutionary analysis and conserved micro-collinearity indicate that the ancestral donor lineage of *YR26* may have undergone genomic extinction during spread of the *Triticeae*. This hypothesis is consistent with the enigmatic evolutionary trajectory of the wheat B sub-genome. Genome sequences of five *Sitopsis* species of *Aegilops* revealed that the donor of the common wheat B subgenome is a distinct, and most likely extinct, diploid species that diverged from an ancestral B lineage progenitor closely related to the extant *Ae. speltoides* and *Am. muticum*^52^. Additionally, the presence of a *Yr26* homolog on cereal rye chromosome arm 1RL implies historical gene flow or a recent evolutionary relationship between the progenitors of rye and B subgenome donor species. These findings offer insights for future research on the donors of the B-subgroup genomes.

Genetic profiles of diverse accessions indicated that *Yr26* first emerged in domesticated emmer or an unsampled wild emmer lineage and was subsequently inherited by durum before being introgressed into bread wheat by breeders. Bread wheat progenitors have reservoirs of disease resistance genes, for example, *Yr15*^22^ and *Yr36*^8^ from wild emmer, stem rust resistance gene *Sr13* from cultivated emmer and durum wheat^53^, and powdery mildew resistance gene *Pm36*^23^ from wild emmer. The cloning of *Yr26* underscores the enduring value of cultivated emmer and durum wheat germplasm in expanding the genetic diversity of disease resistance in bread wheat.

Resistance genes with distinct protein structures often involve different immune mechanisms and stacking these genes is a promising strategy to achieve broad-spectrum resistance. For example, *Lr14a* encodes an ankyrin-repeat transmembrane domain protein^39^ that functions as a calcium-permeable channel to trigger cell death in guard cells to confer leaf rust resistance in wheat^44^. *Lr34*/*Yr18* encodes an ATP-binding cassette transporter against multiple fungal pathogens by mediating sinapyl alcohol transport and cell wall deposition and highlights the synergistic effect of TaCOMT-3B^54^. These two genes together with *Lr75*, which has not been cloned, provided broad-spectrum resistance in wheat cultivar Forno^39,55,56^. In wheat, rust resistance genes, such as *Yr15* encoding a tandem kinase^22^ and *Yr10* encoding a protein with a NAM and ZnF-BED domains^24,25^, were isolated as atypical NLR proteins. Here, *Yr26* was shown to produce a novel protein structure. Combining *Yr26* with these functionally diverse resistance genes represents an attractive strategy for achieving broad-spectrum rust resistance in future wheat breeding programs. In summary, we isolated the all-stage resistance gene *Yr26* that encodes a transmembrane protein and showed that it was a recently evolved orphan gene within the *Triticeae* lineage and delineate a multi-step trajectory through which a non-functional ancestral sequence acquired race-specific resistance function. The gene triggers a Ca²⁺-dependent hypersensitive response upon recognition of avirulent *Pst* isolates, and its C-terminal Domain3 is both necessary and sufficient for induction of cell death. These findings expand the repertoire of functionally validated disease resistance genes, provide evidence that orphan genes can evolve de novo to fulfill critical roles in plant-pathogen interactions, and offer a diagnostic marker for marker-assisted breeding. Future work aimed at elucidating the molecular recognition mechanism, determining the high-resolution structure, and combining *Yr26* with other resistance genes will contribute to development of durable stripe rust resistance in wheat.

## Methods

### Plant materials

The study utilized a panel of wheat lines, including cultivar Yangmai 158 (YM158), Chuanmai 42 (*YrCH42*), Guinong 22 (*YrG22*) and wheat lines 92R137, Avocet S + *Yr24* and Avocet S. Near-isogenic lines (BC_6_F_4_) NIL-R (+*Yr26*) and NIL-S (-*Yr26*) were derived from a cross between YM158 and 92R137. An F_2_ population of 8046 plants generated from cross NIL-R × NIL-S was used in genetic mapping. Susceptible controls included Fielder (the transgenic recipient), Xiaoyan 22, and Mingxian 169 (MX169). A diversity panel to explore alleles of *YR26* comprised 911 tetraploid accessions (including 392 wild emmer (*T. turgidum* ssp. *dicoccoides*), 225 cultivated emmer (*T. turgidum* ssp. *dicoccum*), 294 durum (*T. turgidum* ssp. *durum*)) and 105 synthetics or derivatives (Supplementary Data 6). A panel of 2541 Chinese breeding lines/cultivars was used in haplotype diversity analysis and marker validation of *Yr26* (Supplementary Data 8).

### Evaluation of stripe rust, leaf rust, and powdery mildew response

*Pst* races CYR32, CYR34 and 166 isolates collected from the field in China from 2023 to 2025 were reproduced on MX169 under controlled greenhouse conditions for determination of stripe rust responses (Supplementary Data 4). CYR32 and CYR34 are both prevalent *Pst* races, each with a frequency exceeding 10% in major wheat-growing regions. CYR32 is avirulent to *Yr26* and has been widely used in genetic mapping studies for *Yr26*^31,57^. CYR34, in contrast, was the first identified race virulent to *Yr26* (designated V26), and was responsible for the breakdown of *Yr26*-mediated resistance in approximately 2011^58^. The pathogenicity information of the two races is provided in Supplementary Data 1. Seedling responses were assessed in a greenhouse with MX169 as the susceptible control. Each line was sown in a 20 × 20 × 15 cm plastic pot and inoculated with *Pst* urediniospores at the two-leaf stage before incubation at 10 °C/100% humidity for 24 h followed by transfer to a growth chamber (16 °C Day/10 °C night, 16-h photoperiod, 20,000 lx). Infection types (IT) were recorded 15–18 days post inoculation using a 0 to 9 scale (0–6: resistant, 7–9: susceptible)^59^. Adult-plant field trials were employed in screening M_2_ populations following EMS treatment of seed. Thirty seeds of each mutagenized line were planted in a 1.5 m row with 25 cm row spacing. Two rows of highly susceptible cultivar Xiaoyan 22 were planted after every 20 rows. MX169 was planted adjacent to the plot area and inoculated with CYR32 suspended in liquid paraffin (1:300) during late March to provide inoculum and ensure uniform disease development in the test materials. Segregating lines were selected as potential mutants. Australian *Pst* race 134 E16 A + J + T+ was used in screening EMS-treated Avocet S + *Yr24* for mutants.

The *Pt* race THTT and *Bgt* isolate E09 were used for phenotyping transgene line T_3_-17-5 (Fielder+*Yr26*) and wild type accession Fielder. Leaf rust response scoring followed McIntosh et al.^60^ at 14 days post inoculation: 1 = small uredia with necrosis; 2 = small to medium sized uredia with necrosis or chlorosis; 3 = medium sized lesions with or without chlorosis); and; = hypersensitive flecks. Wheat seedlings for evaluation of powdery mildew response at the two-leaf stage were inoculated by dusting with fungal conidiospores^61^. ITs were assessed at 10 days post inoculation using a 0 to 4 IT scale, where ITs 0, 0;, 1, and 2 indicated resistance, and ITs 3 and 4, corresponded to a susceptible reaction^62^.

### Molecular markers development

KASP and PCR markers based on SNPs identified in RNA-seq or re-seq data of 92R137 and YM158 were developed to narrow down the interval of *Yr26*. KASP primers were designed with Polymarker (http://www.polymarker.info/) using sequences flanking SNPs. PCR marker primers were designed with Primer Premier 6. The primers were synthesized by Sangon Biotech Co., Ltd. (Shanghai). The PCR composition and procedure for KASP markers were set up according to the instructions^63^. The PCR procedure was initiated with a 2 min denaturation step at 98 °C, followed by a touchdown phase of 10 cycles at 98 °C for 10 s, 65 °C for 15 s with a 1 °C decrease in temperature per cycle and 72 °C for 90 s, and followed by 25 cycles at 98 °C for 10 s, 55 °C for 15 s and 72 °C for 90 s, and completion at 72 °C for 5 min and transfer to 6 °C. The reaction mixture for PCR contained 200 ng DNA, 12.5 μL 2×Taq Master Mix (Vazyme, Nanjing), 10 μmol forward primer, 10 μmol reverse primer and ddH_2_O added to obtain 25 μL. PCR markers used in fine mapping were genotyped by Sanger sequencing. Linkage of *YR26* and polymorphism of markers were assessed on a secondary F_2_ population consisting of 8 homozygous resistant, 8 homozygous susceptible and 16 segregating lines based on markers *WRS-435* and *WRS-312*^31^. A diagnostic PCR marker *DM2* was developed to distinguish wheat lines containing the *Yr26* and *yr26* alleles. A gene-based KASP marker for *Yr26* was also designed based on SNP4. Primer information for these markers is provided in Supplementary Data 2.

### Genetic map construction

Linkage analysis was performed using JoinMap v4.0 (http://www.kyazma.nl) and visualized with Mapchart v2.3^64^. Genetic distance was estimated from recombination values using the Kosambi function^65^.

### EMS mutagenesis

Seeds of 92R137 and NIL-R were mutagenized with 0.7% EMS (Solarbio, Beijing). Seeds were incubated for 18 h in water, then incubated for 14 h with shaking at room temperature in EMS solution. Na_2_S_2_O_3_ solution (pH = 7.2) was added to neutralize the EMS. After washing for 24 h under running tap water, seeds were propagated in the field. A single spike from each M_1_ plant was harvested and M_2_ plants were infected in the field with *Pst* race CYR32. Eight thousand seeds of 92R137 and five thousand seeds of NIL-R were mutagenized, respectively. Twenty-one and four candidate susceptible mutant lines were selected from 92R137 and NIL-R. The Wheat660K array genotype of M_2_ plants and susceptible M_3_ progeny of segregating M_2_ families confirmed six independent susceptible mutant lines; five (Mut2-6) derived from 92R137 and one (Mut1) from NIL-R. Generation and screening of mutants for *Yr24* using Avocet S + *Yr24* seed and EMS was based on the protocol described by Mago et al*.*^66^.

### MutChromSeq and MutRNASeq analysis to isolate *Yr24*

Flow sorting and sequencing of chromosome 1B (Chr1B) from Avocet S + *Yr24* and mutant lines M15, M22 and M316 were performed following the protocol described by Sharma et al*.*^5^. Purity of the sorted chromosome fractions ranged from 30 to 57%. Avocet S + *Yr24* chromosome 1B was sequenced in-depth (200× coverage, output 170 Gb) and each of the three mutants were sequenced to 60× coverage (output 52 Gb). Total RNA from *Pst*-infected leaves of Avocet S + *Yr24* and mutants M15, M22, M54, M65 and M316 were extracted using a Maxwell^®^ RSC Plant RNA Kit (Promega, USA) following the manufacturer’s protocol. RNA samples were sequenced by Novogene (Singapore), generating more than 20 million paired-end reads per sample.

Raw DNA and RNA reads were assessed for quality using FastQC v0.11.9^67^ and trimmed using Trimmomatic v0.39^68^ to remove adapter sequences and low-quality regions. Clean DNA reads from the MutChromSeq wild-type line were de novo assembled using MaSuRCA v4.0.5^69^ with default parameters. The resulting assembly was soft-masked for repetitive elements using RepeatMasker v4.1.2 (http://www.repeatmasker.org/) with the *Triticeae* repeat library.

Mutant MutChromSeq and MutRNASeq reads were aligned to the soft-masked WT Chr1B assembly using BWA-MEM v0.7.17^70^. SAMtools v1.15 was used for sorting and indexing of all alignments. Variant calling for both MutChromSeq and MutRNASeq datasets was performed using GATK HaplotypeCaller v4.1.9.0^71^, retaining high-confidence SNPs (QUAL > 30, DP > 5).

### Wheat transformation

A 5802-bp genomic fragment (2099-bp promoter, 2945-bp CDS and 758-bp terminator) from BAC clone B634-10C was cloned into the pCambia3301 vector with the ubiquitin promotor removed. *Agrobacterium*-mediated genetic transformation was carried out in CYR32 susceptible wheat cv. Fielder. The presence of the target gene in T_0_ or T_1_ generation transgenic plants was confirmed by PCR amplification using specific primers listed in Supplementary Data 12.

CRISPR-Cas9 genome editing was employed to knock out *G4*. Two sgRNAs of *G4* were designed using the CRISPR-P 2.0 tool (http://crispr.hzau.edu.cn/CRISPR2/). The sgRNAs were cloned into a pENTR:*sgRNA4* vector and then transferred into vector Cas9-PCL4 by Gateway LR recombination. The recombinant constructs were transformed into line 92R137 via *Agrobacterium*-mediated transformation. Putative transgenic plants were initially screened by PCR amplification targeting the *Cas9* gene, sgRNA cassette, and Bar selection marker. The sgRNA frank-specific primers were then used to amplify the target region, followed by Hi-TOM sequencing to selected *g4* mutant plants. The primer sets are listed in Supplementary Data 12.

### Gene expression analysis

Total RNA was isolated from inoculated fresh leaves using a Plants Quick RNA Isolation Kit (Huayueyang Biotechnology, Beijing). Complementary DNA was synthesized from 1 μg total RNA using a HiFiScript gDNA Removal cDNA Synthesis Kit (Cwbio, Beijing). Real-time qRT-PCR was performed on a CFX Connect Real-Time instrument (Bio-Rad, Hercules, USA) with ChamQ SYBR qPCR Master Mix (Vazyme). The resulting reverse transcription product was diluted 1-fold and used for qRT-PCR. The 20 μL reaction mixture contained 10 μL 2× qPCR Master Mix, 0.4 μL each of 10 μM forward and reverse primers, 3 μL diluted cDNA template, and 6.2 μL nuclease-free water. PCR amplification cycling conditions consisted of initial denaturation at 95 °C for 30 s, followed by 40 cycles of 95 °C for 10 s and 60 °C for 30 s. Wheat genome elongation factor DNA (*TaEF1α*) was used as an endogenous control. Primers for qRT-PCR are listed in Supplementary Data 12.

### Virus-induced gene silencing mediated by barley stripe mosaic virus

We used siRNA-Finder (siFi21) software^72^ to analyze the coding sequences of *G4* to create specific fragments for BSMV-VIGS. Two fragments (1as and 2as) for *G4* were designed and obtained by PCR and subsequently cloned into the barley stripe mosaic virus vector resulting in BSMV:γ1/2as. For VIGS assays, three experimental groups were established: BSMV:γ1/2as (*G4*-silenced plants), BSMV:γ00 (empty vector control), and BSMV:γTaPDS (phytoene desaturase-silenced positive control). Viral inoculum was prepared by mixing equal volumes of in vitro transcribed γ (or γ1/2as, γTaPDS), α, and β RNAs in 1× FES buffer (500 mM glycine, 300 mM K_2_HPO_4_, 1% sodium pyrophosphate, 1% bentonite, 1% celite, pH 8.5) and inoculated onto second leaves of two-leaf-stage seedlings. Mock controls received 1× FES buffer without viral RNA. Primers for VIGS vectors are listed in Supplementary Data 12.

After 24 h incubation in darkness at 100% humidity, plants were transferred to a growth chamber (25 °C, 16/8 h light/darkness cycle). Systemic silencing was confirmed when BSMV:γTaPDS plants exhibited photo bleaching (~12 days post-inoculation). Subsequently, fourth leaves were inoculated with the *Pst* race CYR32 isolate. Silencing efficiency was quantified on inoculated leaves collected at 0 and 24 h by real-time qRT-PCR. Stripe rust responses were recorded at 14–16 days post inoculation (dpi). Three independent biological replicates were performed for all experiments.

### BAC library screening, sequencing, assembling and annotation

In our previous study, a bacterial artificial chromosome (BAC) library was constructed from genomic DNA of 92R137 digested with restriction enzymes *Hind*III and *Bam*HI^73^. The library comprised 765,696 clones (390,144 from *Hind*III digestion, 375,552 from *Bam*HI digestion). Average insert sizes were estimated at 129 and 113 kb, respectively. Primers for screening the BAC library were developed based on the IWGSC RefSeq v.1.0 sequence in the *Yr26* candidate region (Supplementary Data 12). The PCR mixture contained 1 μL BAC solution, 7.5 μL 2× Taq Master Mix (Vazyme), 5 mol each primer and ddH_2_O added to obtain 15 μL. The amplification program was 94 °C for 4 min, 35 cycles each consisting of 94 °C for 30 s, 60 °C for 1 min, 72 °C for 1 min, and extension at 72 °C for 10 min. Amplicons were resolved on 1.2% agarose gels, and positive clones were confirmed through two rounds of re-amplification. The plasmid DNA from the positive BAC clones were extracted using a modified alkaline lysis protocol^73^. The plasmid DNA from positive BAC clones was extracted using a modified alkaline lysis protocol. Briefly, cells were resuspended in 50 mM glucose, 25 mM Tris-HCl (pH 8.0), and 10 mM EDTA, lysed with 0.2 M NaOH and 1% SDS, neutralized with 3 M potassium acetate (pH 5.5), and precipitated with isopropanol. The DNA pellet was washed with 70% ethanol and treated with RNase A. The purified DNA was then sheared into 8–10 kb fragments using mechanical methods. SMRTbell libraries were constructed from the fragmented DNA as specified by the manufacturer. Sequencing was performed in standard mode to generate continuous long reads (CLRs). Finally, the reads were assembled into contigs with canu^74^ under default parameters.

### DNA and RNA sequencing

Genome resequencing and RNA-seq were performed on the DNBSEQ platform at BGI (Shenzhen). Genomic DNA was extracted from seedling leaves using a Plant Genomic DNA Kit (Tiangen, Beijing). Each sample of approximately 10 mg of DNA was used to construct 350-bp paired-end libraries with Bioruptor Pico Sonication System (diagenode). Each library was sequenced to generate 150-bp paired-end reads by whole genome resequencing producing ~1.1 × 10^9^ 100-bp paired-end reads (depth of ~11×). Total RNA from the *Pst*-infected leaves at 24 h and 48 h post inoculation were extracted using the Maxwell RSC Plant RNA Kit (Promega, AS1500). The mRNA was enriched using oligo (dT) beads and then used to prepare strand-specific cDNA libraries. The cDNA libraries were sequenced to generate 150-bp paired-end reads.

### Prediction and annotation of the *Yr26* coding sequence

The coding sequence of *Yr26* was predicted using the NCBI open reading frame finder tool (https://www.ncbi.nlm.nih.gov/orffinder/). Conserved domains were annotated through the Conserved Domain Database (https://www.ncbi.nlm.nih.gov/Structure/cdd/cdd.shtml), InterPro (https://www.ebi.ac.uk/interpro/) databases. TMHMM 2.0 (https://services.healthtech.dtu.dk/services/TMHMM-2.0/)^75^ and Protter (http://wlab.ethz.ch/protter/#)^76^ were used to predict transmembrane topology. The three-dimensional structure of the YR26 was predicted by AlphaFold v3.0 (https://alphafoldserver.com/)^77^. lDDT score was used to determine the model confidence where lDDT > 0.6 is considered a reasonable model, and lDDT > 0.8 is considered highly acceptable, accuracy-wise. The highest IDDT score of YR26 was 0.57. The predicted protein structure and positions of non-redundant amino acid substitutions were visualized using PyMOL v2.3.0.

### Iso-Seq of wheat line 92R137 after *Pst* inoculation

Total RNA was isolated from leaves of 92R137 seedlings inoculated with *Pst* race CYR32 at 24, 48 and 168 hpi using a DP411 RNAprep Pure polysaccharide polyphenol plant total RNA extraction kit (TIANGEN). About 300 ng of total RNA was used as input for cDNA synthesis using a SMARTer PCR cDNA Synthesis Kit (TAKARA, USA). Iso-Seq library construction was performed using a Primer, Polymerase and PacBio Binding kit (PacBio, USA), followed by purification with AMpure PB Beads (PacBio). Sequencing was conducted on a SMRT Cell 8 M (Pacbio) using the PacBio Sequel II system at Haorui Genomics (Xi’an).

### Homologous gene and micro-collinearity analyses

To investigate the evolutionary origin of *Yr26*, we performed BlastN and BlastP searches against NCBI non-redundant protein sequence database and and eight additional databases, collectively covering over 36,000 genomes across plants, animals, fungi, protists, and bacteria. For plants, we searched WheatOmics (44 hexaploid, 4 tetraploid, and 26 diploid wheat, and 10 other *Triticeae* accessions; http://wheatomics.sdau.edu.cn/blast/blast.html) and Ensembl Plants (70 dicots, 74 monocots and 79 other plant genomes; https://plants.ensembl.org/Multi/Tools/Blast). For animals and other eukaryotes, we used Ensembl (97 *Homo sapiens* and 21 other primates, 77 rodents and lagomorphs, 137 other mammals, 101 birds, 20 reptiles, 7 amphibians, 301 fish, 138 coleoptera, 316 Diptera, 33 Hemiptera, 190 Hymenoptera, 783 Lepidoptera, 37 other insect species, 47 other arthropods, 197 other animals, and 22 other eukaryotes genomes; https://www.ensembl.org/Multi/Tools/Blast), Ensembl Metazoa (357 Metazoa; http://metazoa.ensembl.org/Multi/Tools/Blast), and Ensembl protists (237 protists genomes; http://protists.ensembl.org/Multi/Tools/Blast). For microbes, we searched Ensembl Fungi (1502 fungal genomes; https://fungi.ensembl.org/Multi/Tools/Blast) and Ensembl Bacteria (31,332 non-redundant bacterial and archaeal genomes; http://bacteria.ensembl.org/Multi/Tools/Blast). BLAST searches were performed using the following parameters: expectation value threshold of 0.05, word size of 16, maximum target sequences of 100, and match/mismatch scores set to the default linear gap costs. Low-complexity regions were filtered, and masking was applied for the lookup table only. The parameter for automatically adjusting parameters for short input sequences was enabled, and the maximum matches in a query range was set to 0 (no limit). All other parameters were kept at default values. Micro-collinearity analysis was carried out using Triticeae-Gene Tribe (http://wheat.cau.edu.cn/TGT/)^78^.

### Multiple sequence alignment and phylogenetic tree

The homologous DNA sequences of *Yr26* were retrieved from 47 de novo assembled wheat and related species genomes using the GetSequence function on WheatOmics^79^. The genomic coordinates of *G4* homologs were determined by BlastN using the full-length *Yr26* DNA sequence as query. Sequences containing both a start codon (ATG) and a stop codon (TGA) were classified as complete genes. Multiple sequence alignment of these homologs was performed with Muscle^80^.

Public re-sequencing data from 2483 wheat accessions (2,141 hexaploid and 342 tetraploid) representing global genetic diversity were obtained from the NCBI and the National Genomics Data Center (NGDC; https://ngdc.cncb.ac.cn/). Accession details are provided in Supplementary Data 10. SNP calling was performed by mapping WGS reads to the Chinese Spring reference genome (RefSeq v.2.1) using BWA-MEM, followed by variant detection with GATK HaplotypeCaller and hard filtering of SNPs with criteria: QD > 2.0, FS < 200.0, SOR < 10.0, MQ > 40.0, FS < 60.0, SOR < 3.0, MQRankSum > −12.5, and ReadPosRankSum > −8.0^4^. The SNP variations within the *YR26* region were used to construct a phylogenetic tree based on the neighbor-joining method using MEGA v11.0.13^81^ and visualized using iTOL (https://itol.embl.de/). Accessions clustering on the same branch were assigned to a lineage, defined as a group sharing evolutionarily related haplotypes. All analyses were carried out with default parameters.

### Cell death assays in wheat protoplasts and *N*. *benthamiana*

Protoplasts were isolated from second leaves of wheat cultivar Fielder as described above. The full length CDS of *Yr26* and target fragments were cloned into the maize ubiquitin promoter-driven vector p18T (Solarbio, Beijing). Each construct (10 μg) was mixed with the luciferase (LUC) reporter plasmid (10 μg) and then transformed into protoplasts using the method reported by Saur et al*.*^82^. After incubation at 26 °C in darkness for 16–24 h, the transformed protoplasts were collected by centrifugation, suspended in 100 µL 1× Cell Culture Lysis Buffer (YEASEN, Shanghai, Catalog No. 11401-A), incubation on ice for 10 min, followed by centrifuging at 15,000 × *g* for 10 min at 4 °C. Subsequently, 100 μL of supernatant was mixed with an equal volume of Luciferase Assay Substrate (YEASEN, Catalog No. 11401-B). LUC activity of the samples was immediately detected using a GloMax®96 Microplate Luminometer (Promega). The empty vector p18T mixed with LUC reporter vector was used as a negative control. p18T-*Sr50* and p18T-*AvrSr50* mixed with LUC reporter vector were used as a positive control. Each experiment was conducted in three biological replicates.

The full length CDS of *Yr26* and target fragments were cloned into the pGR106 vector (Solarbio, Beijing) to generate recombinant plasmids, which were transformed into *Agrobacterium* strain GV3101 (Solarbio, Beijing). Bacterial cultures were grown in LB medium at 28 °C for 2–3 days, pelleted (1700 × g, 10 min), and resuspended in MMA buffer (10 mM MES, 10 mM MgCl₂, 100 µM acetosyringone, pH = 6.5). Suspensions (OD₆₀₀ = 0.6) were incubated at room temperature for 2 h before infiltration into leaves of 4-week-old *N. benthamiana* plants. Cell death was assessed 120 h post-infiltration using trypan blue staining. BAX and the empty vector were used as positive and negative controls, respectively. For inhibition of cell death, leaves of *N. benthamiana* were infiltrated with 2 mM LaCl₃ dissolved in distilled water at 8 h post-agroinfiltration^39^. All constructs were verified by Sanger sequencing (primers in Supplementary Data 12).

### Histological observations

To assess the accumulation of H₂O₂ as a cellular response to *Pst* infection, a diaminobenzidine (DAB) staining assay was performed^83^. Infected wheat leaves were excised and treated with a 1.5 mg/mL DAB solution (3,3’-diaminobenzidine; Sigma-Aldrich, USA). The samples were then incubated under light at room temperature for 6 h to allow the peroxidase-mediated polymerization of DAB, which forms a reddish-brown precipitate at sites of H₂O₂ accumulation. More than 30 infection sites were observed for each treatment.

To visualize fungal hyphae and infection structures within *Pst*-infected wheat leaves, samples were first fixed and decolorized in a mixture of acetic acid and ethanol (1:1) for 3 days. Following decolorization, the leaf samples were stained with wheat germ agglutinin (WGA) conjugated to Alexa-488 (Invitrogen, Carlsbad, CA, USA). WGA binds specifically to chitin and other glycoconjugates present in fungal cell walls. The stained samples were observed under blue-light excitation (excitation wavelength: 450–480 nm; emission wavelength: 515 nm) using a confocal microscope (Olympus BX-53). More than 30 infection sites were observed for each treatment.

### Quantification of *Pst* biomass by qRT-PCR

To investigate the biomass dynamics of the pathogen post-inoculation, leaf samples were collected at 1, 2, 3, 4, 5, 6, and 7 dpi for genomic DNA extraction, and subsequent use for fungal biomass quantification. Relative biomass of *Pst* DNA was determined by quantitative real-time PCR (qRT-PCR), using the comparative 2^-ΔΔCt^ method^84^. Specifically, the cycle threshold (Ct) values of the *PstEF1* gene (*P. striiformis* elongation factor 1, GenBank accession no. KNE93481) were normalized against those of the wheat reference gene *TaEF1α* (*T. aestivum* elongation factor 1 alpha, GenBank accession no. Q03033), which served as the internal control for both host and pathogen. All experiments were performed with three independent biological replicates, and each replicate was technically repeated in triplicate to ensure statistical robustness. Statistical significance between groups was assessed using Student’s *t*-tests. Primers for *Pst* biomass are listed in Supplementary Data 12.

### Subcellular colocalization

The target genes or fragments were inserted into the vector pYJ-GFP-Nos to generate C-terminal GFP fusion proteins. *Agrobacterium* strain GV3101 carrying constructs expressing the GFP fused protein and the plasma membrane (PM) marker TaWPI6-mCherry were co-infiltrated into *N*. *benthamiana* leaves^85^. Fluorescence signals were observed at 36 h post co-infiltration under a confocal laser scanning microscope (FV3000; Olympus, Tokyo). Each treatment included at least three biological replicates.

### Western blotting analysis

Using the same method as above, *Agrobacterium* strain GV3101 carrying GFP fusion constructs of Yr26 and its truncated fragments or mutants was infiltrated into *N*. *benthamiana* leaves. At 36 h post infiltration, the proteins were extracted from 0.1 g of fresh leaves using a Membrane and Cytosol Protein Extraction Kit (Beyotime, Shanghai, Catalog No. P0033). Lysates were centrifuged at 14,000 × *g* for 30 min (4 °C). Supernatants were collected and incubated with 6×Protein Loading Buffer (TransGen Biotech, Beijing, Catalog No. DL101-02) at 95 °C for 10 min. Denatured protein samples were resolved by 12.5% (w/v) sodium dodecyl sulfate-polyacrylamide gel electrophoresis (SDS-PAGE) gel and transferred to a PVDF membrane. Immunoblots were detected with an anti-GFP antibody. Anti-GFP Mouse Monoclonal Antibody (TransGen Biotech, Beijing, Catalog No. HT801-01) was used for the identification of GFP and GFP fusion proteins at a dilution of 1:2000. Following that, the secondary antibody used to detect the primary antibody was HRP Conjugate Goat Anti-Mouse IgG (H + L) (TransGen Biotech, Beijing, Catalog No. HS201-01) at a dilution of 1:2000.

### Measurement of Ca^2+^ influx in wheat mesophyll tissue using NMT

The Ca^2+^ influx was detected using Non-invasive Micro-test Technology (NMT Physiolyzer, Xuyue Company, China) in Zhongguancun NMT Industrial Alliance (http://nmtia.org.cn). Wheat transformation line T_3_-17-5 (carrying *Yr26*) and the wild-type accession Fielder were used in this assay. Plant growth conditions were consistent with those described above. At the second-leaf stage, second leaves were inoculated with urediniospores of *Pst* race CYR32 suspended in electrolytic fluorinating solution. Seedlings treated with an equal volume of electrolytic fluorinating solution without urediniospores served as negative controls. Each treatment included at least three biological replicates. Infected leaves at 1 and 5 days post-inoculation were selected, and mesophyll tissues were exposed by removing the abaxial epidermis and then immersed in test solution (0.5 mM CaCl_2_, 0.2 mM MES, pH = 5.8) for 3 h to measure Ca^2+^ influx in the mesophyll. Each sample was recorded for 5 min under illumination consistent with the cultivation light intensity throughout the experiment.

### Reporting summary

Further information on research design is available in the Nature Portfolio Reporting Summary linked to this article.

## Supplementary information

Supplementary Information

Peer Review file

Description of Additional Supplementary Files

Supplementary Data 1

Supplementary Data 2

Supplementary Data 3

Supplementary Data 4

Supplementary Data 5

Supplementary Data 6

Supplementary Data 7

Supplementary Data 8

Supplementary Data 9

Supplementary Data 10

Supplementary Data 11

Supplementary Data 12

Reporting Summary

## Source data

Source data

## Data Availability

The raw whole-genome re-sequencing data are available at NCBI under BioProject PRJNA1255391. The raw RNA-sequencing and isoform-sequencing data are available at NCBI under BioProject PRJNA1276679. The cloned *Yr26* gene is available in the NCBI GenBank under accession PX307891. Source data are provided with this paper.
