# Supplementary material for: A transmembrane protein confers the Yr26/Yr24/YrCH42/YrG22-mediated stripe rust resistance in wheat
Source: Nat Commun. 2026 Aug 24;17:10389. doi: 10.1038/s41467-026-76091-5 (PMC13627085; doi:10.1038/s41467-026-76091-5)
Supplement: Supplementary file 1 [file 41467_2026_76091_MOESM16_ESM.pdf]

nature portfolio

Corresponding author(s): Zhensheng Kang, Sambasivam Periyannan, Dejun Han, Qingdong Zeng

Last updated by author(s): Jul 7, 2026

# Reporting Summary

Nature Portfolio wishes to improve the reproducibility of the work that we publish. This form provides structure for consistency and transparency in reporting. For further information on Nature Portfolio policies, see our Editorial Policies and the Editorial Policy Checklist.

## Statistics

For all statistical analyses, confirm that the following items are present in the figure legend, table legend, main text, or Methods section.

| n/a | Confirmed | |
|---|---|---|
| ☐ | ☒ | The exact sample size (*n*) for each experimental group/condition, given as a discrete number and unit of measurement |
| ☐ | ☒ | A statement on whether measurements were taken from distinct samples or whether the same sample was measured repeatedly |
| ☐ | ☒ | The statistical test(s) used AND whether they are one- or two-sided<br>*Only common tests should be described solely by name; describe more complex techniques in the Methods section.* |
| ☒ | ☐ | A description of all covariates tested |
| ☒ | ☐ | A description of any assumptions or corrections, such as tests of normality and adjustment for multiple comparisons |
| ☐ | ☒ | A full description of the statistical parameters including central tendency (e.g. means) or other basic estimates (e.g. regression coefficient) AND variation (e.g. standard deviation) or associated estimates of uncertainty (e.g. confidence intervals) |
| ☐ | ☒ | For null hypothesis testing, the test statistic (e.g. *F*, *t*, *r*) with confidence intervals, effect sizes, degrees of freedom and *P* value noted<br>*Give P values as exact values whenever suitable.* |
| ☒ | ☐ | For Bayesian analysis, information on the choice of priors and Markov chain Monte Carlo settings |
| ☒ | ☐ | For hierarchical and complex designs, identification of the appropriate level for tests and full reporting of outcomes |
| ☒ | ☐ | Estimates of effect sizes (e.g. Cohen's *d*, Pearson's *r*), indicating how they were calculated |

*Our web collection on statistics for biologists contains articles on many of the points above.*

## Software and code

Policy information about availability of computer code

| | |
|---|---|
| Data collection | Phenotypic data (disease severity, cell death scores, histological observations, $Ca^{2+}$ influx) were collected manually without dedicated software. For genome resequencing and RNA-seq, library preparation and sequencing were performed at BGI (Shenzhen) on the DNBSEQ platform. Raw sequencing data were generated using the platform's standard control software. No custom code was used during data collection. |
| Data analysis | 1. Statistical analysis<br>Two-group comparisons were performed using the two-tailed Student's t-test function from the rstatix package (version 0.7.3). For comparisons among three or more groups, one-way ANOVA was conducted via the aov function from the base R stats package, followed by post-hoc multiple comparisons using Tukey's HSD test. Significance levels were indicated as follows: *P < 0.05, **P < 0.01, ***P < 0.001.<br><br>2. BAC clones sequencing, assembling and annotation<br>The plasmid DNA of the positive BAC clones were extracted and sheared into 8-10 kb fragments using mechanical methods. SMRTbell libraries were constructed from the fragmented DNA. Sequencing was performed in standard mode to generate continuous long reads (CLRs). Finally, the reads were assembled into contigs with canu under default parameters.<br><br>3. Gene analysis<br>Prediction and annotation of the Yr26 coding sequence: the coding sequence of Yr26 was predicted using the NCBI open reading frame finder tool (https://www.ncbi.nlm.nih.gov/orffinder/). Conserved domains were annotated through the Conserved Domain Database (https://www.ncbi.nlm.nih.gov/Structure/cdd/cdd.shtml), InterPro (https://www.ebi.ac.uk/interpro/) databases. TMHMM 2.0 (https://services.healthtech.dtu.dk/services/TMHMM-2.0/) and Protter (http://wlab.ethz.ch/protter/#) were used to predict transmembrane topology. |

The three-dimensional structure of the YR26 was predicted by AlphaFold v3.0 (https://alphafoldserver.com/). The predicted protein structure and positions of non-redundant amino acid substitutions were visualized using PyMOL v2.3.0.
Homologous genes and micro-collinearity analysis: BlastN and BlastP searches were performed to against NCBI non-redundant protein sequence database and the WheatOmics and Ensembl databases. Micro-collinearity analysis was carried out using Triticeae-Gene Tribe (http://wheat.cau.edu.cn/TGT/).

4. Multiple sequence alignment and phylogenetic tree
Multiple sequence alignment of the homologous DNA sequences of Yr26 was performed with Muscle. A phylogenetic tree of the YR26 region were constructed based on the neighbor-joining method using MEGA v11.0.1364 and visualized using iTOL (https://itol.embl.de/).

5. Genetic map construction
Linkage analysis was performed using JoinMap v4.0 and visualized with Mapchart v2.3. Genetic distance was estimated from recombination values using the Kosambi function.

6. MutChromSeq and MutRNASeq analysis to isolate Yr24
Raw DNA and RNA reads were assessed for quality using FastQC v0.11.9 and trimmed using Trimmomatic v0.39 to remove adapter sequences and low-quality regions. Clean DNA reads from the MutChromSeq wild-type line were de novo assembled using MaSuRCA v4.0.5 with default parameters. The resulting assembly was soft-masked for repetitive elements using RepeatMasker v4.1.2 (Smit et al., http://www.repeatmasker.org/) with the Triticeae repeat library. Mutant MutChromSeq and MutRNASeq reads were aligned to the soft-masked WT Chr1B assembly using BWA-MEM v0.7.17. SAMtools v1.15 was used for sorting and indexing of all alignments. Variant calling for both MutChromSeq and MutRNASeq datasets was performed using GATK HaplotypeCaller v4.1.9.0, retaining high-confidence SNPs (QUAL >30, DP >5).

For manuscripts utilizing custom algorithms or software that are central to the research but not yet described in published literature, software must be made available to editors and reviewers. We strongly encourage code deposition in a community repository (e.g. GitHub). See the Nature Portfolio guidelines for submitting code & software for further information.

## Data

Policy information about availability of data

All manuscripts must include a data availability statement. This statement should provide the following information, where applicable:
- Accession codes, unique identifiers, or web links for publicly available datasets
- A description of any restrictions on data availability
- For clinical datasets or third party data, please ensure that the statement adheres to our policy

The raw whole-genome re-sequencing data were deposited in NCBI under BioProject PRJNA1255391 (https://www.ncbi.nlm.nih.gov/bioproject/PRJNA1255391), and the raw RNA-sequencing and isoform-sequencing data were deposited under BioProject PRJNA1276679 (https://www.ncbi.nlm.nih.gov/bioproject/PRJNA1276679/). The cloned Yr26 gene is available in the NCBI GenBank under accession number PX307891 (https://www.ncbi.nlm.nih.gov/nuccore/PX307891). Source Data are provided with this paper.

## Research involving human participants, their data, or biological material

Policy information about studies with human participants or human data. See also policy information about sex, gender (identity/presentation), and sexual orientation and race, ethnicity and racism.

| | |
|---|---|
| Reporting on sex and gender | Not applicable |
| Reporting on race, ethnicity, or other socially relevant groupings | Not applicable |
| Population characteristics | Not applicable |
| Recruitment | Not applicable |
| Ethics oversight | Not applicable |

Note that full information on the approval of the study protocol must also be provided in the manuscript.

# Field-specific reporting

Please select the one below that is the best fit for your research. If you are not sure, read the appropriate sections before making your selection.

☒ Life sciences ☐ Behavioural & social sciences ☐ Ecological, evolutionary & environmental sciences

For a reference copy of the document with all sections, see nature.com/documents/nr-reporting-summary-flat.pdf

# Life sciences study design

All studies must disclose on these points even when the disclosure is negative.

| | |
|---|---|
| Sample size | A diversity panel to explore alleles of YR26 comprised 911 tetraploid accessions (including 392 wild emmer (Triticum turgidum L. ssp. dicoccoides), 225 cultivated emmer (T. turgidum L. ssp. dicoccum), 294 durum (T. turgidum var. durum)) and 105 synthetics or derivatives (Supplementary Data 6). For haplotype diversity analysis and marker validation of Yr26, a panel of 2,541 Chinese breeding lines/cultivars was used (Supplementary Data 8). A total of 122 individual plants derived from an F2 population (DEW072/D026) were used for the genetic analysis of Yr26_h5 to determine its disease resistance function. |
| Data exclusions | No data were exclude from the analyses. |
| Replication | Three independent biological replicates were performed for BSMV--VIGS, detection of transgenic lines, cell death assays, histological observations, and subcellular colocalization, and four independent biological replicates for $Ca^{2+}$ influx experiments. |
| Randomization | Randomization was not relevant for the diversity panel analyses because no treatment groups were defined. For all functional experiments, biological replicates were randomly allocated to different treatment conditions. |
| Blinding | Blinding is not necessary for sampling accessions for genome sequencing. The samples for genome sequencing were selected based on their geographic distribution and phylogenetic tree to cover the geographic distribution and genetic diversity of the species. The investigators were blinded to the population structure or origin of accessions when collecting trait data in the field. Blinding was implemented during phenotyping of the gene-mapping population plants, diversity panels, mutant lines, and transgenic plants, as well as in qRT-PCR assays for gene expression analysis, histological observations, and cell death assays. |

# Reporting for specific materials, systems and methods

We require information from authors about some types of materials, experimental systems and methods used in many studies. Here, indicate whether each material, system or method listed is relevant to your study. If you are not sure if a list item applies to your research, read the appropriate section before selecting a response.

## Materials & experimental systems

| n/a | Involved in the study | |
|---|---|---|
| ☐ | ☒ | Antibodies |
| ☒ | ☐ | Eukaryotic cell lines |
| ☒ | ☐ | Palaeontology and archaeology |
| ☒ | ☐ | Animals and other organisms |
| ☒ | ☐ | Clinical data |
| ☒ | ☐ | Dual use research of concern |
| ☐ | ☒ | Plants |

## Methods

| n/a | Involved in the study | |
|---|---|---|
| ☒ | ☐ | ChIP-seq |
| ☒ | ☐ | Flow cytometry |
| ☒ | ☐ | MRI-based neuroimaging |

## Antibodies

| | |
|---|---|
| Antibodies used | Anti-GFP Mouse Monoclonal Antibody (TransGen Biotech, Beijing, Catalog No. HT801) |
| Validation | This antibody has been validated by the manufacturer (TransGen Biotech) for the detection of GFP and GFP fusion proteins via Western blotting. It has been widely cited in previous publications for GFP-tagged protein detection in various biological samples. In this study, the antibody was used at a dilution of 1:2000 and successfully detected the target GFP fusion proteins with expected molecular weights, with no significant non-specific bands observed under the experimental conditions (Supplementary Fig. 10c). |

## Dual use research of concern

Policy information about dual use research of concern

### Hazards

Could the accidental, deliberate or reckless misuse of agents or technologies generated in the work, or the application of information presented in the manuscript, pose a threat to:

| No | Yes | |
|---|---|---|
| x | | Public health |
| x | | National security |
| x | | Crops and/or livestock |
| x | | Ecosystems |
| x | | Any other significant area |

## Experiments of concern

Does the work involve any of these experiments of concern:

| No | Yes | |
|---|---|---|
| x | | Demonstrate how to render a vaccine ineffective |
| x | | Confer resistance to therapeutically useful antibiotics or antiviral agents |
| x | | Enhance the virulence of a pathogen or render a nonpathogen virulent |
| x | | Increase transmissibility of a pathogen |
| x | | Alter the host range of a pathogen |
| x | | Enable evasion of diagnostic/detection modalities |
| x | | Enable the weaponization of a biological agent or toxin |
| x | | Any other potentially harmful combination of experiments and agents |

# Plants

| | |
|---|---|
| Seed stocks | The wheat accessions are available at CGRIS, NSGC, IPK genebank, ICARDA genebank, and CIMMYT Wheat Germplasm Bank. More details are listed in Supplementary Table 4. |
| Novel plant genotypes | 1)The independent transgenic lines for Yr26 were generated by agrobacterium-mediated transformation.<br>2) The EMS mutants of 92R137 and Avocet+Yr24.<br>3)The CRISPR-Cas9 genome editing system were used to knock out Yr26. |
| Authentication | EMS-induced susceptible mutants, CRISPR/Cas9-based knockout plants, and transgenic lines were utilized for comprehensive analysis. All mutants and transgenic plants were verified through RNA-seq, PCR amplification and Sanger sequencing analysis. |
