# Supplementary material for: A transmembrane protein confers the Yr26/Yr24/YrCH42/YrG22-mediated stripe rust resistance in wheat
Source: Nat Commun. 2026 Aug 24;17:10389. doi: 10.1038/s41467-026-76091-5 (PMC13627085; doi:10.1038/s41467-026-76091-5)
Supplement: Supplementary file 2 [file 41467_2026_76091_MOESM1_ESM.pdf]

A transmembrane protein confers the *Yr26*/*Yr24*/*YrCH42*/*YrG22*-mediated stripe rust resistance in wheat

Liu *et al.*

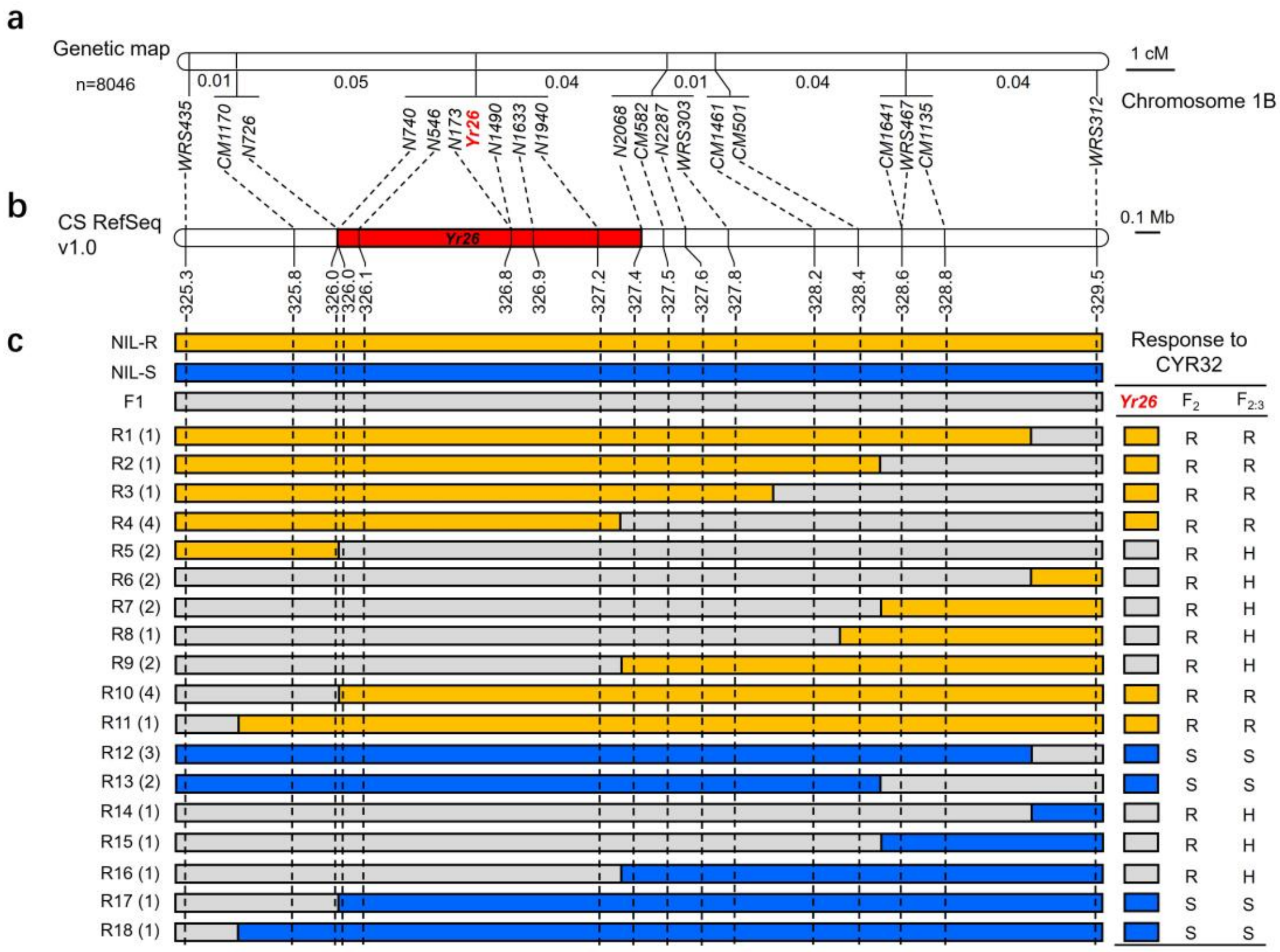

Supplementary Figure 1. Fine mapping of *Yr26*. a. Genetic map of *YR26*. Marker sources: CM, 660K array; WRS and N, RNA-seq-derived SNP. **b.** Physical map of *YR26* based on Chinese Spring IWGSC RefSeq v1.0. Red rectangle represents the *YR26* confidence interval on chromosome arm 1BL. **c.** Eighteen recombinants (R1 – 18) identified in the F_2 population used for fine mapping of *YR26*. R, S and H, homozygous resistant, homozygous susceptible, and heterozygous, respectively.

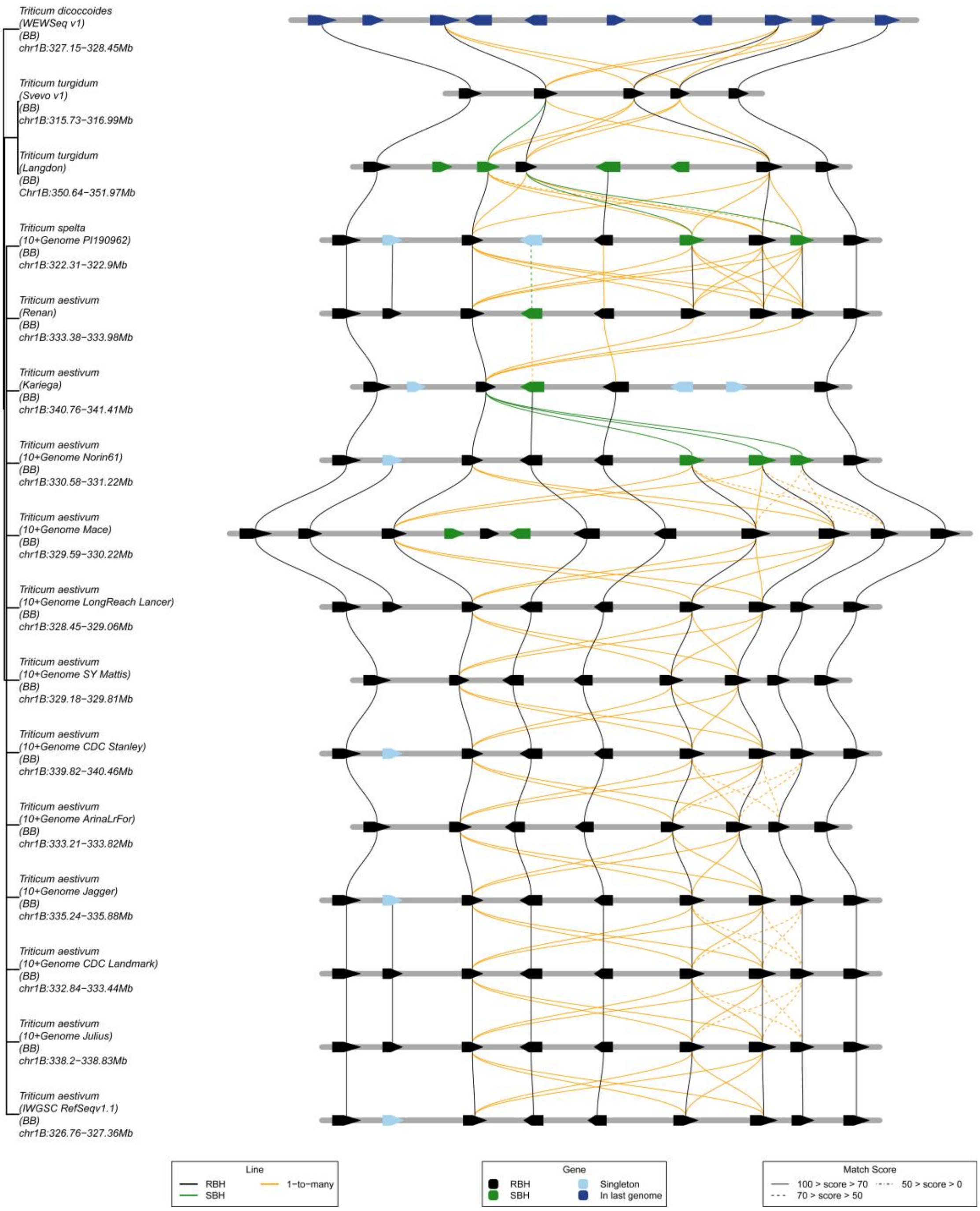

Supplementary Figure 2. Micro-collinearity of the *Yr26* fine-mapping region in different *Triticeae* genomes. RBH, reciprocal best hit; SBH, single-side best hit.

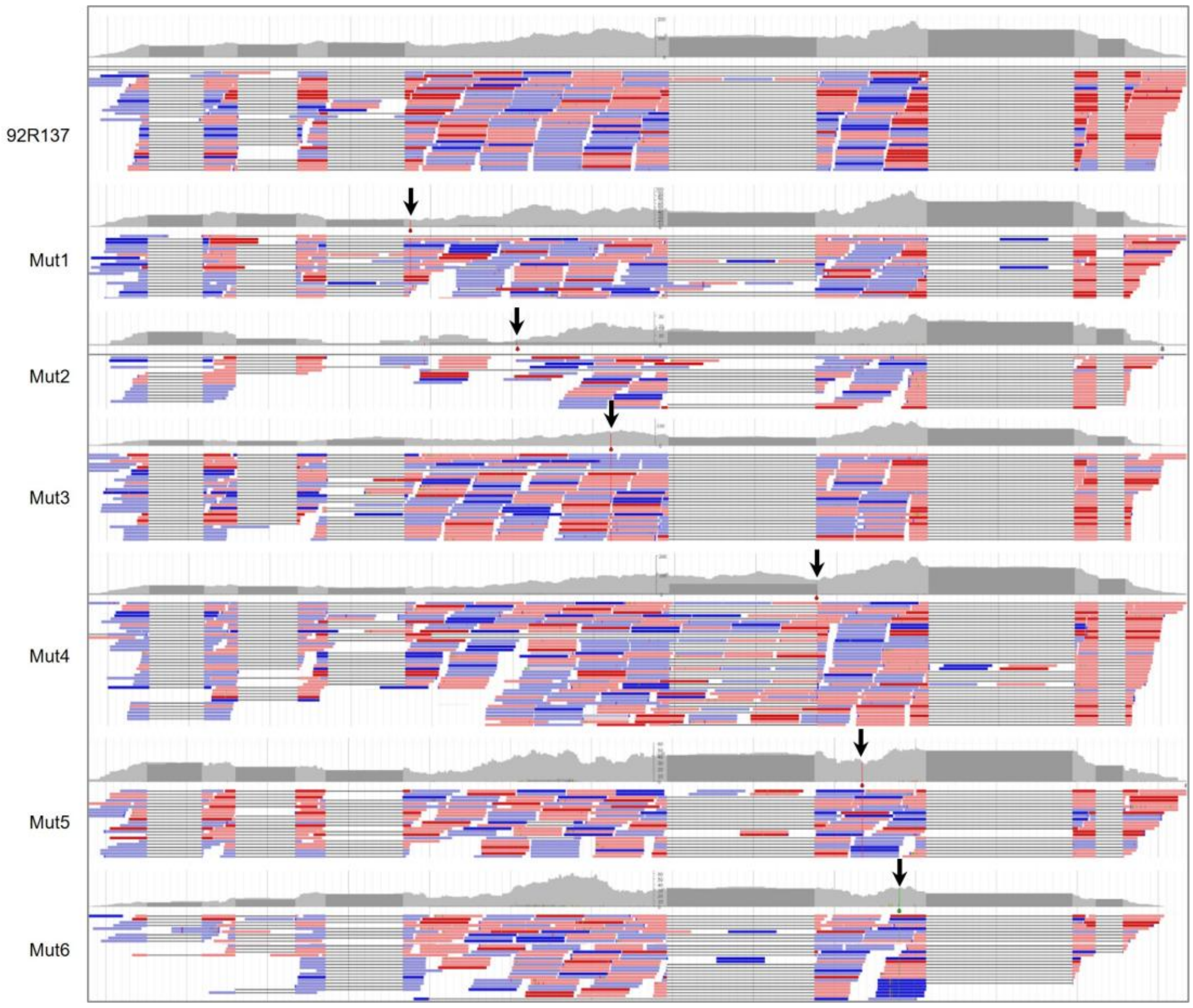

Supplementary Figure 3. MutRNA-seq analysis of *Yr26* candidate gene *G4*. Mut1-6, six susceptible mutant lines selected following EMS mutagenesis. Black arrows indicate the locations of mutations. Light and dark gray, exons and introns. The Arabic numerals on the scale lines represent the number of reads in RNA-seq data. Red and blue bars under the gene represent reads.

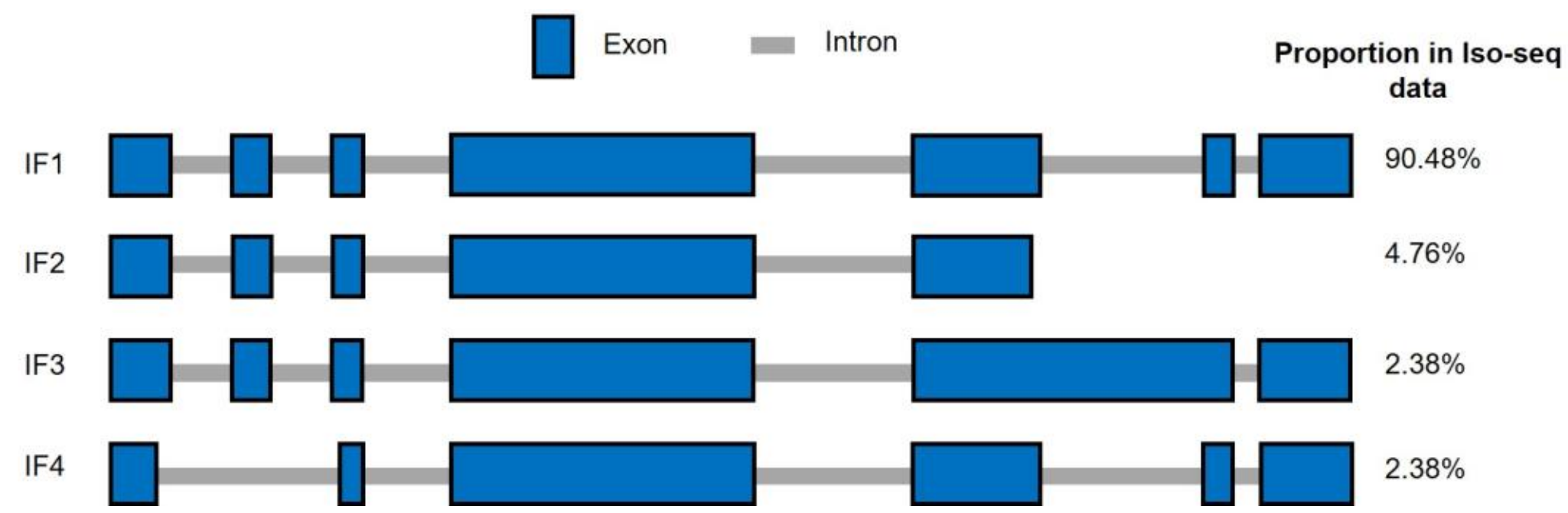

Supplementary Figure 4. Full-length transcript isoforms of *G4* (*Yr26* candidate) identified by PacBio Iso-Seq. Four major splice variants (*IF1 – IF4*) are shown. The percentage at the right represents the proportion of each isoform in the sequence data.

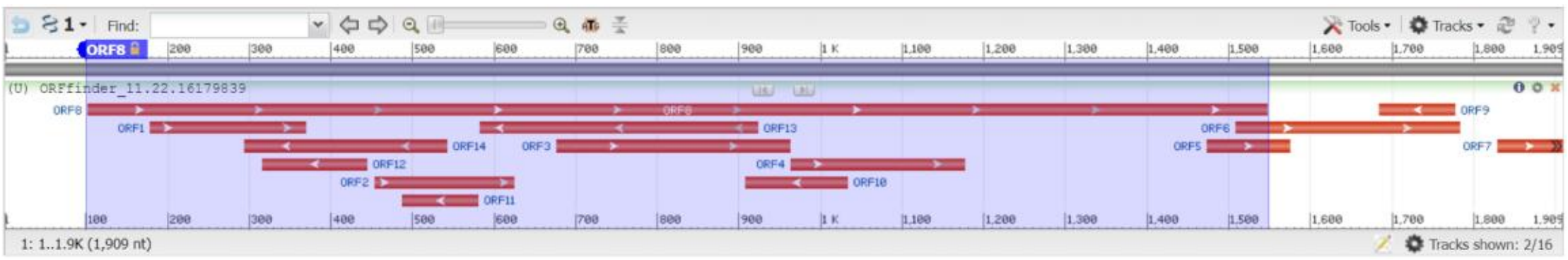

Supplementary Figure 5. Open reading frame (ORF) prediction for *G4* using ORFinder in NCBI. The longest *ORF8* with 481 amino acids was selected as the candidate functional coding sequence of *G4*.

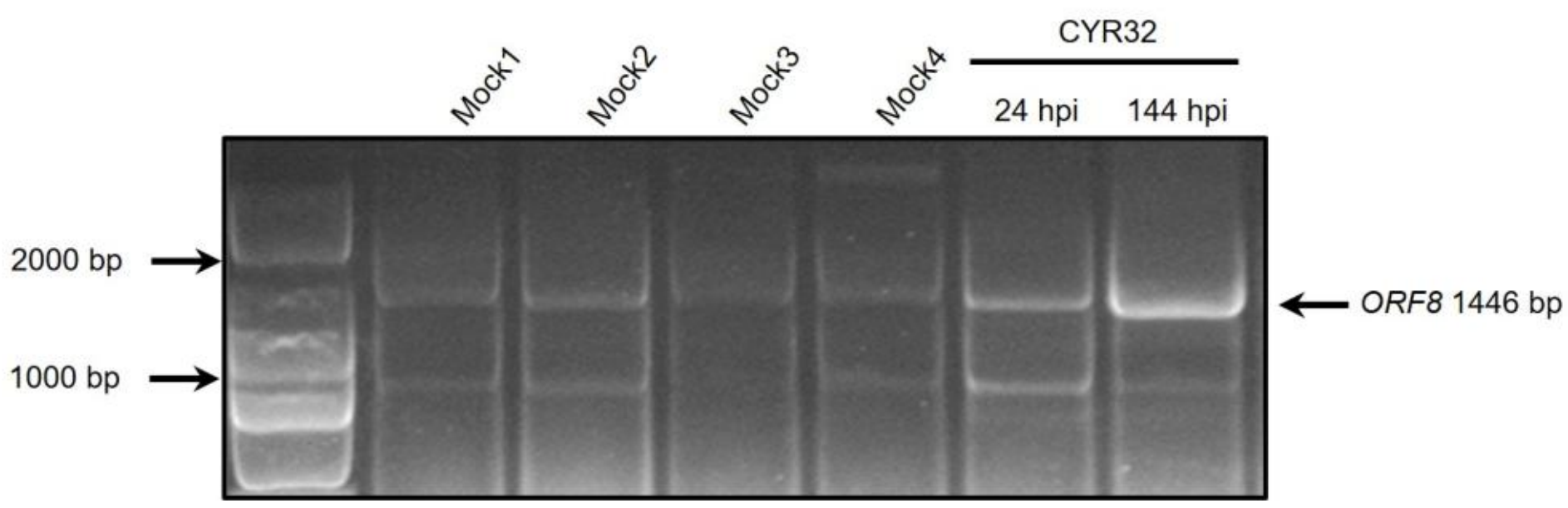

Supplementary Figure 6. Expression of *Yr26* in plants inoculated with ddH_2O and *Pst* races CYR32. The cDNA samples were collected from leaves 24 and 144 hours post inoculation (hpi). Source data are provided as a Source Data file.

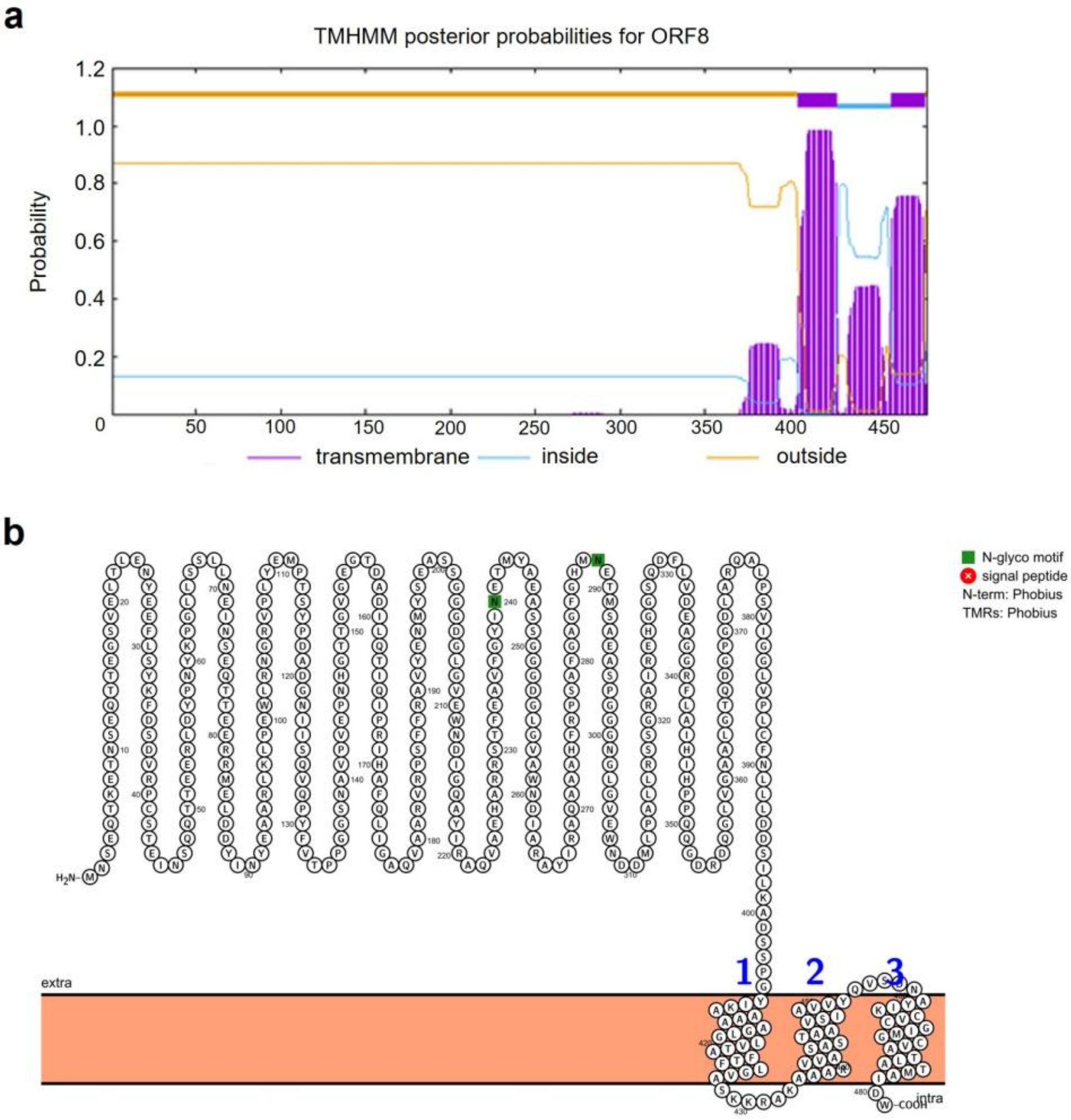

Supplementary Figure 7. Transmembrane (TM) structure predictions for Yr26. a. Two TM helices by TMHMM 2.0[1]. **b.** Two Three TM helices by Protter[2]. Default parameters were applied for both tools.

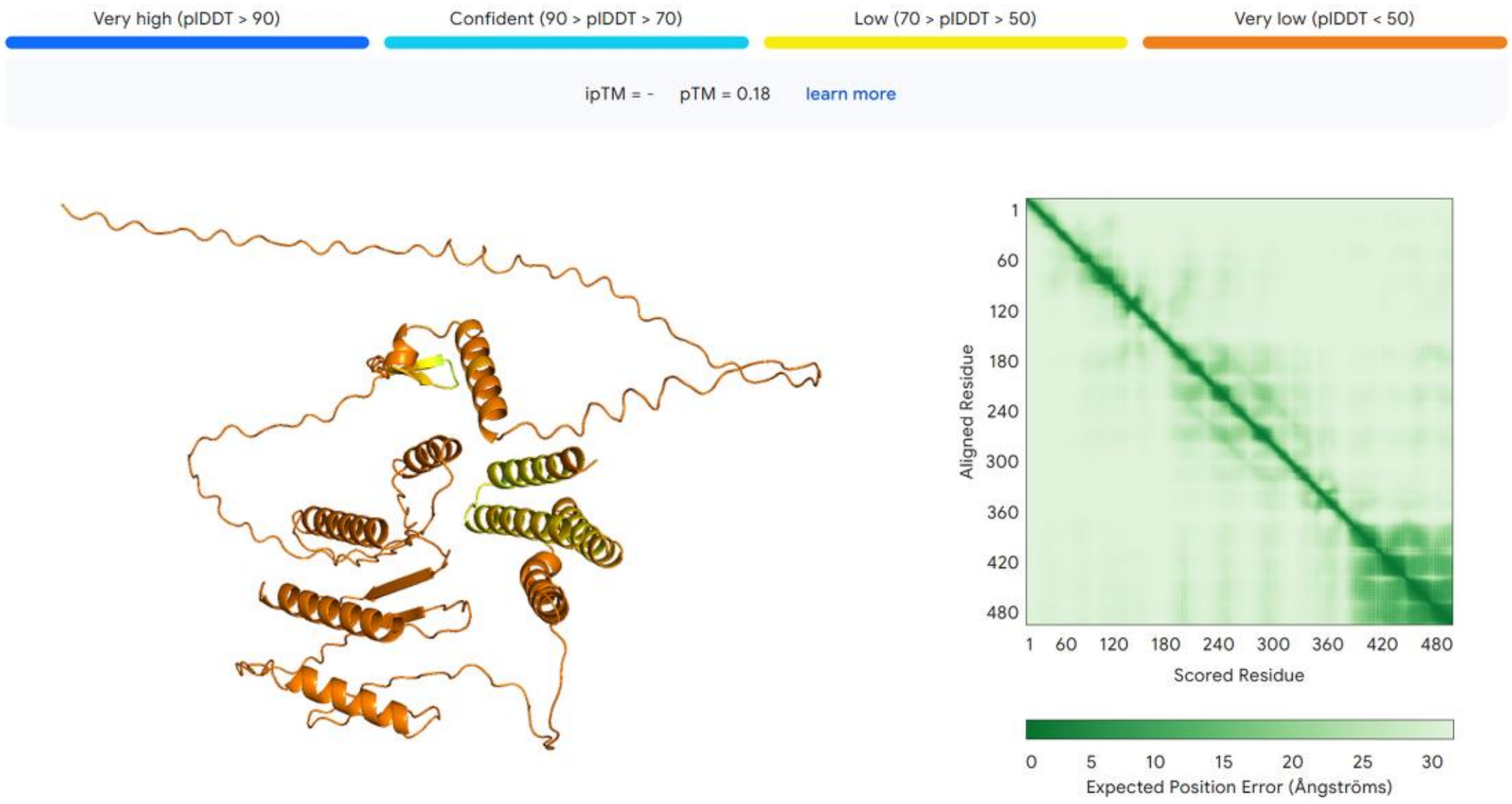

Supplementary Figure 8. Quality assessment of the protein structure predicted by AlphaFold v3[3]. The upper colored bar indicates the predicted local distance difference test score (pIDDT), which evaluates model confidence per residue: very high confidence (pIDDT > 90) in dark blue; confident (90 > pIDDT > 70) in light blue; low (70 > pIDDT > 50) in yellow; and very low (pIDDT < 50) in orange. The predicted post-translational modification (iPTM) is shown as "-", and the predicted template modeling score (pTM) is 0.18. The lower line plot shows the predicted positional error for each residue, with higher values indicating lower reliability in that region. The x-axis represents the amino acid residue position (1-481). The highest ranking score for the model is 0.57.

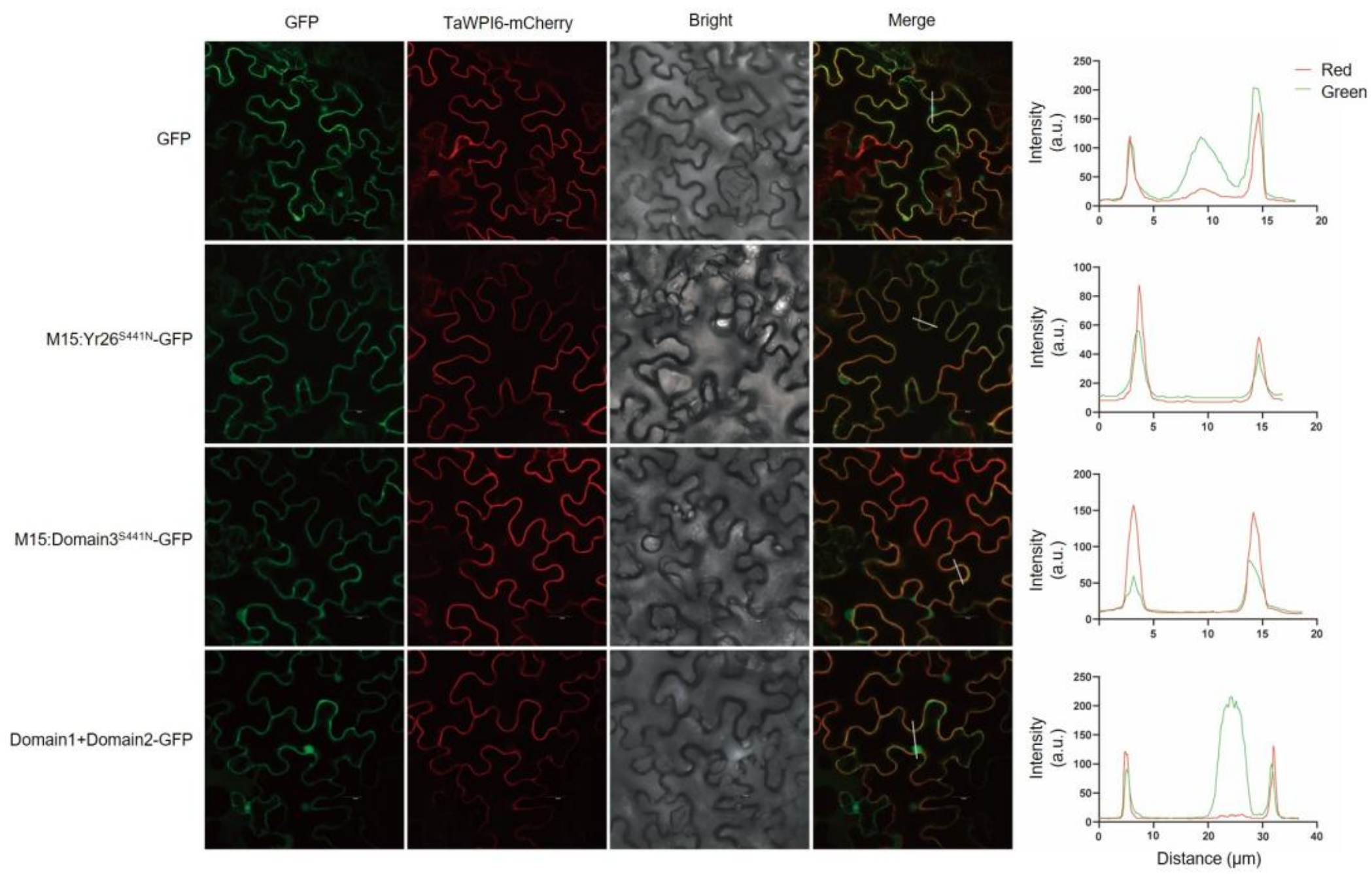

Supplementary Figure 9. Subcellar localization of Yr26 and its predicted domains in *N. benthamiana* epidermal cells. C-terminal GFP fusion proteins were generated using the pYJ-GFP-Nos vector and were transiently expressed in *N. benthamiana* leaves. TaWPI6-mCherry were co-infiltrated served as a cytoplasmic membrane localization marker. GFP (green) and mCherry (red) fluorescence signals were observed, along with bright-field (Bright) and merged (Merge) images. The fluorescence intensity profiles (right) quantify the relative fluorescence intensity (a.u.) along the white lines shown in the merge images, with distance measured in micrometers (μm). Similar results were obtained from three independent biological replicates (n = 3). Experiments were performed without randomization or blinding, and no data were excluded. a.u., atomic units. Source data are provided as a Source Data file.

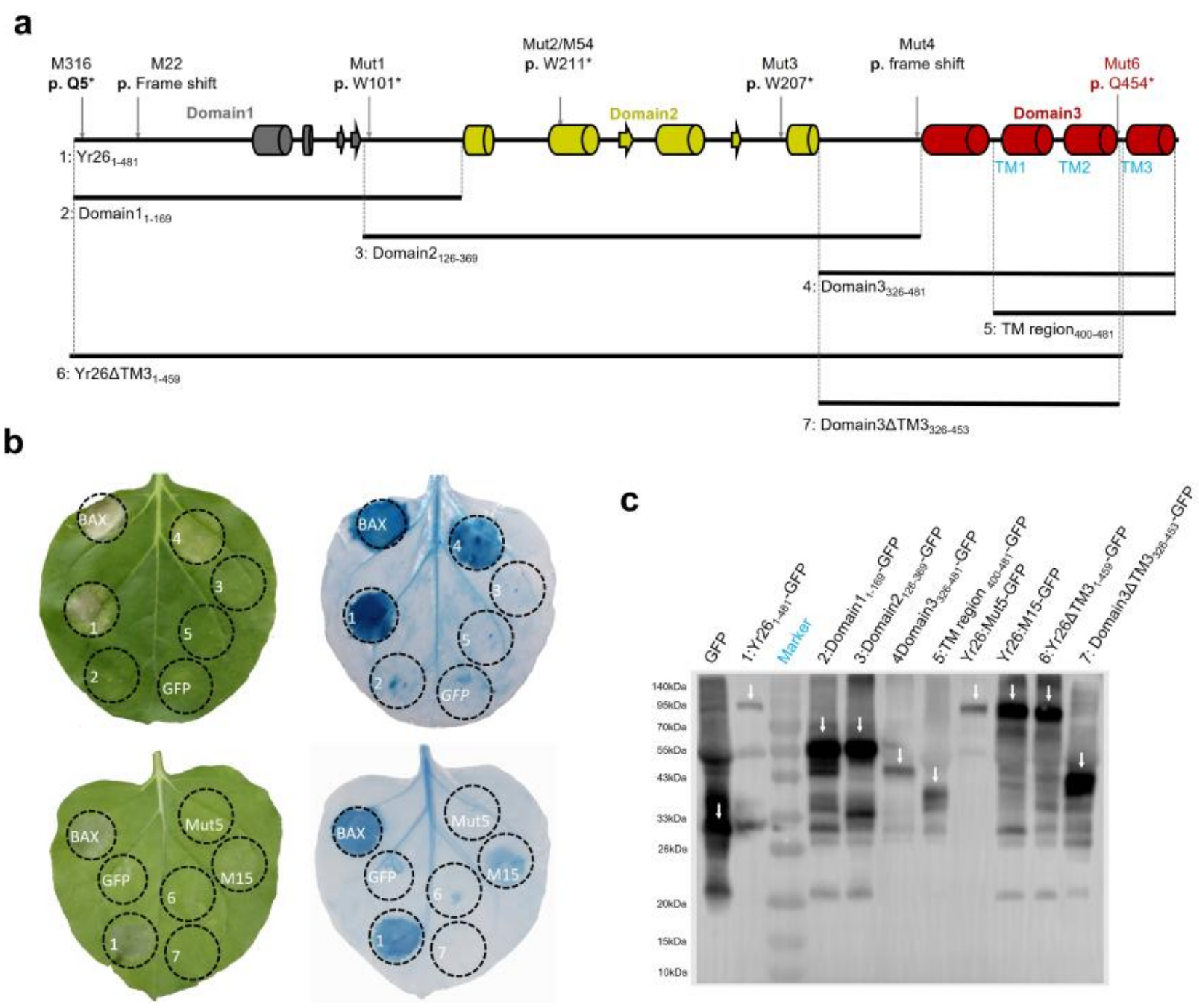

Supplementary Figure 10. *Yr26* induces cell death in *N. benthamiana*. a. Secondary structure of *Yr26*. Cylinders, α-helices; arrows, β-sheets. Protein domains are illustrated by colors described above. Arrows indicate the position of the six loss-of-function mutations generated by EMS. Black bars and "Δ" represent constructs used to transform wheat protoplasts and remove the corresponding domain, respectively. **b.** Transient expression of *Yr26* and its truncated structures (C-terminal 3×Flag-tagged) induced cell death in *N. benthamiana* under a Cub promoter at 48 h post inoculation. *Bax* (inducing cell death) and *GFP* were used as controls. The experiment was performed three times with similar results. Dead cells were identified by trypan blue staining. Similar results were obtained from three independent cell replicates (n = 3). Overexpression of full-length Yr26 or Domain 3 alone induced cell death, whereas deletion of the TM3 helix or introduction of Domain 3 point mutations (G416R, S441N) abolished or severely reduced this activity, indicating that an intact Domain 3 structure is essential for Yr26-mediated HR and pathogen containment. **c.** Protein expression analysis of *Yr26* and its truncated/mutant variants as GFP fusion proteins. Protein extracts were separated by SDS-PAGE and detected by anti-GFP antibody. GFP alone used as control. The molecular weight of marker is shown on the left. White arrows indicate the expected bands of the fusion proteins. Similar results were obtained from three independent biological replicates (n = 3). For all experiments shown in this figure, randomization or blinding was applied where applicable, and no data were excluded. Source data are provided as a Source Data file.

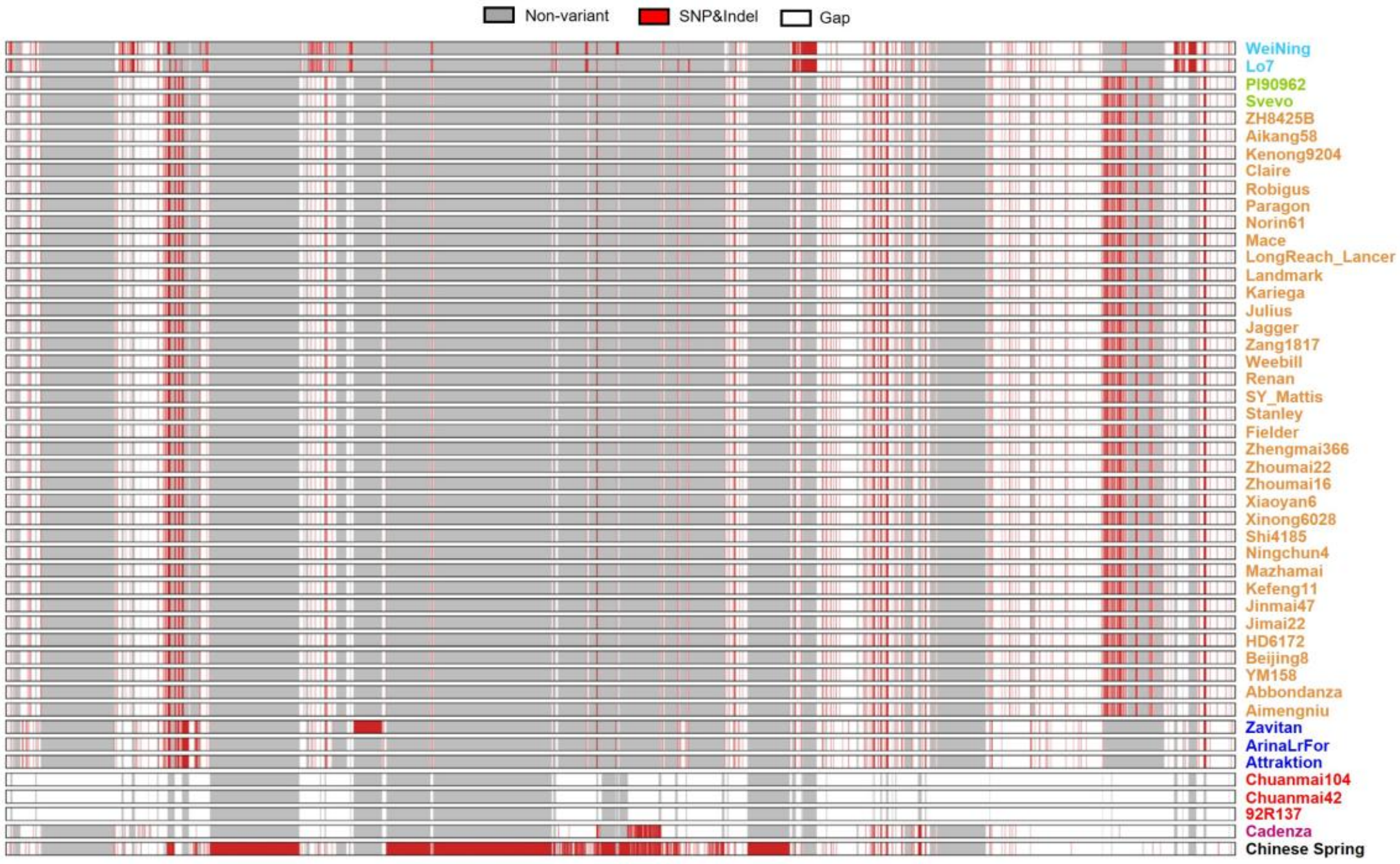

Supplementary Figure 11. Multiple sequence alignments of *Yr26* homologs across 47 assembled genomes of wheat and its relatives. Genomes with same sequence are marked in the same color at the right.

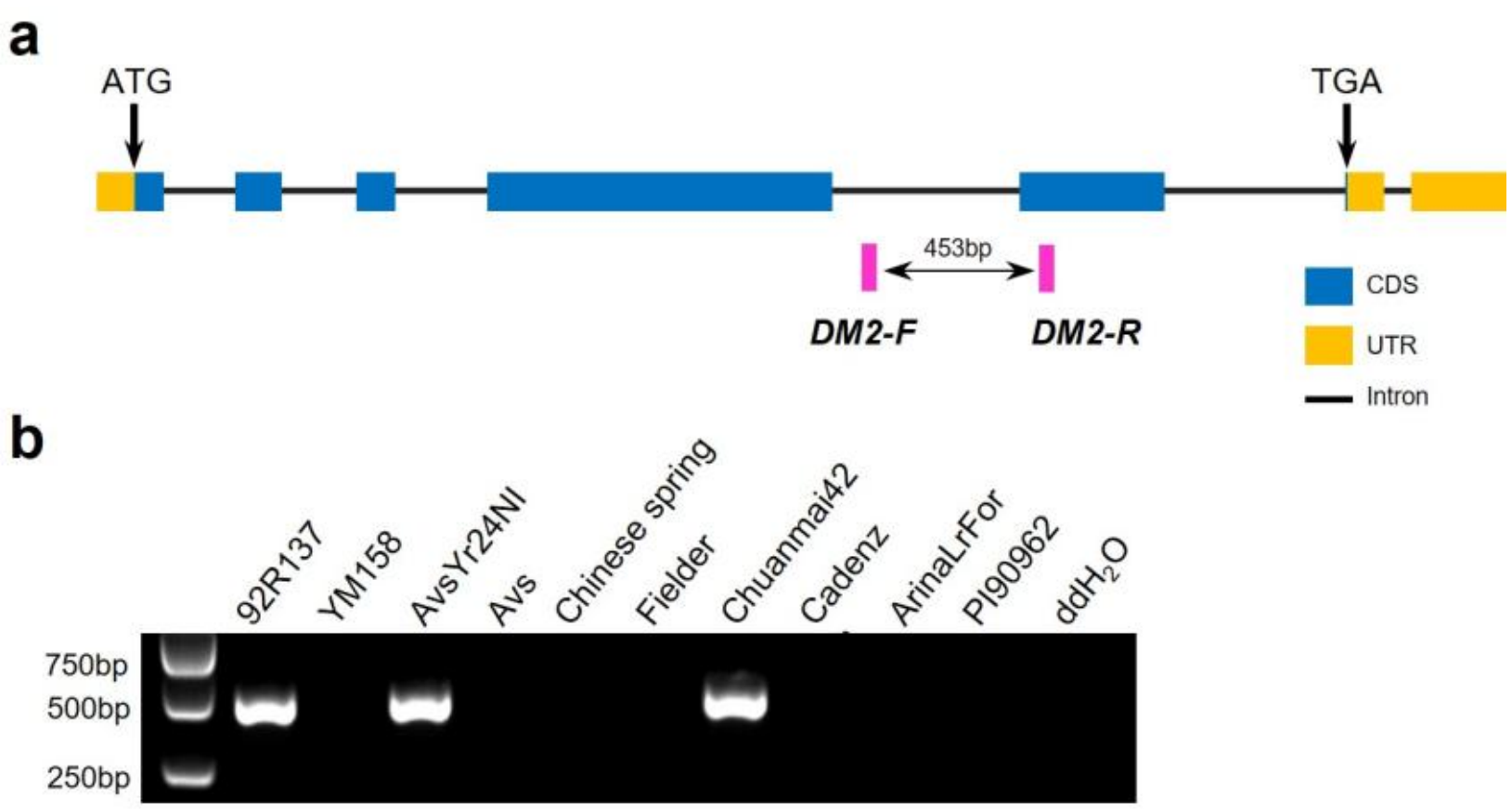

Supplementary Figure 12. *YR26* locus-specific DNA marker *DM2*. a. Schematic of the *DM2* marker in the *Yr26* allele. *DM2*-F/R, *DM2*-forward/reverse primers. **b.** Genotyping of *YR26* homologs using the *DM2* marker. A 453 bp band indicates *Yr26* allele presence. Source data are provided as a Source Data file.

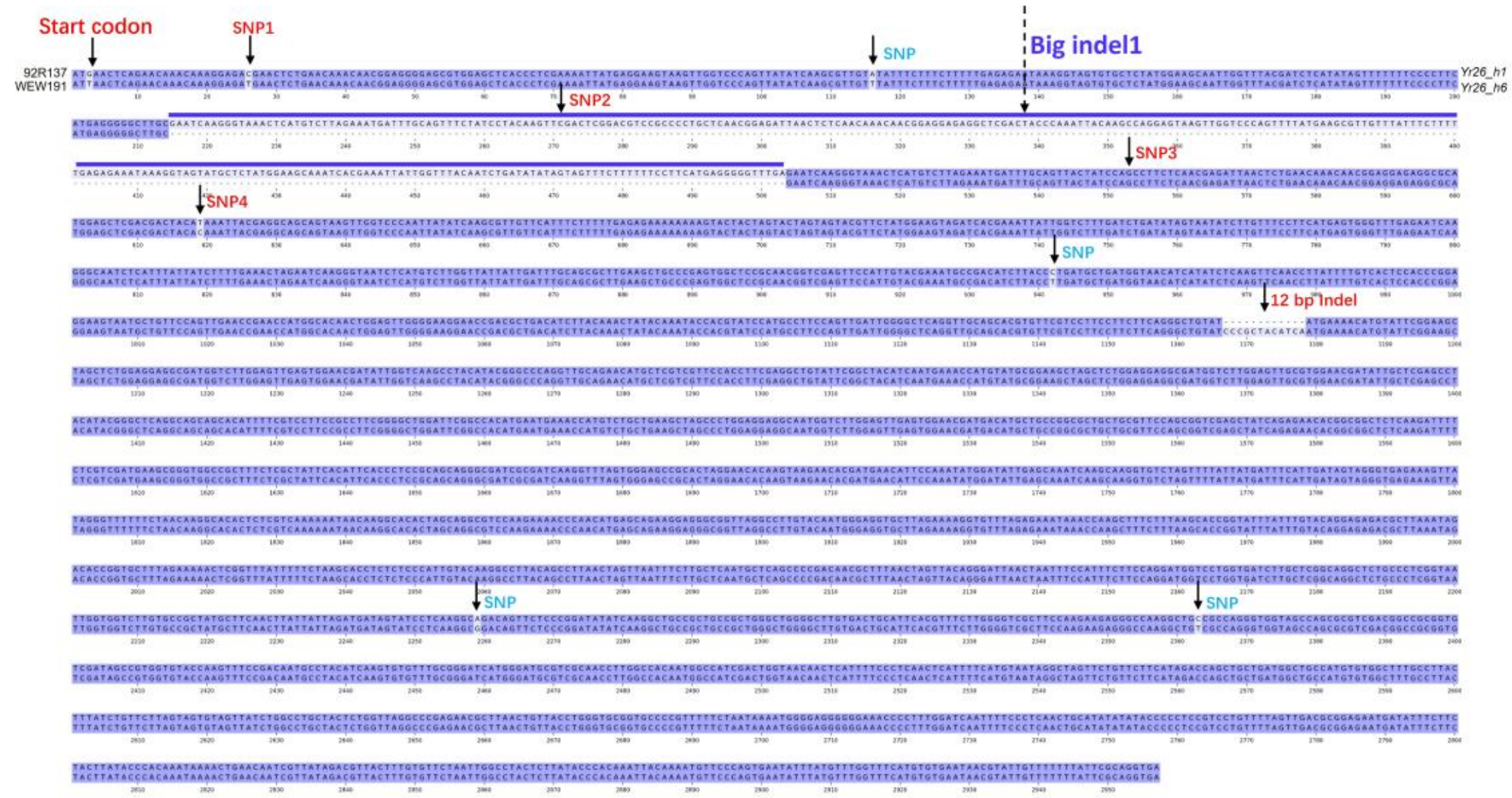

Supplementary Figure 13. Alignment of *Yr26_h1* in 92R137 and *Yr26_h6* in WEW191. Blue bar, Indel1. SNPs in haplotypes *Yr26_h1* – *_h5* are marked by black arrows with red font. In contrast, SNPs unique to the *Yr26_h1* and *Yr26_h6* comparison are indicated by black arrows with blue font.

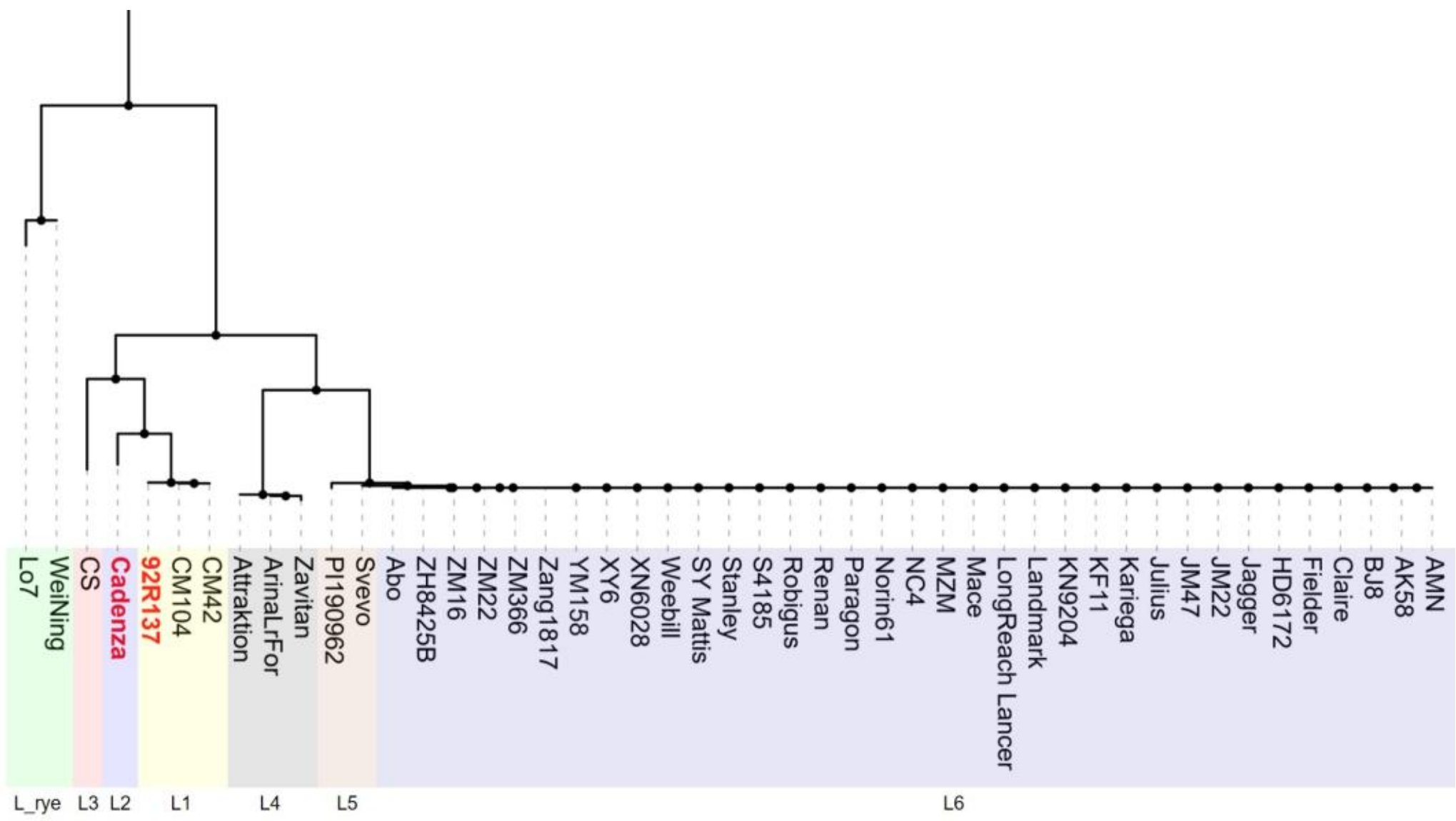

Supplementary Figure 14. Phylogenetic analysis of *YR26* homologs across 47 assembled genomes of wheat and its relatives. Alignment was conducted with Muscle. The phylogenetic tree was made with neighbor-joining procedure in MEGA v11.0.13. The tree was visualized with iTOL (https://itol.embl.de/). The accessions most closely related to *Yr26* are highlighted in red bold font.

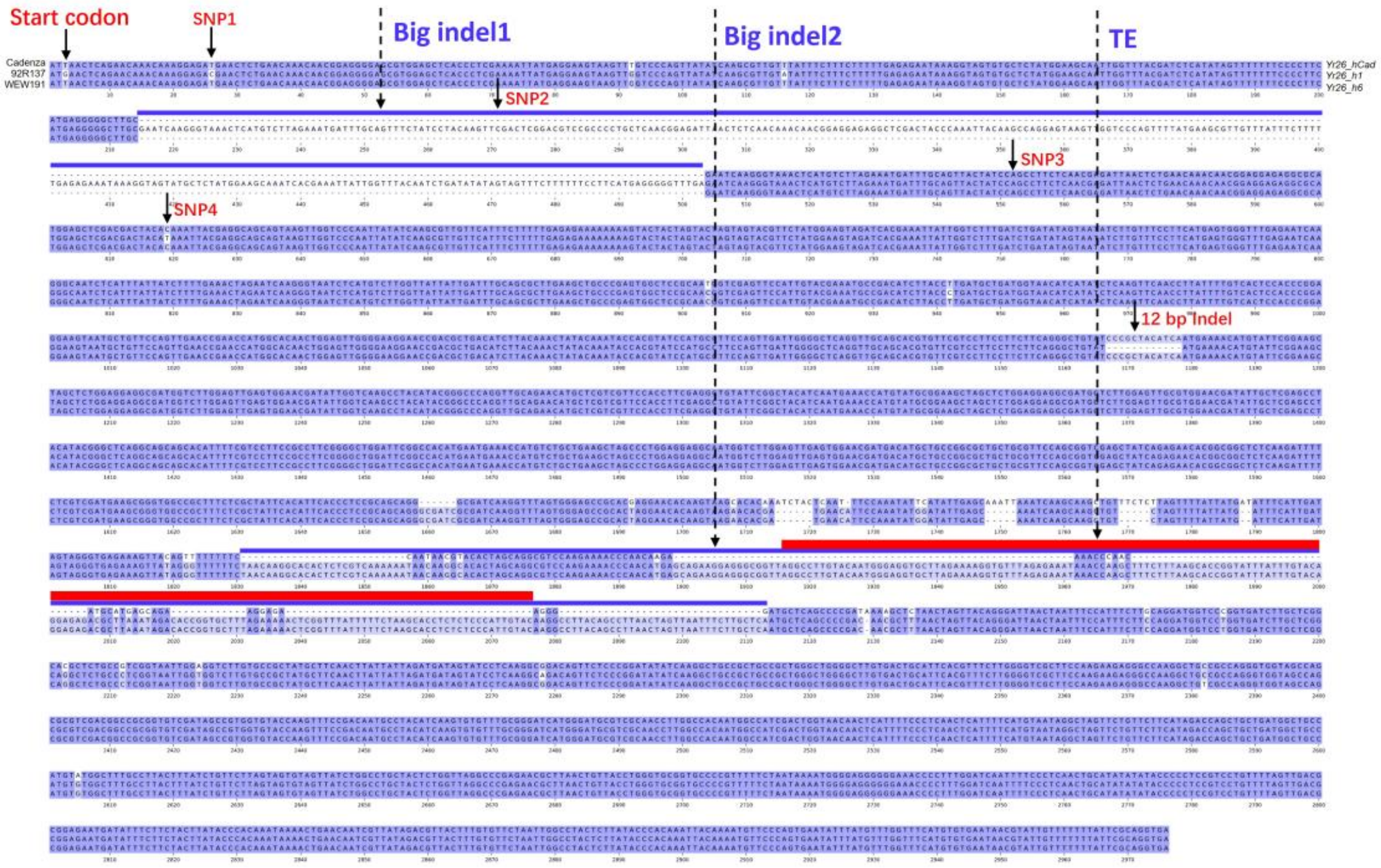

Supplementary Figure 15. Alignment of *YR26* and homolog in Cadenza. Blue bars: Indel1 and Indel2; red bar: transposon. SNP1–SNP4, indicated by black arrows, represent SNPs between *Yr26_ h1* – *h5*.

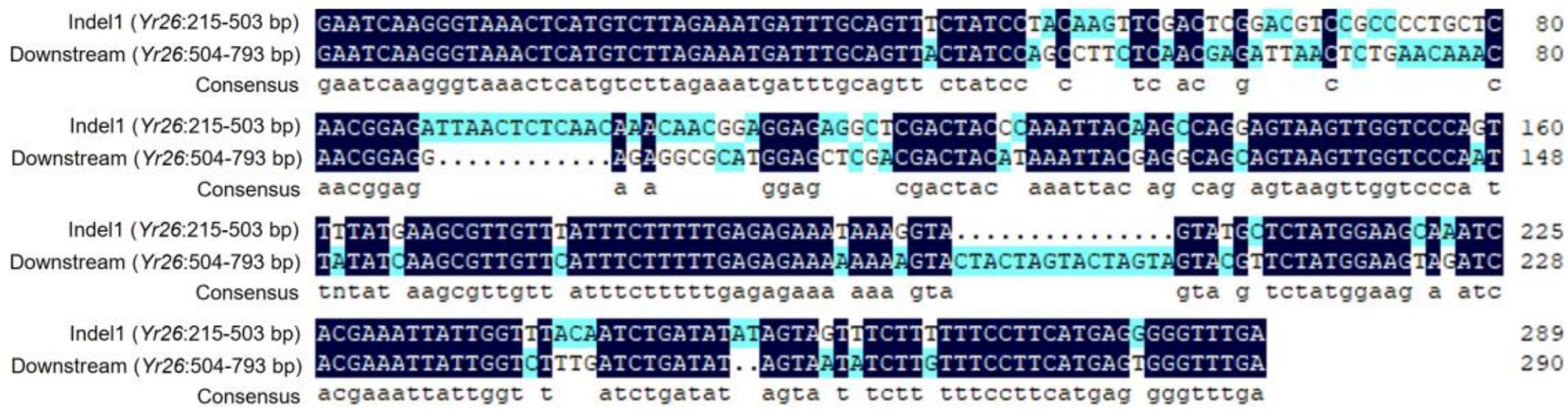

Supplementary Figure 16. Alignment of Indel1 and the downstream 289 bp segment.

Supplementary reference

1. Krogh, A. et al. Predicting transmembrane protein topology with a hidden Markov model: Application to complete genomes. *Journal of Molecular Biology* **305**, 567-580 (2001).
2. Omasits, U. et al. Protter: interactive protein feature visualization and integration with experimental proteomic data. *Bioinformatics* **30**, 884–886 (2014).
3. Abramson, J. et al. Accurate structure prediction of biomolecular interactions with AlphaFold 3. *Nature* **630**, 493–500 (2024).
