# Supplementary material for: A transmembrane protein confers the Yr26/Yr24/YrCH42/YrG22-mediated stripe rust resistance in wheat
Source: Nat Commun. 2026 Aug 24;17:10389. doi: 10.1038/s41467-026-76091-5 (PMC13627085; doi:10.1038/s41467-026-76091-5)
Supplement: Supplementary file 3 [file 41467_2026_76091_MOESM2_ESM.pdf]

nature portfolio

Peer Review File

A unique transmembrane protein confers the Yr26/Yr24/YrCH42/YrG22-mediated stripe rust resistance in wheat

Corresponding Author: Professor Qingdong Zeng

This file contains all reviewer reports in order by version, followed by all author rebuttals in order by version.

Version 0:

Reviewer comments:

Reviewer #1

(Remarks to the Author)
This manuscript presents a rigorous and impactful study on cloning the wheat stripe rust resistance gene Yr26/Yr24/YrCH42/YrG22, a widely used resistance gene in wheat breeding program, and characterizing its function, structure, and evolution. The work is methodologically sound (e.g., map-based cloning, mutant analysis, VIGS, CRISPR-Cas9, transgenic complementation) and fills critical gaps in understanding non-NLR resistance genes in wheat.
Below are comments to improve the current manuscript:
1. Abstract revision suggestion: "Yr26 is a newly evolved gene encoding a previously uncharacterized transmembrane protein, first emerged in cultivated emmer wheat and recently introgressed into bread wheat. AlphaFold v3.0 predicts a C-terminal four-helical beam domain critical for triggering cell death, a hallmark of hypersensitive resistance."
2. Yr24/Yr26/YrCH42/YrG22 were historically proposed as allelic but unconfirmed. The authors may frame the current study's validation of their identity as a major resolution to this uncertainty.
3. L177-178: The CDS of Yr24 in Avocet+Yr24 shares identical coding, sequences with Yr26 in 92R137. L243-244 stated that "An identical G4 sequence in 92R137, Avocet+Yr24, Chuanmai 42 and Guinong 22 confirmed the same stripe rust resistance gene". Are the sequences of those genes from the 4 lines including the promoter and terminator sequences? Please clarify.
4. Up to date, only Rph3 in barley and TaMlo, Lr34, Lr67, Lr14 and WAI-B2 in wheat are transmembrane resistance proteins. The authors could add 1–2 sentences on the paucity of cloned transmembrane resistance genes in the grass family to highlight Yr26's novelty in resistance mechanism.
5. Could YR26 form a pentameric cation channel as WeiTsing in Arabidopsis? The authors may perform Ca^{2+} influx assays in wheat protoplasts or Nicotiana benthamiana leaves. The Ca^{2+} blocker LaCl3 should be tested in wheat protoplasts or Nicotiana benthamiana assays.
6. The suppressed recombination in the Yr24/Yr26 genomic region should be noted. The fine genetic mapping interval of the candidate gene overlaps the centromeric region of chromosome 1B, a known recombination cold spot, to justify why further refinement beyond 0.002 cM was unfeasible.
7. The single Yr26_h5 accession (CAS075) is resistant to both avirulent (CYR32) and virulent (CYR34) races. Could the authors rule out the possibility of other gene, rather than the Yr26_h5, contribute to the resistance? A follow-up study (e.g., complementation with Yr26_h5 or VIGS of Yr26_h5) will test if it confers broad-spectrum resistance, as this is a high-impact observation.
8. Why both G4-specific fragments (1as/2as) were used in the VIGS experiment? Do both of them achieved similar knockdown efficiency (~50%)?
9. The authors speculate that Yr26 evolved in cultivated emmer. However, the authors only screeded 33 wild emmer (WEW) accessions that may be not enough evidence to exclude wild emmer as its origin.
10. "To characterize Yr26-mediated resistance seedlings of lines 92R137…" should be "To characterize Yr26-mediated resistance, seedlings of lines 92R137…"

Reviewer #2

(Remarks to the Author)

This study map-clones Yr26 using a large NIL-based F2 population (8,046 plants) and narrows the locus to a 0.002 cM interval supported by BAC contig assembly and candidate transcript annotation, ultimately prioritizing an unannotated transcript (G4) as the leading candidate. The authors provide multiple lines of functional evidence that G4 encodes Yr26, including transgene complementation in Fielder, where G4-positive lines are resistant and null/Wt lines are susceptible. They further propose that Yr26 is a previously uncharacterized transmembrane (TM) orphan gene whose C-terminal four-helix beam domain (Domain3) is required for hypersensitive response (HR)/cell death, based on AlphaFold predictions and truncation/fragment assays. Evolutionary analyses suggest Triticeae-restricted distribution and recent origin consistent with an orphan gene.

Study design, controls, and whether conclusions are supported

Overall, the study design is appropriate for the central research question—identifying the causal gene underlying Yr26-mediated stripe rust resistance and characterizing its key functional domain—because it combines:

1. high-resolution mapping with a large segregating population and physical contig support, and
2. orthogonal genetic tests (mutants, silencing/editing, and complementation; complementation evidence is explicit in the main text).

Controls are generally well chosen for the mechanistic assays: empty-vector negatives and well-established positives are used in the wheat protoplast readout (Sr50/AvrSr50) and N. benthamiana HR assays (BAX). For expression profiling, the use of avirulent vs virulent Pst races and mock controls appropriately supports the conclusion that Yr26 is pathogen-inducible during incompatible interaction.

Are conclusions supported?

• The conclusion that G4 is Yr26 is well supported by genetic mapping + complementation.
• The conclusion that Domain3 is necessary/sufficient to trigger HR-like cell death is supported by fragment overexpression phenotypes in two systems (wheat protoplasts and tobacco), with consistent negative controls.
• The manuscript's broader mechanistic speculation that Yr26 may function like a cation channel facilitating Ca^{2+} influx is explicitly framed as a hypothesis and "future research". However, in its current form, this part should remain clearly labeled as speculative unless additional functional/channel data are provided (see Major points).

Methodological soundness and reproducibility

Most core methods are described at a level that enables replication for mapping, cell death assays, and plant-pathogen phenotyping, including key parameters and controls (e.g., construct amounts and incubation for protoplasts; OD600 and buffer composition for infiltration; staining and microscopy approach). The mapping strategy and candidate interval definition are also described clearly (markers, recombinants, interval definition), though some practical details would still help reproducibility/interpretation (see below).

Statistics and uncertainties

Some sections report biological replication (e.g., "three biological replicates" for protoplast assays; ">30 infection sites" for microscopy), which is a good baseline. That said, the manuscript would benefit from a clearer, centralized statistical description: what statistical tests were used for each figure type, what "n" represents (plants, leaves, infection sites, independent experiments), whether experiments were repeated independently, and whether assumptions were met. At present, the statistical reporting appears distributed and may be insufficient for Nature Communications standards unless fully detailed in figure legends/reporting summary.

Major points (recommended revisions)

1. Mechanistic claims should be aligned with evidence (channel/Ca^{2+} hypothesis).

The discussion draws an analogy to WTS and suggests a cation-channel-like mechanism and Ca^{2+} influx. This is interesting, but the current evidence base is primarily: (i) AlphaFold-predicted TM/helix architecture and (ii) HR induction by Domain3 fragments.

Actionable fix: Either (a) add direct supporting assays (e.g., subcellular localization; oligomerization; Ca^{2+} reporter readouts after Yr26/Domain3 expression; mutational analysis of conserved residues in Domain3), or (b) tighten the text to clearly label this as a hypothesis and avoid mechanistic phrasing that implies demonstrated channel activity.

2. Clarify scope and robustness of disease-resistance phenotyping.

The paper asserts Yr26 inhibits post-infection fungal development based on microscopy differences that emerge at 5–7 dpi.

Actionable fix: Explicitly report sample sizes for phenotyping (plants/replicates per genotype/line), number of independent infection experiments, and quantitative metrics (e.g., uredinia density, fungal biomass, infection type scoring) aligned with the images. This will strengthen the causal chain from gene → HR → restricted colonization.

3. Map-based cloning section: explain "suppressed recombination" implications.

The manuscript notes suppressed recombination preventing further refinement.

Actionable fix: Provide more detail on recombination suppression evidence (e.g., local recombination rate vs flanking regions; structural variation possibility), and clarify how this limitation was overcome by BAC contigs/transcript evidence and mutant/complementation tests.

4. AlphaFold confidence and domain definitions.

The structural prediction reports lDDT max 0.57 for Domain3.

Actionable fix: Provide per-residue confidence or a more nuanced interpretation (e.g., which segments are high-confidence helices vs uncertain loops), and ensure the functional conclusions are not over-weighted on low-confidence regions. A simple supplementary figure mapping AlphaFold confidence to Domain boundaries would help.

Minor points (editorial/clarity)

1. Ensure all key controls are explicitly described in the Results text (not only Methods), especially for HR/cell death assays (negative/positive controls are stated in Methods).
2. Evolutionary/orphan-gene claims: the text states no significant homology outside Triticeae and proposes recent genomic events. Please ensure thresholds/databases and search parameters are clearly stated (e.g., BLAST e-value cutoffs, proteome sets queried) and that claims match those criteria.
3. Figure quantification: where microscopy/staining is used, pair representative images with quantified summaries and explicit "n" (infection sites counted are mentioned).

4. Reporting summary: the manuscript references a Nature Portfolio Reporting Summary. Please ensure it is complete and consistent with figure legends (replicates, randomization/blinding where applicable, data exclusions).
The genetic identification of Yr26/G4 is strong and potentially high-impact, and the domain-level functional mapping is compelling. However, for Nature Communications, the mechanistic framing around channel/Ca^{2+}-influx should either be substantiated with additional data or more carefully constrained as a hypothesis, and the resistance phenotyping/statistical reporting should be strengthened to match the journal's rigor expectations.

Reviewer #3

(Remarks to the Author)
This manuscript reports the cloning and functional validation of the wheat stripe rust resistance locus Yr26 (synonymous with Yr24/YrCH42/YrG22) and concludes that it encodes an atypical transmembrane "orphan" protein. The authors mapped the locus to a very small genetic interval, identified a single candidate transcript, and support causality using independent EMS loss-of-function mutants, BSMV-VIGS knockdown, CRISPR knockouts, and transgenic complementation into susceptible wheat. Using AlphaFold predictions, they identified several domains within the protein and propose that a C-terminal "four-helix beam" domain (Domain 3) is required for induction of cell death, based on cell-death assays in wheat protoplasts and N. benthamiana following expression of full-length Yr26 or Domain 3. Finally, they analyzed natural variation and concluded that the functional resistant haplotype (Yr26_h1) is associated with a key SNP, and that Yr26 likely first appeared in cultivated emmer and was introgressed into bread wheat through breeding.
The cloning of the gene and the realization that it corresponds to several previously mapped resistance loci is important, and the discovery that it is unrelated to known classes of R genes is novel. The authors also convincingly show that cell-death activity maps to a C-terminal domain. However, the manuscript lacks mechanistic insight, and several issues need to be addressed and clarified, particularly regarding the induction of cell death in susceptible and non-host systems and the hypothesis that the gene first appeared in domesticated emmer.

Main comments
1. Lack of sufficient mechanistic insight. The nature of resistance conferred by Yr26 remains unclear. The gene is associated with race-specific resistance, however expression of the gene in susceptible wheat as well as in tobacco leads to cell death. The authors argue that, unlike classical R genes, Yr26 does not interact with an effector, but rather operates as a protein whose activation or abundance is sufficient to trigger cell death, for example by activating downstream processes that culminate in cell death. According to this scenario, one would expect broad-spectrum resistance, potentially even resistance to multiple diseases, as reported for several non-canonical resistance genes. However, the resistance conferred by Yr26 is race-specific; The authors explain race specificity by proposing that Yr26 functions as a downstream executor of cell death whose transcription is selectively induced during incompatible interactions. If, as suggested, resistance is determined by gene activation, one would need to assume the existence of a fungal factor specific to avirulent races that selectively activates Yr26 expression. Such a factor has not been reported and would represent a novel mechanism of specificity. However, no race-specific effector, upstream receptor, or regulatory mechanism controlling Yr26 induction has been identified or proposed. Thus, while the proposed model is plausible, race specificity is inferred rather than mechanistically demonstrated.
2. Given that this is a central issue, it is essential to conduct a broader screen of pathogen races and report the resistance spectrum to additional stripe rust races, as well as to stem and leaf rusts, and possibly to other wheat pathogens.
3. Subcellular localization. Subcellular localization of Yr26 (and Domain 3) is not demonstrated in planta, which is critical for interpreting any proposed "channel-like" or membrane-associated function. The Discussion speculates about an ion-channel analogy (e.g. WTS), but this hypothesis is not experimentally tested. Such assays could be performed in N. benthamiana, since the protein induces cell death in this system.
4. Protoplast assays. The protoplast cell-death assay uses luciferase as a proxy for viability, which is reasonable. However, for a transmembrane protein it would be important to additionally show expression-level normalization (protein accumulation), determine whether cell death correlates with membrane localization, and test whether Domain 3–induced cell death is abolished by mutations that disrupt predicted transmembrane helices (structure–function analysis).
5. Evolutionary origin of Yr26. Phylogenetic analysis indicates that lineages L1/L2 (including 92R137) are absent in wild emmer but present in cultivated emmer and in descendant lineages including durum and bread wheat. Based on this, the authors conclude that the gene "first emerged in domesticated emmer and was inherited into durum". Several factors could affect the accuracy of this conclusion, including sampling bias and difficulties in presence/absence calling from short-read data, which is particularly problematic for orphan genes. More importantly, the presence of homologs in rye (which diverged from wheat more than 5 MYA) does not align with the hypothesis that the gene itself first appeared in cultivated emmer. A more appropriate interpretation of the data would be that the functional resistant allele likely arose in domesticated emmer (or in an unsampled wild lineage) and was subsequently selected and introgressed, rather than that the gene itself "appeared" at this stage. This interpretation is also more consistent with the authors' own suggestion of precursor or non-functional alleles and a multistep evolutionary model. I would also recommend more robust validation of wild emmer "absence," such as coverage-based thresholds, assembly-based confirmation in multiple WEW genomes, or targeted PCR across a broader WEW collection. Finally, if the authors want to propose that the gene originated in domesticated emmer, the mechanism underlying such gene emergence should be explicitly addressed.

Additional comments
6. Line 106: How was this isolate selected?
7. Line 117: The text does not align with the DAB results shown in Figure 1. The results at 48 hpi show strong DAB staining in the resistant interaction, suggesting an early response. However, the text states that the results indicate that "Yr26 inhibited post-infection fungal development". A time-course analysis of ROS accumulation starting at 12hpi should be conducted, and the text revised accordingly. Additionally, quantitative measurement of ROS would be more appropriate than

reliance on DAB staining alone. The authors may also consider PI staining to determine the onset of cell death.
8. Line 231: Please specify that the cultivar used for the protoplast assay was cv. Fielder, which is susceptible. It would have been informative to perform the same assay in a resistant line as well.
9. Lines 257–269: Only two races were tested - CYR32 (avirulent) and CYR34 (virulent). This is insufficient to draw general conclusions about race specificity or to determine whether CYR34 virulence reflects effector loss, suppression, or bypass. Additional races should be tested.
10. Lines 343–345: The sentence referring to “trigger cell death in intact transmembrane domains” is unclear. The data are insufficient to support the claim that these domains “are strictly upregulated during infection leading to hypersensitive responses.”
11. Lines 355–356: No evidence is provided to support the proposed mechanism (e.g., measurements of Ca^{2+} flux or ion efflux). It is also unclear how the response is initiated and whether an effector or other specificity determinants are involved.
12. Lines 416–420: This sentence requires revision.
a) Only a single isolate was tested.
b) The study does not substantially expand the mechanistic repertoire of disease-resistance genes, as mechanistic insight is limited.
c) It is unclear how the study represents an advancement in understanding the role of newly evolved genes in wheat plant–pathogen interactions
13. Line 863: Please describe the methods itself (DAB and FITC WGA staining)

Version 1:

Reviewer comments:

Reviewer #1

(Remarks to the Author)
This revised manuscript made significant revision based on the comments of the reviewers. The authors performed additional experiments and provided strong new information to support their findings. I was satisfied with the scientific meaning and content of the revision. I have no further comments for the revised manuscript.

Reviewer #2

(Remarks to the Author)
The revised manuscript is substantially improved and now addresses several of the major concerns raised previously. The genetic evidence identifying Yr26/Yr24/YrCH42/YrG22 as the same gene is strong and convincing, supported by fine mapping, BAC-based candidate identification, EMS mutant analysis, BSMV silencing, CRISPR knockout, and transgenic complementation. The manuscript is also strengthened by the expanded haplotype analysis, the more careful evolutionary interpretation, and the newly added data supporting a Ca^{2+}-dependent component of Yr26-triggered cell death. In particular, the addition of Ca^{2+} influx measurements in the Yr26 transgenic line and the suppression of Yr26-triggered cell death by $LaCl_3$ meaningfully reinforce the proposed functional model, while the rebuttal appropriately acknowledges that direct evidence for Yr26 itself acting as a cation channel is still lacking.
It is appreciated that the authors have improved the framing of several previously overstated points. The revised manuscript now better resolves the issue of the identity of Yr24/Yr26/YrCH42/YrG22, more cautiously interprets the evolutionary origin of the functional resistant allele after additional screening of wild emmer accessions, and provides new evidence that the resistant Yr26_h1 allele should be distinguished from non-functional or uncertain haplotypes. The added VIGS and F2-based analyses of Yr26_h5 are especially helpful in showing that the resistance phenotype in the corresponding accession is likely due to other gene(s), not Yr26_h5 itself.
A few points still need attention. First, the revised text still contains several internal inconsistencies and residual duplicated passages, especially in the sections describing Yr26 expression under avirulent versus virulent races and the interpretation of Domain 3 function. These need to be carefully reconciled so that the final narrative is fully consistent. Second, although the Ca^{2+}-related evidence is a real improvement, the manuscript should continue to avoid wording that could be interpreted as demonstrating that Yr26 itself forms a Ca^{2+} channel or pore. The current data support a Ca^{2+}-dependent mechanism and are consistent with such a possibility, but they do not yet prove it. Third, the statement that SNP4 is likely the key causal polymorphism distinguishing Yr26_h1 from Yr26_h2 is plausible but still inferential and should remain framed as such. Finally, the manuscript should be checked carefully to ensure that all figure legends fully match the improved statistical reporting described in the rebuttal.

Reviewer #3

(Remarks to the Author)
The authors have done impressive additional work that fully addresses all comments of this reviewer. They performed a substantial amount of new experimental work, including correcting the expression profiles, adding rigorous protein controls, significantly expanding the evolutionary analysis, and successfully tracking real-time cellular calcium dynamics and protein localization. The manuscript text and figures were revised accordingly and are now much stronger. I have no further comments.

Version 2:

Reviewer comments:

Reviewer #2

(Remarks to the Author)
The revised manuscript is substantially improved and now addresses the major concerns raised previously. The evidence identifying Yr26/Yr24/YrCH42/YrG22 as the same gene remains strong and convincing, supported by fine mapping, BAC-based candidate identification, EMS mutant analysis, BSMV silencing, CRISPR knockout, and transgenic complementation. The manuscript is also strengthened by the expanded haplotype and evolutionary analyses, the clarified race-specificity framework, and the added Ca^{2+}-related experiments. In particular, the revised Results and Discussion now present a coherent model in which Yr26 is specifically induced by Pst, confers race-specific resistance, and triggers a Ca^{2+}-dependent cell-death response, while appropriately acknowledging that direct evidence that Yr26 itself functions as a channel is still lacking.

The manuscript also appears to have resolved the previous internal inconsistencies, especially regarding Yr26 expression under avirulent versus virulent races and the interpretation of Domain 3 function. The evolutionary interpretation is more careful than before, and the statement about SNP4 is now framed as a likely rather than proven causal change. These are important improvements.

Only a small number of issues remain. First, the Abstract still states that the C-terminal four-helical bundle domain is critical for triggering cell death "by mediating Ca^{2+} influx," which is somewhat stronger than the more appropriately cautious wording used later in the manuscript. Based on the current evidence, a Ca^{2+}-dependent mechanism is supported, but direct mediation of Ca^{2+} influx by Yr26 itself is not yet demonstrated. Second, the front matter would benefit from one final wording adjustment so that the statements about the emergence of Yr26 are fully aligned with the more careful evolutionary interpretation presented later for the functional resistant allele.

Open Access This Peer Review File is licensed under a Creative Commons Attribution 4.0 International License, which permits use, sharing, adaptation, distribution and reproduction in any medium or format, as long as you give appropriate credit to the original author(s) and the source, provide a link to the Creative Commons license, and indicate if changes were made.
In cases where reviewers are anonymous, credit should be given to 'Anonymous Referee' and the source.
The images or other third party material in this Peer Review File are included in the article's Creative Commons license, unless indicated otherwise in a credit line to the material. If material is not included in the article's Creative Commons license and your intended use is not permitted by statutory regulation or exceeds the permitted use, you will need to obtain permission directly from the copyright holder.
To view a copy of this license, visit https://creativecommons.org/licenses/by/4.0/

REVIEWER COMMENTS

Reviewer #1 (Remarks to the Author):

This manuscript presents a rigorous and impactful study on cloning the wheat stripe rust resistance gene *Yr26*/*Yr24*/*YrCH42*/*YrG22*, a widely used resistance gene in wheat breeding program, and characterizing its function, structure, and evolution. The work is methodologically sound (e.g., map-based cloning, mutant analysis, VIGS, CRISPR-Cas9, transgenic complementation) and fills critical gaps in understanding non-NLR resistance genes in wheat.

Response: Thank you for your recognition of our work. Your careful review and comments were very helpful for us to further improve the work. We agreed with the comments and have responded for them after careful consideration.

Below are comments to improve the current manuscript:

1. Abstract revision suggestion: "*Yr26* is a newly evolved gene encoding a previously uncharacterized transmembrane protein, first emerged in cultivated emmer wheat and recently introgressed into bread wheat. AlphaFold v3.0 predicts a C-terminal four-helical beam domain critical for triggering cell death, a hallmark of hypersensitive resistance."

Response: Thank you for your suggestion. We changed the abstract to "*Yr26* is a newly evolved gene encoding a previously uncharacterized transmembrane protein, first emerged in wild/cultivated emmer wheat and recently introgressed into bread wheat. AlphaFold v3.0 predicts a C-terminal four-helical beam domain is critical for triggering cell death, a hallmark of hypersensitive resistance, by mediating Ca^{2+} influx." (Line 34-44)

2. *Yr24*/*Yr26*/*YrCH42*/*YrG22* were historically proposed as allelic but unconfirmed. The authors may frame the current study's validation of their identity as a major resolution to this uncertainty.

Response: Thank you for your advice. We added a comment "However, it remains

unclear whether these four genes represent the same gene or different alleles." at the end of the third paragraph of the Introduction. (Line 95-96)

3. L177-178: The CDS of *Yr24* in Avocet+Yr24 shares identical coding sequences with *Yr26* in 92R137. L243-244 stated that "An identical *G4* sequence in 92R137, Avocet+Yr24, Chuanmai 42 and Guinong 22 confirmed the same stripe rust resistance gene". Are the sequences of those genes from the 4 lines including the promoter and terminator sequences? Please clarify.

Response: Thank you for your comments. The *G4* sequence cloned in 92R137, Avocet+Yr24, Chuanmai 42 and Guinong 22 comprises a 2945 bp coding region, a 2099 bp promoter and a 758 bp terminator. These details have now been included in the revised manuscript.

4. Up to date, only Rph3 in barley and TaMlo, Lr34, Lr67, Lr14 and WAI-B2 in wheat are transmembrane resistance proteins. The authors could add 1-2 sentences on the paucity of cloned transmembrane resistance genes in the grass family to highlight *Yr26*'s novelty in resistance mechanism.

Response: Thank you for your suggestion. In the revised manuscript, we included a discussion of the research progress on transmembrane resistant proteins in plants. Specifically, "Structural analysis identified *Yr26* as a unique resistance gene harboring two to three transmembrane (TM) helices, thereby differing from classical NLR-type resistance proteins. Emerging evidence increasingly implicates TM proteins as critical components in plant disease resistance. In *Arabidopsis*, the broad-spectrum mildew resistance gene *Rpw8* (Xiao et al. 2001) and the *Plasmodiophora brassicae* resistance gene WeiTsing (WTS) (Wang et al. 2023) both contain TM domains and localize to pathogen-derived membranes to restrict infection. In barley, the leaf rust resistance gene *Rph3* encodes a unique predicted TM protein and is strictly induced by pathogenic fungi during infection (Dinh et al. 2022). In wheat, several TM-domain resistance proteins have been characterized, including TaMlo (powdery mildew resistance) (Li et al. 2022), Lr34/Yr18/Sr57/Pm38 (Krattinger et al. 2009), Lr67/Yr46/Sr55/Pm46 (Moore et al.

2015) and WAI-B2 (broad-spectrum multiple pathogen resistance) (Li et al. 2026), Lr14a (leaf rust resistance) (Kolodziej et al. 2021). Additional TM-containing resistance proteins, such as Pm4 (Sánchez-Martín et al. 2021), Stb16q (Saintenac et al. 2021), and WTK7-TM (Li et al. 2024), possess TM domains that are essential for their membrane localization and function. In this study, an EMS mutant (Mut6) carrying a premature stop codon upstream of the C-terminal TM helices produced a truncated protein lacking an intact Domain 3, resulting in complete loss of resistance. This genetic evidence confirms that the TM domain is indispensable for Yr26 function. The identification of Yr26 further expands the repertoire of TM resistance proteins in wheat and underscores the central role of such proteins in plant-pathogens interactions."

Reference:

Dinh, H.X. et al. The barley leaf rust resistance gene *Rph3* encodes a predicted membrane protein and is induced upon infection by avirulent pathotypes of *Puccinia hordei*. *Nature Communications* **13**, 2386 (2022).

Kolodziej, M.C. et al. A membrane-bound ankyrin repeat protein confers race-specific leaf rust disease resistance in wheat. *Nature communications* **12**, 956 (2021).

Krattinger, S.G. et al. A putative ABC transporter confers durable resistance to multiple fungal pathogens in wheat. *Science* **323**, 1360-1363 (2009).

Li, M. et al. A membrane associated tandem kinase from wild emmer wheat confers broad-spectrum resistance to powdery mildew. *Nature Communications* **15**, 3124 (2024).

Li, S. et al. Genome-edited powdery mildew resistance in wheat without growth penalties. *Nature* **602**, 455-460 (2022).

Moore, J.W. et al. A recently evolved hexose transporter variant confers resistance to multiple pathogens in wheat. *Nature Genetics* **47**, 1494-1498 (2015).

Saintenac, C. et al. A wheat cysteine-rich receptor-like kinase confers broad-spectrum resistance against Septoria tritici blotch. *Nature Communications* **12, 433**(2021).

Sánchez-Martín, J. et al. Wheat Pm4 resistance to powdery mildew is controlled by alternative splice variants encoding chimeric proteins. *Nature Plants* **7**, 327-341 (2021).

Wang, W. et al. WeiTsing, a pericycle-expressed ion channel, safeguards the stele to confer clubroot resistance. *Cell* **186**, 2656-2671.e18 (2023).

Xiao, S. et al. Broad-spectrum mildew resistance in Arabidopsis *thaliana* mediated by RPW8. *Science* **291**, 118-120 (2001).

5. Could Yr26 form a pentameric cation channel as WeiTsing in Arabidopsis? The authors may perform Ca^{2+} influx assays in wheat protoplasts or *Nicotiana benthamiana*

leaves. The Ca^{2+} blocker $LaCl_3$ should be tested in wheat protoplasts or *Nicotiana benthamiana* assays.

Response: Thank you for your insightful suggestions. We performed an experiment to measure the Ca^{2+} influx in mesophyll tissue of the transformation line T_3-17-5 (Fielder+*Yr26*) and WT accession Fielder at 1dpi and 5dpi following inoculation with CYR32 (Fig. 4i; Supplementary Table 5). Plants treated with electrolytic fluorinating solution served as the control. In the control treatment, the Ca^{2+} influx rate in T_3-17-5 (-48.8 and -88.4 pico mol cm^{-2} s^{-1}) was slightly higher than in WT (-38.4 and -59.9 pico mol cm^{-2} s^{-1}), but both remained at relatively low levels at 1 dpi and 5 dpi (Fig. 4i; Supplementary Table 5). Upon CYR32 inoculation, Ca^{2+} influx rate increased dramatically in both T_3-17-5 (-519.3 and -813.0 pico mol cm^{-2} s^{-1}) and WT (-425.4 and -612.5 pico mol cm^{-2} s^{-1}), indicating that *Pst* infection induces Ca^{2+} influx. Compared to the WT, T_3-17-5 exhibited approximately 22.08% and 32.73% faster Ca^{2+} influx rate at 1 dpi and 5 dpi, respectively, suggesting that *Yr26* activates Ca^{2+} influx signals upon *Pst* challenge. Furthermore, when the Ca^{2+} blocker $LaCl_3$ (2 mM) was applied during expression of Yr26 in *Nicotiana benthamiana*, the cell death typically triggered by Yr26 was abolished, confirming the Ca^{2+} plays an important role in the Yr26-mediated immune response (Fig. 4j).

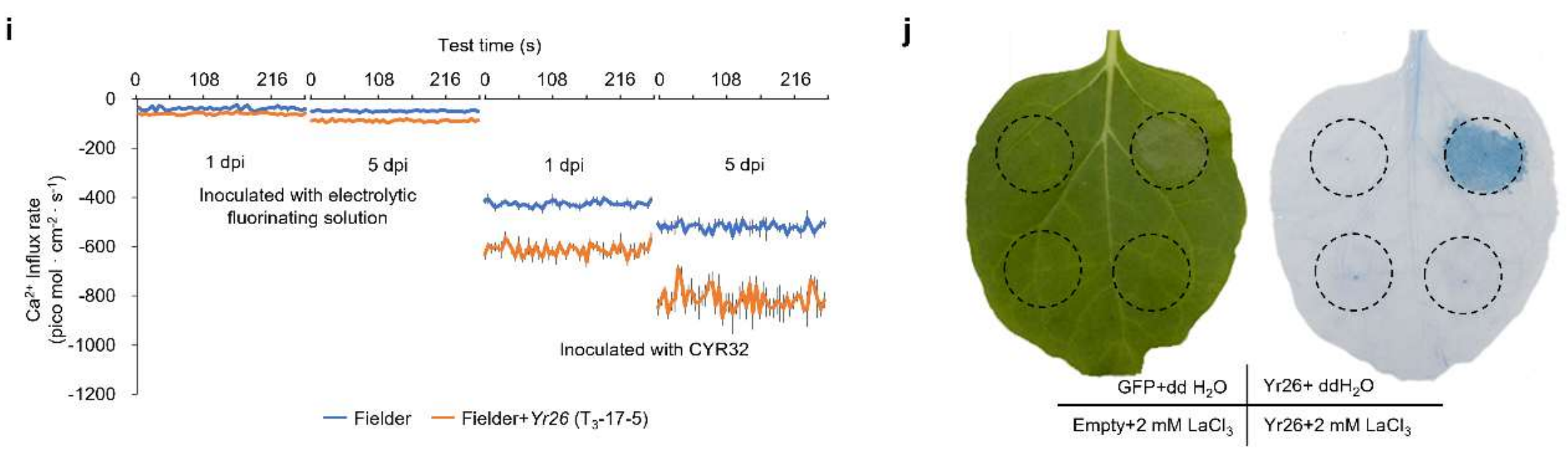

Fig. 4i-j Yr26-mediated resistance requires calcium influx, as shown by NMT analysis and $LaCl_3$ blockade

i. Ca^{2+} influx rate in transgene line T_3-17-5 (Fielder+*Yr26*) and WT line Fielder leaves infected with *Pst* race CYR32 at 1dpi and 5 dpi. Leaves inoculated with electrolytic fluorinating solution served as mock control. For each treatment, Ca^{2+} influx rate was continuously monitored for approximately 5 minutes using Non-invasive Micro-test Technology (NMT), and four independent leaves ($n = 4$) were measured.

j. Suppression of *Yr26* overexpression induced cell death by the calcium channel blocker $LaCl_3$ in *N. benthamiana* leaves. Agrobacterium cultures harboring the indicated constructs (GFP, empty vector, and *Yr26*) were infiltrated into *N. benthamiana leaves*. Eight hours after *Agrobacterium* infiltration, the infiltrated region was further treated with either water or 2 mM $LaCl_3$, a calcium channel blocker. Cell death phenotypes were assessed at 2 days post-infiltration (dpi) using trypan blue staining (dead cells are stained blue). Expression of *Yr26* alone induced a clear cell death phenotype, whereas co-infiltration with $LaCl_3$ abolished this response. No cell death was observed in the GFP, empty vector, or empty vector plus $LaCl_3$ control regions. Similar results were obtained from three independent biological replicates (n = 3).

Although our data demonstrate that Yr26-mediated immunity requires Ca^{2+} influx, and $LaCl_3$ blocks Yr26-triggered cell death, the direct evidence for Yr26 forming a pentameric cation channel like WeiTsing is still missing. Future structural and electrophysiological studies will be needed to determine whether Yr26 itself functions as a Ca^{2+}-permeable channel or acts upstream of such channels.

6. The suppressed recombination in the *Yr24*/*Yr26* genomic region should be noted. The fine genetic mapping interval of the candidate gene overlaps the centromeric region of chromosome 1B, a known recombination cold spot, to justify why further refinement beyond 0.002 cM was unfeasible.

Response: Thank you for your comment and suggestion. We revised the sentence "Suppressed recombination that precluded further refinement was observed in the candidate interval (Supplementary Figure 1c)" to:

"Further refinement of the *Yr26* candidate interval was precluded by a severe suppression of recombination within the mapped region. All remaining recombination events were asymmetrically clustered: on the proximal flank, all events mapped within a ~14 kb interval between markers *N726* and *N740*; on the distal flank, all events mapped within a ~127 kb interval between markers *N1940* and *N2068* (Supplementary Fig. 1c). Notably, no recombination was detected across the entire candidate interval spanning 326.01–327.35 Mb (IWGSC Chinese Spring (CS) RefSeq v1.0). This asymmetric clustering and the complete absence of recombination across the physical interval are characteristic of centromeric recombination suppression. Structural

variation (e.g., chromosomal inversion) was excluded as an alternative cause, because high collinearity is maintained across diverse wheat genome assemblies in this region (Supplementary Fig. 2). Consequently, the *Yr26* interval could not be narrowed beyond 0.002 cM by genetic mapping alone."

In addition, we have added a discussion on how to overcome suppressed recombination using BAC-based strategies, which now reads:

"*Yr26* was fine-mapped to a recombination-suppressed interval in the near centromeric region of chromosome 1B. In such regions, combining target region assembly with mutant analysis has proven effective for gene isolation. For example, a chromosome-scale assembly of the South African bread wheat cultivar Kariega, combined with EMS-induced mutational analysis, enabled the isolation of *Yr27* (Athiyannan et al. 2022), which encodes a coiled-coil NBS-LRR immune receptor. In this study, we assembled the *Yr26* target region using a BAC-by-BAC strategy, as demonstrated for *Sr33* (Periyannan et al. 2013). Transcript-based annotation and RNA-seq analysis of six independent EMS-derived susceptible mutants identified *G4* as the sole candidate gene. In parallel, MutChromSeq analysis of five susceptible mutants derived from the Avocet+*Yr24* background independently identified the same gene, confirming that *Yr24* and *Yr26* are identical. More broadly, mutant-based cloning strategies have become invaluable for isolating resistance genes in recombinationally inert genomic regions, particularly in crops with complex or incomplete reference genomes. Notable examples include MutRenSeq for *Yr5* (Marchal et al. 2018), MutIso-seq for *Yr9* (Zhang et al. 2025), and sequencing trait-associated mutations (STAM) for *Yr10* (Ni et al. 2023) in polyploid wheat. The successful cloning of *Yr26* further demonstrates the power of integrating genome assembly with systematic mutant analysis to overcome the challenges posed by centromeric recombination suppression."

Thank you again for your insightful comment.

Reference

Athiyannan, N. et al. Long-read genome sequencing of bread wheat facilitates disease resistance gene

cloning. *Nature Genetics* **54**, 227-231 (2022).
Marchal, C. et al. BED-domain-containing immune receptors confer diverse resistance spectra to yellow rust. *Nature Plants* **4**, 662-668 (2018).
Ni, F. et al. Sequencing trait-associated mutations to clone wheat rust-resistance gene *YrNAM*. *Nature Communications* **14**, 4353 (2023).
Periyannan, S. et al. The gene *Sr33*, an ortholog of barley Mla genes, encodes resistance to wheat stem rust race Ug99. *Science* **341**, 786-788 (2013).
Zhang, Y. et al. Rapid isolation of *Yr9* via MutIsoSeq and QTL analysis of durable stripe rust resistance in wheat cultivar Xingzi 9104. *Stress Biology* **5**, 29 (2025).

7. The single *Yr26_h5* accession (CAS075) is resistant to both avirulent (CYR32) and virulent (CYR34) races. Could the authors rule out the possibility of other gene, rather than the *Yr26_h5*, contribute to the resistance? A follow-up study (e.g., complementation with *Yr26_h5* or VIGS of *Yr26_h5*) will test if it confers broad-spectrum resistance, as this is a high-impact observation.

Response: Thank you for your comments. To test whether *Yr26_h5* functions in resistance, we performed a VIGS experiment on CAS075 (DEW075) using the vector BSMV:*G4*-γ1as. No significant difference in stripe rust response to race CYR32 was observed between the silenced plants and the mock or control groups (Fig. 5d). qRT-PCR analysis confirmed that *Yr26_h5* transcript levels were significantly reduced in silenced leaves compared to mock controls (Fig. 5e). Due to limited seed availability, we were unable to test the virulent race CYR34 at this stage. In parallel, an association analysis was performed using an F_2 population derived from a cross between CAS075 and the susceptible accession D026 (Fig. 5f). Based on the DM2 functional marker of *YR26*, no stripe rust phenotypic distinction (to CYR32) was detected between the *Yr26_h5* (n=92) and *yr26_h5* (n=31) lines. These findings indicate that *Yr26_h5* is not required for resistance to the avirulent race CYR32 in CAS075 and we speculate that another gene(s) likely underlies the observed broad-spectrum resistance.

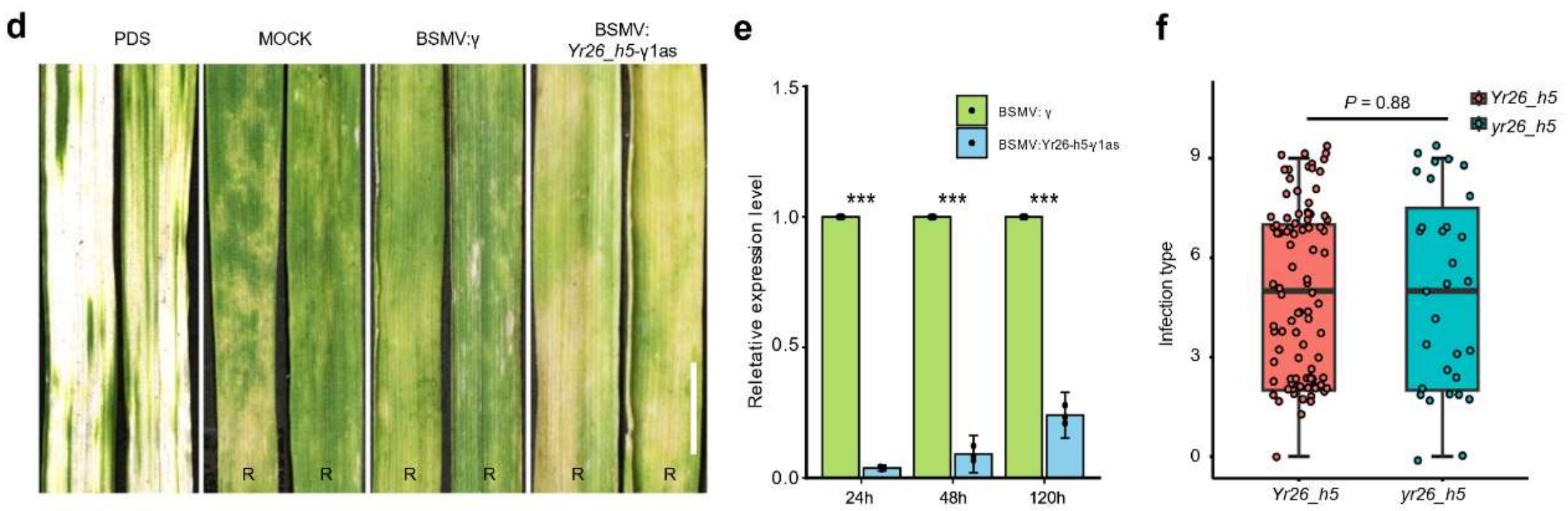

Fig.5 d-f Functional analysis of the *Yr26_h5* allele in stripe rust resistance using VIGS and an F_2 genetic population

d. Representative images showing results of the VIGS experiment. Wheat leaves inoculated with BSMV:*TaPDS*-γas (positive control for silencing), BSMV:γ (empty vector control), and BSMV: *Yr26_h5* -γ1as (silencing constructs that target *G4*), respectively. Mock, wheat leaves treated with 1× FES buffer. BSMV:*TaPDS*-γas denote controls with a PDS silencing construct. BSMV: *Yr26_h5* -γ1as denote different silencing constructs that target *Yr26_h5*. Phenotypes for BSMV:*TaPDS*-γas plants were recorded at 10 dpi. Reactions of *Yr26_h5*-knockdown, mock and control plants inoculated with avirulent *Pst* race CYR32 at 14 dpi. R, resistant; S, susceptible. Scale bars, 0.5 cm.**e.** Quantification of silencing efficiency for *Yr26_h5* in VIGS plants. Data are shown as means ± s.d. (n = 3 biological replicates). Each replicate is shown as a single data point. Gene *TaEF-1α* was used for normalization. The transcript level of *Yr26_h5* in the control leaves at 0 hpi was set as 1. Asterisks indicate significant differences compared to the control (BSMV:γ) as assessed by two-tailed Student's *t*-tests. ***$P < 0.001$.

f. The distribution and comparison of infection types to stripe rust race CYR32 for each genotype group at the *Yr26_h5* locus in DEW075/D026 F_2 population. The numbers of F_2 plants classified as genotypes *Yr26_h5* and *yr26_h5* were 92 and 30, respectively. No significant (ns) difference was detected between the two groups. The central line within each violin/box represents the median, and the edges indicate the interquartile range. Each data point is shown as a dot. Statistical significance was determined using two-tailed Student's *t*-tests and a $P > 0.05$ was considered not significant.

In addition, we are currently generating stable transgenic lines of *Yr26_h5* in a susceptible accession Fielder to more definitively assess its role in resistance. the ongoing transgenic complementation will address whether *Yr26_h5* confers broad-spectrum resistance.

8. Why both G4-specific fragments (1as/2as) were used in the VIGS experiment? Do both of them achieved similar knockdown efficiency (~50%)?

Response: Thank you for your thoughtful question. Given the limitations of VIGS, such as incomplete silencing, risk of off-target, two independent *G4*-specific silencing fragments (1as and 2as) were selected for of G4 to ensure the reliability of the results. Both fragments achieved >50% knockdown efficiency, as determined by qRT-PCR and

shown in the revised Fig. 3c, which is sufficient for effective silencing of the target gene in the VIGS system. As both fragments yielded consistent phenotypes and due to seed availability, we selected fragment 1as for VIGS to validate *Yr26_h5* function in CAS075. This design is now clarified in the Methods section.

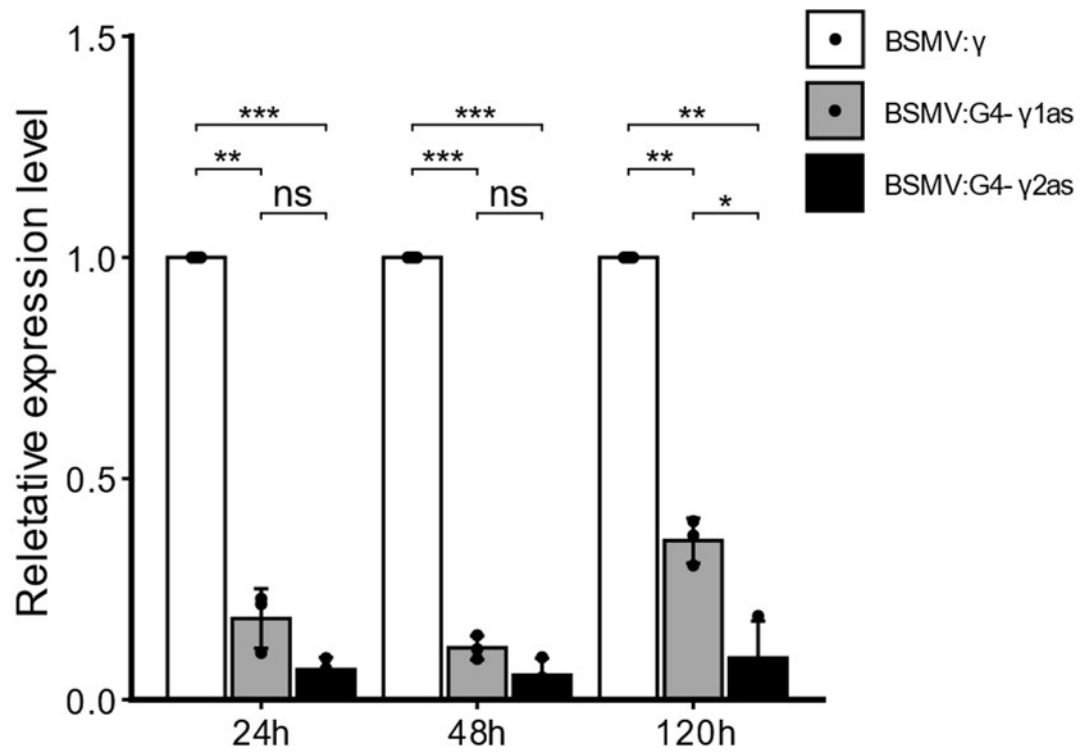

Fig. 3c Quantification of silencing efficiency for *G4* in VIGS plants. Gene *TaEF-1α* was used for normalization. The transcript level of *G4* in the control leaves at 0 hpi was set as 1. Data are shown as mean ± s.d. ($n = 3$ biological replicates). Each replicate is shown as a single data point. Asterisks indicate significant differences compared to the control (BSMV:γ) as assessed by two-tailed Student's *t*-tests. *$P < 0.05$, **$P < 0.01$, ***$P < 0.001$; ns, not significant.

9. The authors speculate that *Yr26* evolved in cultivated emmer. However, the authors only screened 33 wild emmer (WEW) accessions that may be not enough evidence to exclude wild emmer as its origin.

Response: Thank you for the comment. To more rigorously investigate the origin of *Yr26*, we expanded our screening to include 359 wild emmer (WEW) accessions kindly provided by Prof. Lin Huang at SAU. Based on the *DM2* functional marker, we identified five novel accessions carrying the *YR26* locus. Haplotype analysis revealed a novel haplotype in these accessions, designated *Yr26_h6* (Supplementary Table 4). Compared with the functional allele *Yr26_h1*, *Yr26_h6* contains, in addition to SNP1-4 and the 12-bp indel, four additional SNPs and a 289 bp insertion (Big indel1) (Supplementary Fig. 13). All five accessions carrying *Yr26_h6* are susceptible to both CYR32 and CYR34 (Supplementary Table 4), indicating that this haplotype does not confer resistance. These findings identify a non-functional *Yr26* haplotype in wild emmer and provide new evidence regarding the evolutionary origin of *Yr26*, suggesting that the functional *Yr26* allele may have evolved before domestication. We have

incorporated these new data into the revised manuscript and have revised the conclusion to state that functional *Yr26* allele originated from cultivated emmer or unsampled wild emmer accessions.

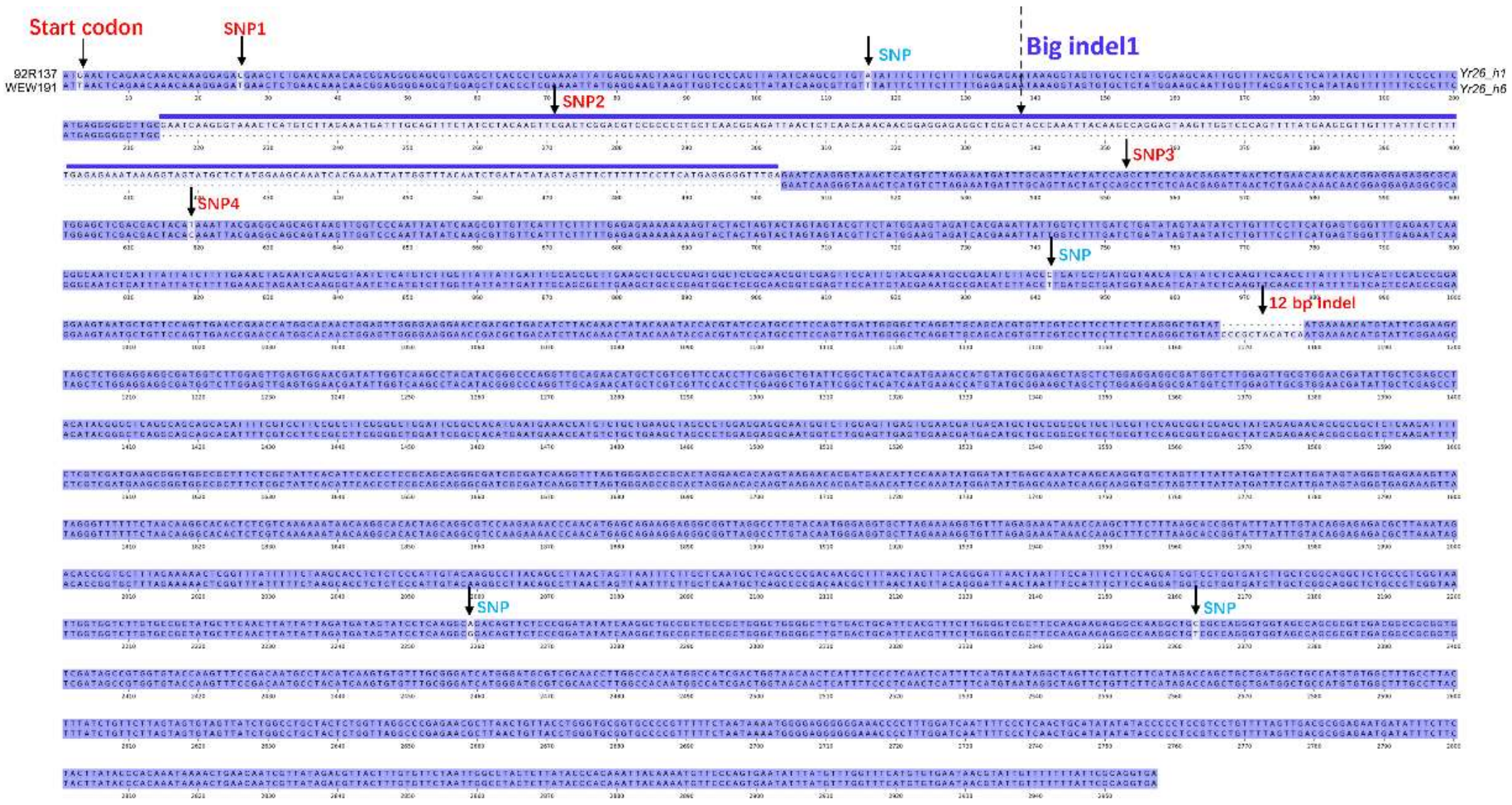

Supplementary Figure 13 Alignment of *Yr26_h1* in 92R137 and *Yr26_h6* in WEW191. Blue bar, Indel1. SNPs in haplotypes *Yr26_h1* – *_h5* are marked by black arrows with red font. In contrast, SNPs unique to the *Yr26_h1* and *Yr26_h6* comparison are indicated by black arrows with blue font.

10. “To characterize Yr26-mediated resistance seedlings of lines 92R137…” should be “To characterize Yr26-mediated resistance, seedlings of lines 92R137…”

Response: Thank you for your comment. We revised it.

Reviewer #2 (Remarks to the Author):

This study map-clones *Yr26* using a large NIL-based F_2 population (8,046 plants) and narrows the locus to a 0.002 cM interval supported by BAC contig assembly and candidate transcript annotation, ultimately prioritizing an unannotated transcript (*G4*) as the leading candidate. The authors provide multiple lines of functional evidence that *G4* encodes *Yr26*, including transgene complementation in Fielder, where *G4*-positive lines are resistant and null/Wt lines are susceptible. They further propose that *Yr26* is a previously uncharacterized transmembrane (TM) orphan gene whose C-terminal four-helix beam domain (Domain3) is required for hypersensitive response (HR)/cell death, based on AlphaFold predictions and truncation/fragment assays. Evolutionary analyses

suggest *Triticeae*-restricted distribution and recent origin consistent with an orphan gene.

Study design, controls, and whether conclusions are supported

Overall, the study design is appropriate for the central research question—identifying the causal gene underlying *Yr26*-mediated stripe rust resistance and characterizing its key functional domain—because it combines:

1. high-resolution mapping with a large segregating population and physical contig support, and

2. orthogonal genetic tests (mutants, silencing/editing, and complementation; complementation evidence is explicit in the main text).

Controls are generally well chosen for the mechanistic assays: empty-vector negatives and well-established positives are used in the wheat protoplast readout (*Sr50*/*AvrSr50*) and *N. benthamiana* HR assays (BAX). For expression profiling, the use of avirulent vs virulent Pst races and mock controls appropriately supports the conclusion that *Yr26* is pathogen-inducible during incompatible interaction.

Are conclusions supported?

• The conclusion that *G4* is *Yr26* is well supported by genetic mapping + complementation.

• The conclusion that Domain3 is necessary/sufficient to trigger HR-like cell death is supported by fragment overexpression phenotypes in two systems (wheat protoplasts and tobacco), with consistent negative controls.

• The manuscript's broader mechanistic speculation that *Yr26* may function like a cation channel facilitating Ca^{2+} influx is explicitly framed as a hypothesis and "future research". However, in its current form, this part should remain clearly labeled as speculative unless additional functional/channel data are provided (see Major points).

Response: Thank you for your comments. Your constructive comments have guided us

in further strengthening the manuscript, as detailed in our point-by-point responses below.

Methodological soundness and reproducibility

Most core methods are described at a level that enables replication for mapping, cell death assays, and plant-pathogen phenotyping, including key parameters and controls (e.g., construct amounts and incubation for protoplasts; OD600 and buffer composition for infiltration; staining and microscopy approach). The mapping strategy and candidate interval definition are also described clearly (markers, recombinants, interval definition), though some practical details would still help reproducibility/interpretation (see below).

Response: Thank you for your comments. In the revised manuscript, we have carefully reviewed the Methods section and added practical details that we had inadvertently omitted or only briefly mentioned. Specifically, we have now included:

(1) The complete primer sequences for all KASP markers, PCR markers, and VIGS fragments in Supplementary Table 12, along with their genomic positions.

(2) Detailed PCR cycling conditions for KASP genotyping, including the touchdown phase parameters and final extension steps.

(3) The exact composition of the FES buffer used for BSMV inoculations (500 mM glycine, 300 mM K_2HPO_4, 1% sodium pyrophosphate, 1% bentonite, 1% celite, pH 8.5).

(4) The specific parameters for confocal microscopy, including laser wavelengths and detection ranges for GFP and mCherry fluorescence.

(5) The complete protocol for protoplast isolation and transformation, including enzyme concentrations, incubation times, and centrifugation conditions.

(6) The accession numbers and database versions used for all BLAST searches, including the specific Ensembl release versions and NCBI database build dates.

We believe these additions substantially improve the reproducibility of our methods for

other researchers.

Statistics and uncertainties

Some sections report biological replication (e.g., “three biological replicates” for protoplast assays; “>30 infection sites” for microscopy), which is a good baseline. That said, the manuscript would benefit from a clearer, centralized statistical description: what statistical tests were used for each figure type, what “n” represents (plants, leaves, infection sites, independent experiments), whether experiments were repeated independently, and whether assumptions were met. At present, the statistical reporting appears distributed and may be insufficient for *Nature Communications* standards unless fully detailed in figure legends/reporting summary.

Response: Thank you for your comments. We fully agree with this important point. We have made the following improvements in the revised manuscript:

(1) We have added a dedicated "Statistical analysis" subsection in the Reporting Summary, which includes:

a) Two-group comparisons were performed using the two-tailed Student's *t*-test function from the rstatix package (version 0.7.3).

b) For comparisons among three or more groups, one-way ANOVA was conducted via the aov function from the base R stats package, followed by post-hoc multiple comparisons using Tukey’s HSD test.

c) Significance levels were indicated as follows: $*P < 0.05$, $**P < 0.01$, $***P < 0.001$.

d) The software used for all analyses was R v4.2.0.

e) The definition of "n" for each experiment type (e.g., n = number of biologically independent plants; n = number of infection sites; n = number of independent experiments as specified in each figure legend).

(2) Revised figure legends

We have systematically revised all figure legends to explicitly report:

a) The sample size (n) and what it represents (e.g., "$n = 3$ biologically independent plants," "$n = 30$ independent infection sites").

b) The number of independent experimental replicates.
c) The statistical test used and the *P*-value thresholds.
d) Data are shown as mean ± s.d. and all raw data points are plotted directly on the bar graph, allowing visualization of data distribution.

We trust that these revisions now meet the statistical reporting standards expected by Nature Communications.

Major points (recommended revisions)

1. Mechanistic claims should be aligned with evidence (channel/Ca^{2+} hypothesis).

The discussion draws an analogy to WTS and suggests a cation-channel-like mechanism and Ca^{2+} influx. This is interesting, but the current evidence base is primarily: (i) AlphaFold-predicted TM/helix architecture and (ii) HR induction by Domain3 fragments.

Actionable fix: Either (a) add direct supporting assays (e.g., subcellular localization; oligomerization; Ca^{2+} reporter readouts after Yr26/Domain3 expression; mutational analysis of conserved residues in Domain3), or (b) tighten the text to clearly label this as a hypothesis and avoid mechanistic phrasing that implies demonstrated channel activity.

Response: Thank you for your thoughtful comments and suggestions regarding the mechanistic model. We agree that the channel/Ca^{2+} hypothesis requires explicit support, and we have now provided additional experimental evidence.

Subcellular localization We attempted to determine the subcellular localization of Yr26 via transient expression in wheat protoplasts and *N. benthamiana* leaves. However, multiple attempts at protein extraction and localization failed, likely due to the low expression level of Yr26 or its hypersensitivity to necrosis. As an alternative approach, we took advantage of the M15 mutant, which carries a point mutation that compromises resistance but does not completely abolish the cell death function, suggesting that the M15 protein may retain normal biochemical activity (Fig. 4g-h). We therefore used the M15 sequence for subcellular localization analysis. The results showed that

M15:Yr26^{S441N} and M15:Domain3^{S441N} localized to the plasma membrane, with TAWPI6-mCherry serving as a membrane marker (Koike et al. 2005). In contrast, Domain1+Domain2 (lacking Domain3) exhibited both nuclear and cytoplasmic membrane localization (Supplementary Fig. 9). These findings provide further evidence that Yr26 functions as a transmembrane protein.

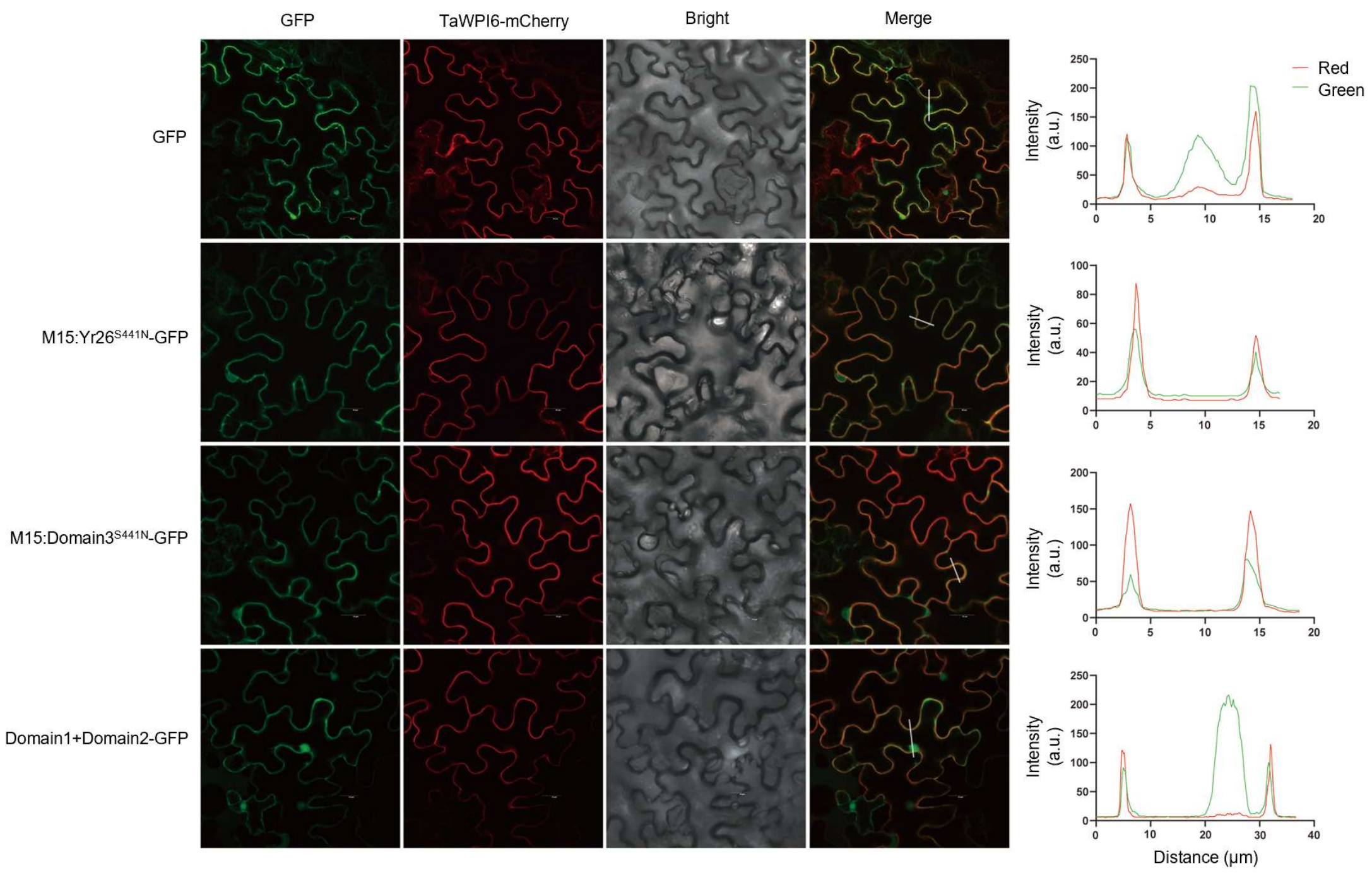

Supplementary Figure 9 Subcellular localization of Yr26 and its predicted domains in *N. benthamiana* epidermal cells. C-terminal GFP fusion proteins were generated using the pYJ-GFP-Nos vector and were transiently expressed in *N. benthamiana* leaves. TaWPI6-mCherry were co-infiltrated served as a cytoplasmic membrane localization marker. GFP (green) and mCherry (red) fluorescence signals were observed, along with bright-field (Bright) and merged (Merge) images. The fluorescence intensity profiles (right) quantify the relative fluorescence intensity (a.u.) along the white lines shown in the merge images, with distance measured in micrometers (μm). For each treatment, at least three independent cells were examined.

Reference:

Koike, M. et al. Molecular characterization of a cold-induced plasma membrane protein gene from wheat. *Molecular Genetics and Genomics* **274**, 445-453 (2005).

Ca^{2+} influx assays We measured the Ca^{2+} influx in mesophyll tissue of the transformation line T_3-17-5 (Fielder+*Yr26*) and WT accession Fielder at 1dpi and 5dpi following inoculation with CYR32 (Fig. 4i). Plants treated with electrolytic fluorinating solution served as the control. In the control treatment, the Ca^{2+} influx rate in T_3-17-5

(-48.8 and -88.4 pico mol cm^{-2} s^{-1}) was slightly higher than in WT (-38.4 and -59.9 pico mol cm^{-2} s^{-1}), but both remained at relatively low levels at 1 dpi and 5 dpi (Fig. 4i; Supplementary Table 5). Upon CYR32 inoculation, Ca^{2+} influx rate increased dramatically in both T_3-17-5 (-519.3 and -813.0 pico mol cm^{-2} s^{-1}) and WT (-425.4 and -612.5 pico mol cm^{-2} s^{-1}), indicating that *Pst* infection induces Ca^{2+} influx. Compared to the WT, T_3-17-5 exhibited approximately 22.08% and 32.73% faster Ca^{2+} influx rate at 1 dpi and 5 dpi, respectively, suggesting that *Yr26* activates Ca^{2+} influx signals upon *Pst* challenge. Furthermore, when the Ca^{2+} blocker $LaCl_3$ (2 mM) was applied during expression of Yr26 in *Nicotiana benthamiana*, the cell death typically triggered by Yr26 was abolished, confirming the Ca^{2+} plays an important role in the Yr26-mediated immune response (Fig. 4j).

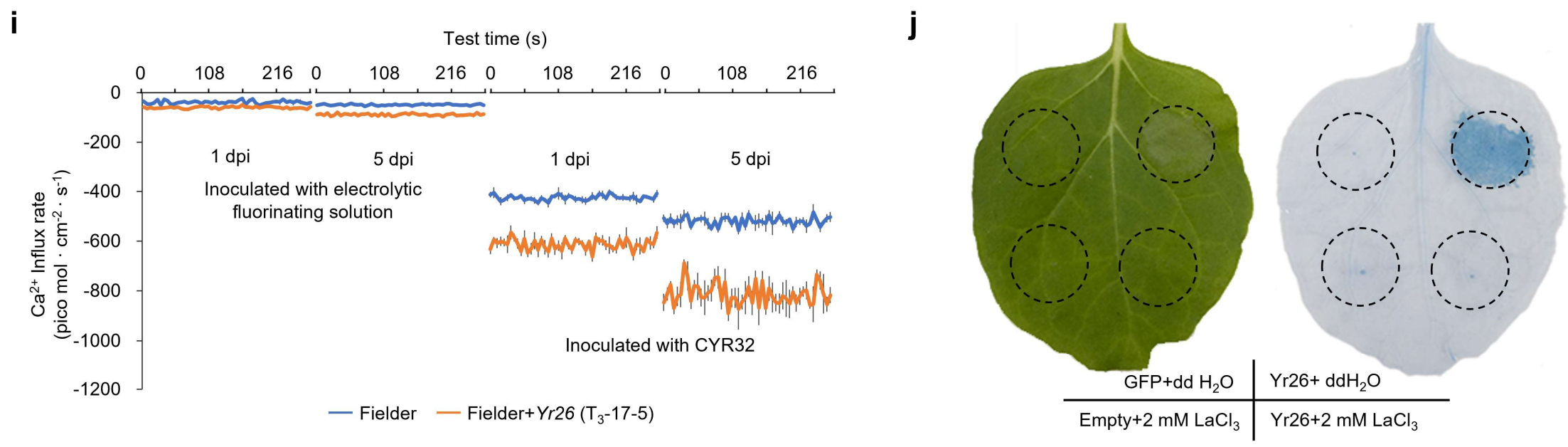

Fig. 4i-j Yr26-mediated resistance requires calcium influx, as shown by NMT analysis and $LaCl_3$ blockade

i. Ca^{2+} influx rate in transgene line T_3-17-5 (Fielder+*Yr26*) and WT line Fielder leaves infected with *Pst* race CYR32 at 1dpi and 5 dpi. Leaves inoculated with electrolytic fluorinating solution served as mock control. For each treatment, Ca^{2+} influx rate was continuously monitored for approximately 5 minutes using Non-invasive Micro-test Technology (NMT), and four independent leaves (n = 4) were measured.

j. Suppression of *Yr26* overexpression induced cell death by the calcium channel blocker $LaCl_3$ in *N. benthamiana* leaves. Agrobacterium cultures harboring the indicated constructs (GFP, empty vector, and *Yr26*) were infiltrated into *N. benthamiana leaves*. Eight hours after *Agrobacterium* infiltration, the infiltrated region was further treated with either water or 2 mM $LaCl_3$, a calcium channel blocker. Cell death phenotypes were assessed at 2 days post-infiltration (dpi) using trypan blue staining (dead cells are stained blue). Expression of *Yr26* alone induced a clear cell death phenotype, whereas co-infiltration with $LaCl_3$ abolished this response. No cell death was observed in the GFP, empty vector, or empty vector plus $LaCl_3$ control regions. Similar results were obtained from three independent biological replicates (n = 3).

Taken together, these data demonstrate that *Yr26* is a plasma membrane-localized

transmembrane protein and that *Yr26*-mediated immunity requires Ca^{2+} influx. However, we acknowledge that direct evidence for *Yr26* itself forming a pentameric cation channel as WeiTsing is currently lacking. We have therefore tightened the discussion to clearly state that the channel-like mechanism remains a hypothesis based on structural prediction (AlphaFold), transmembrane architecture, and functional Ca^{2+} dependency, rather than a demonstrated activity. These observations raise the possibility that Yr26 may function as part of an ion channel complex facilitating Ca^{2+} influx, analogous to WTS. And direct structural and electrophysiological evidence is required to test this hypothesis.

2. Clarify scope and robustness of disease-resistance phenotyping.

The paper asserts Yr26 inhibits post-infection fungal development based on microscopy differences that emerge at 5–7 dpi.

Actionable fix: Explicitly report sample sizes for phenotyping (plants/replicates per genotype/line), number of independent infection experiments, and quantitative metrics (e.g., uredinia density, fungal biomass, infection type scoring) aligned with the images. This will strengthen the causal chain from gene → HR → restricted colonization.

Response: Thank you for your constructive comment. To clarify the scope and robustness of our disease-resistance phenotyping, we have conducted additional histological analyses and quantified key metrics during the *Pst* infection process in NIL-R and NIL-S.

Specifically, we performed time-course diaminobenzidine (DAB) staining to monitor reactive oxygen species (ROS) accumulation from 12 hpi to 72 hpi. Strong ROS accumulation was observed at infection sites in NIL-R compared to NIL-S at 48 hpi and 72 hpi, whereas no significant difference was detected at 12 hpi and 24 hpi (Fig. 1b-c). In addition, we quantified *Pst* fungal biomass in wheat leaf tissues. The results showed comparable fungal development between NIL-R and NIL-S during early infection (1–4 dpi) (Fig. 1d-e). However, at 5–7 dpi, NIL-R exhibited a markedly reduced fungal biomass compared to NIL-S (Fig. 1c).

These quantitative data, including ROS accumulation dynamics and fungal biomass measurements, provide robust evidence supporting that *Yr26* inhibits post-infection fungal development during the late infection stage. The sample sizes and replicate numbers have been explicitly reported in the revised figure legends.

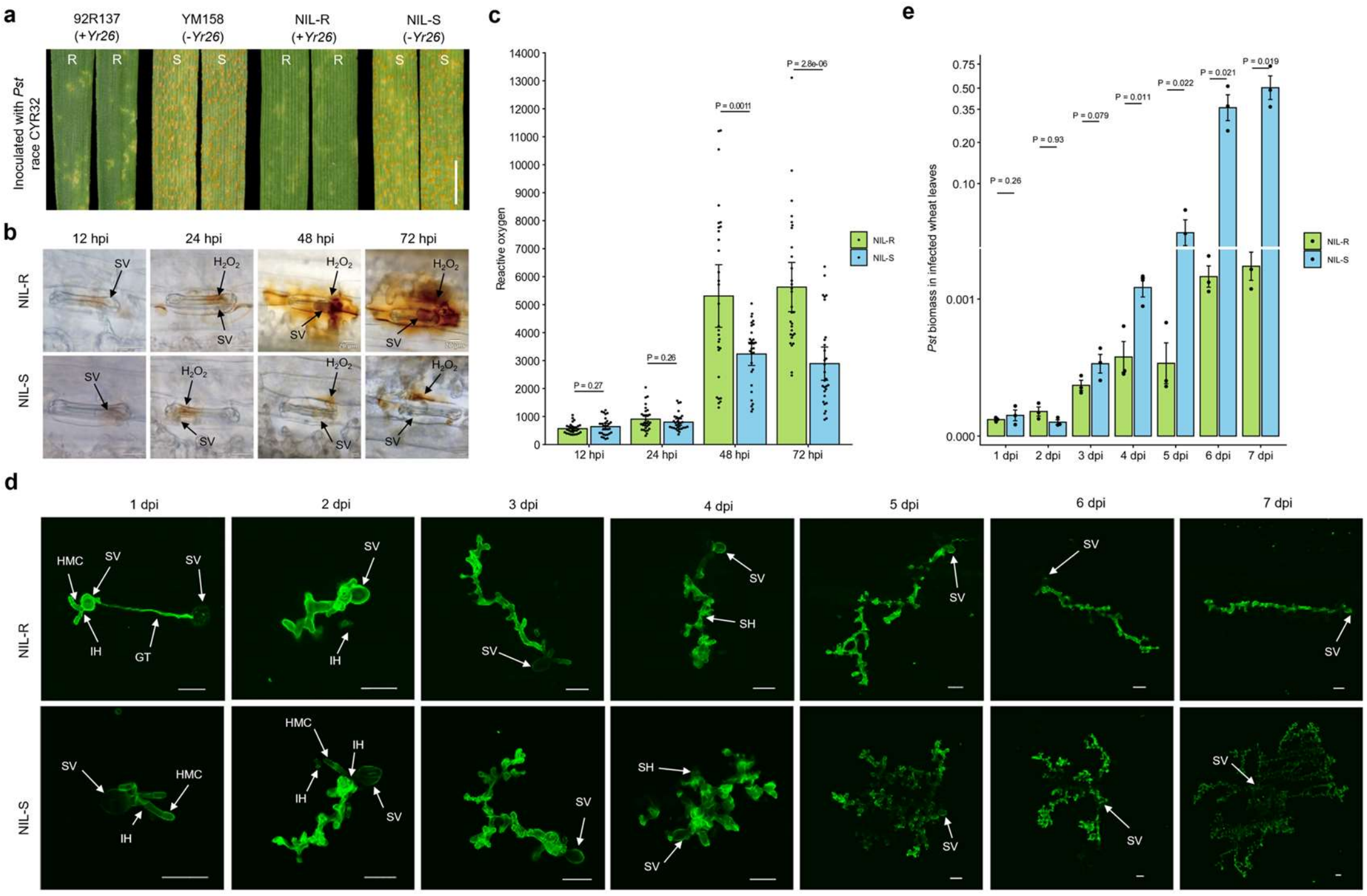

Fig. 1 Resistance response of *Yr26* to avirulent *Puccinia striiformis* f. sp. *tritici* (*Pst*) race CYR32

a. Wheat lines 92R137 (+*Yr26*), YM158 (-*Yr26*), and the near-isogenic line pair NIL-R: +*Yr26*; NIL-S: -*Yr26* inoculated with avirulent *Pst* race CYR32 at 14 days post inoculation (dpi). R: resistant, S: susceptible. Scale bar, 1 cm.

b-c. Histology and statistic of reactive oxygen production in *Yr26* near-isogenic lines inoculated with CYR32 at 12 hpi, 24 hpi, 48 hpi and 72 hpi. SV: substomatal vesicle. Data are shown as mean ± s.d. (n = 30 independent infection sites). Each infection site is shown as a single data point. Statistical significance was determined using two-tailed Student's t-tests and a P value > 0.05 was considered not significant.

d. Histology and cytology of fungal development during incompatible (resistant) and compatible (susceptible) interactions at 1-7 dpi. S, urediniospore; SV: substomatal vesicle; GT, germ tube; IH, infection hypha; HMC, haustorial mother cell; H, haustoria. $n > 30$ independent infection sites. Scale bars, 40 μm.

e. Biomass of *Pst* race CYR32 in infected leaves of *Yr26* near-isogenic lines. Data are shown as mean ± s.d. ($n = 3$ biological replicates). Each replicate is shown as a single data point. Statistical significance was determined using two-tailed Student's t-tests and a $P > 0.05$ was considered not significant.

3. Map-based cloning section: explain "suppressed recombination" implications.

The manuscript notes suppressed recombination preventing further refinement.

Actionable fix: Provide more detail on recombination suppression evidence (e.g., local recombination rate vs flanking regions; structural variation possibility), and clarify how this limitation was overcome by BAC contigs/transcript evidence and mutant/complementation tests.

Response: Thank you for your comment and suggestion. In the revised manuscript, we have provided more detailed evidence for the suppressed recombination. Specifically, the fine mapping interval of Yr26 resides in the near-centromeric region of chromosome 1B, a known recombination cold spot. Micro-collinearity analysis across different wheat genomes revealed no structural genomic variations caused by chromosomal rearrangements, further supporting that the suppressed recombination is due to the centromeric location of the Yr26 interval.

We revised the sentence "Suppressed recombination that precluded further refinement was observed in the candidate interval (Supplementary Figure 1c)" to "Suppressed recombination that precluded further refinement was observed in the candidate interval, as all recombination events were clustered within the interval between markers N726 and N740 (approximately 14 kb apart) or between N1940 and N2068 (approximately 127 kb apart) (Supplementary Figure 1c). This suppression is likely due to its location in the centromeric recombination cold spot on chromosome 1B, rather than structural genomic variations, as evidenced by the high collinearity across different wheat genomes (Supplementary Figure 2)".

In addition, we discuss how to overcome suppressed recombination using BAC-based strategies. "*Yr26* was fine-mapped to a recombination-suppressed interval in the near centromeric region of chromosome 1B. In such regions, combining target region assembly with mutant analysis has proven effective for gene isolation. For example, a chromosome-scale assembly of the South African bread wheat cultivar Kariega, combined with EMS-induced mutational analysis, enabled the isolation of *Yr27* (Athiyannan et al. 2022), which encodes a coiled-coil NBS-LRR immune receptor. In

this study, we assembled the *Yr26* target region using a BAC-by-BAC strategy, as demonstrated for *Sr33* (Periyannan et al. 2013). Transcript-based annotation and RNA-seq analysis of six independent EMS-derived susceptible mutants identified *G4* as the sole candidate gene. In parallel, MutChromSeq analysis of five susceptible mutants derived from the Avocet+*Yr24* background independently identified the same gene, confirming that *Yr24* and *Yr26* are identical. More broadly, mutant-based cloning strategies have become invaluable for isolating resistance genes in recombinationally inert genomic regions, particularly in crops with complex or incomplete reference genomes. Notable examples include MutRenSeq for *Yr5* (Marchal et al. 2018), MutIso-seq for *Yr9* (Zhang et al. 2025), and sequencing trait-associated mutations (STAM) for *Yr10* (Ni et al. 2023) in polyploid wheat. The successful cloning of *Yr26* further demonstrates the power of integrating genome assembly with systematic mutant analysis to overcome the challenges posed by centromeric recombination suppression."

Reference

Athiyannan, N. et al. Long-read genome sequencing of bread wheat facilitates disease resistance gene cloning. *Nature Genetics* **54**, 227-231 (2022).

Marchal, C. et al. BED-domain-containing immune receptors confer diverse resistance spectra to yellow rust. *Nature Plants* **4**, 662-668 (2018).

Ni, F. et al. Sequencing trait-associated mutations to clone wheat rust-resistance gene *YrNAM*. *Nature Communications* **14**, 4353 (2023).

Periyannan, S. et al. The gene *Sr33*, an ortholog of barley Mla genes, encodes resistance to wheat stem rust race Ug99. *Science* **341**, 786-788 (2013).

Zhang, Y. et al. Rapid isolation of *Yr9* via MutIsoSeq and QTL analysis of durable stripe rust resistance in wheat cultivar Xingzi 9104. *Stress Biology* **5**, 29 (2025).

4. AlphaFold confidence and domain definitions.

The structural prediction reports lDDT max 0.57 for Domain3.

Actionable fix: Provide per-residue confidence or a more nuanced interpretation (e.g., which segments are high-confidence helices vs uncertain loops), and ensure the functional conclusions are not over-weighted on low-confidence regions. A simple supplementary figure mapping AlphaFold confidence to Domain boundaries would help.

Response: Thank you for your comment and suggestion. In response to your suggestion, we have added the per-residue confidence details of the predicted AlphaFold structure in Supplementary Fig. 8. We acknowledge that, as Yr26 is an orphan gene without closely related templates, the overall confidence of the structural prediction is modest. However, Domain 3, and particularly its predicted transmembrane helix, shows a relatively higher level of confidence compared to other regions. Importantly, our subcellular localization analysis (Supplementary Fig. 7) independently confirms that Yr26 is a transmembrane protein and that its membrane localization is determined by Domain 3 (Domain3 localized to the plasma membrane, whereas Domain1+Domain2 did not). Thus, while the absolute structural coordinates remain predictive, the key conclusion that Yr26 is a membrane-anchored protein via Domain 3 is supported by experimental evidence. We have revised the text to avoid over-interpreting low-confidence regions and to clearly distinguish between structural predictions and experimentally validated features.

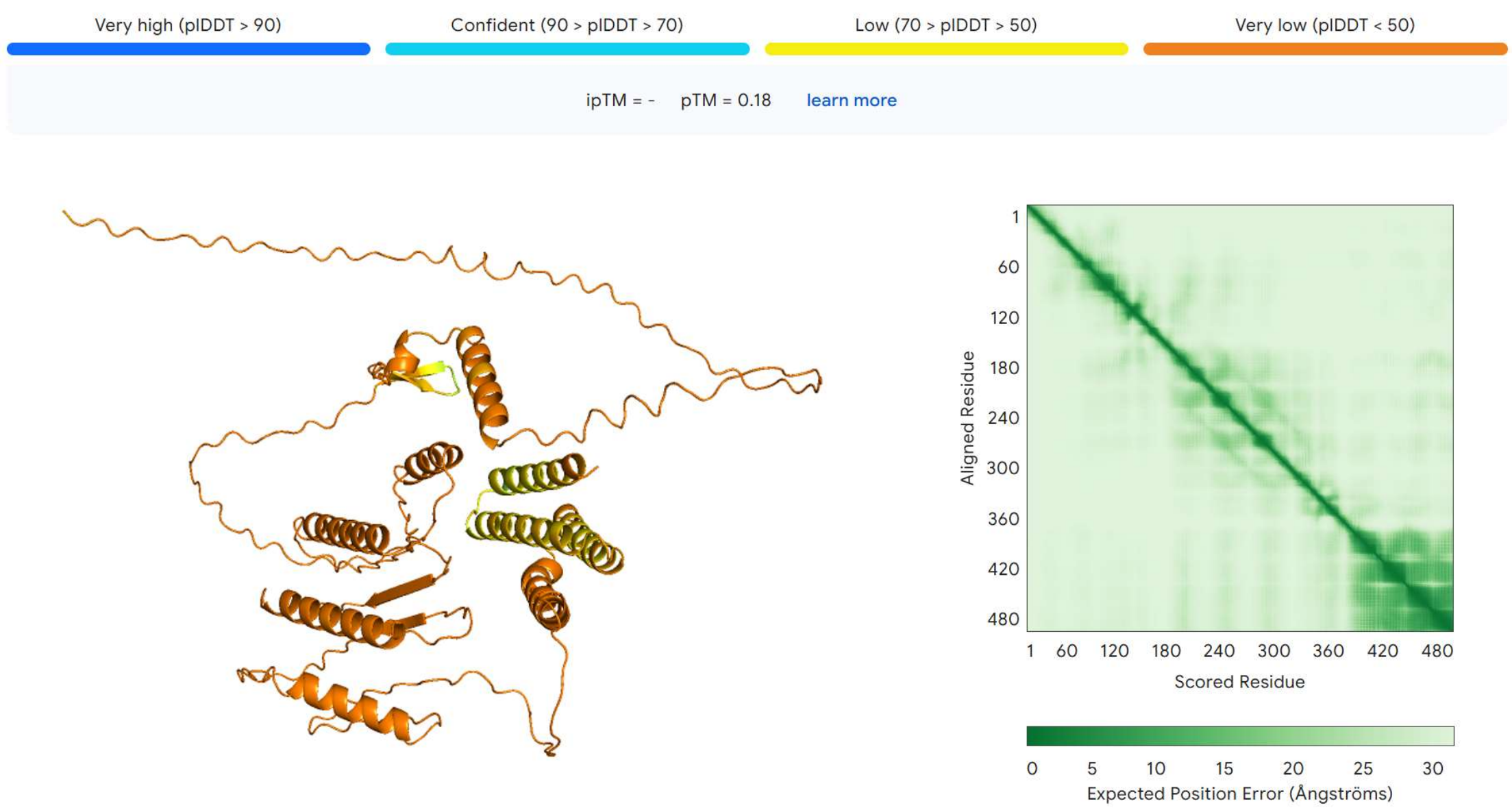

Supplementary Figure 8 Quality assessment of the protein structure predicted by AlphaFold v3. The upper colored bar indicates the predicted local distance difference test score (pIDDT), which evaluates model confidence per residue: very high confidence (pIDDT > 90) in dark blue; confident (90 > pIDDT > 70) in light blue; low (70 > pIDDT > 50) in yellow; and very low (pIDDT < 50) in orange. The predicted post-translational modification (iPTM) is shown as "-", and the predicted template modeling score (pTM) is 0.18. The lower line plot shows the predicted positional error for each residue, with higher values indicating lower reliability in that region. The x-axis represents the amino acid residue position (1-481). The highest ranking score for the model is 0.57.

Minor points (editorial/clarity)

1. Ensure all key controls are explicitly described in the Results text (not only Methods), especially for HR/cell death assays (negative/positive controls are stated in Methods).

Response: Thank you for your comments. We have revised the Results section to explicitly describe all key controls for the HR/cell death assays. Specifically, we have added the following statements:

(1) As expected, co-transformation of Sr50 and AvrSr50 (positive control) induced evident necrosis in wheat protoplasts, whereas no necrotic phenotype was observed in protoplasts transformed with the LUC alone (negative control) (Fig. 4g-h).

(2) No cell death was observed in the GFP, empty vector, or empty vector plus $LaCl_3$ control regions, whereas expression of Yr26 alone induced a clear cell death phenotype (these controls were previously described only in the Methods section) (Fig. 4j). The corresponding modifications have been made in the revised manuscript.

2. Evolutionary/orphan-gene claims: the text states no significant homology outside Triticeae and proposes recent genomic events. Please ensure thresholds/databases and search parameters are clearly stated (e.g., BLAST e-value cutoffs, proteome sets queried) and that claims match those criteria.

Response: Thank you for your comments. We have now clearly described the BLAST search parameters and databases in the Methods section. BLAST searches were performed using the following parameters: expectation value threshold of 0.05, word size of 16, maximum target sequences of 100, and match/mismatch scores set to the default linear gap costs. Low-complexity regions were filtered, and masking was applied for the lookup table only. The parameter for automatically adjusting parameters for short input sequences was enabled, and the maximum matches in a query range was set to 0 (no limit). All other parameters were kept at default values. The revised manuscript explicitly states these criteria, and the claim of “no significant homology outside *Triticeae*” is strictly based on these thresholds.

3. Figure quantification: where microscopy/staining is used, pair representative images with quantified summaries and explicit “n” (infection sites counted are mentioned).

Response: Thank you for your comments. Thank you for your valuable comment. Accordingly, we have revised all relevant figures and their legends. For each microscopy or staining experiment (including ROS accumulation and fungal biomass assays), we now provide quantitative summaries alongside the representative images. The number of infection sites counted (*n*) has been explicitly stated in the figure legends. These modifications ensure that all quantified data are accompanied by clear sample size information.

4. Reporting summary: the manuscript references a Nature Portfolio Reporting Summary. Please ensure it is complete and consistent with figure legends (replicates, randomization/blinding where applicable, data exclusions).

Response: Thank you for your comments. We have carefully reviewed and completed the Nature Portfolio Reporting Summary to ensure it is fully consistent with the figure legends. Specifically, we added a dedicated "Statistical analysis" subsection in the Reporting Summary, which includes:

a) Two-group comparisons were performed using the two-tailed Student's *t*-test function from the rstatix package (version 0.7.3).

b) For comparisons among three or more groups, one-way ANOVA was conducted via the aov function from the base R stats package, followed by post-hoc multiple comparisons using Tukey’s HSD test.

c) Significance levels were indicated as follows: $*P < 0.05$, $**P < 0.01$, $***P < 0.001$.

d) The software used for all analyses was R v4.2.0.

e) The definition of "*n*" for each experiment type (e.g., *n* = number of biologically independent plants; *n* = number of infection sites; *n* = number of independent experiments as specified in each figure legend).

In addition, we have verified that all information regarding randomization, blinding, and data exclusions (none) is accurately and consistently reported in both the Reporting

Summary and the relevant figure legends. The completed Reporting Summary has been uploaded along with the revised manuscript.

The genetic identification of Yr26/G4 is strong and potentially high-impact, and the domain-level functional mapping is compelling. However, for Nature Communications, the mechanistic framing around channel/Ca^{2+}-influx should either be substantiated with additional data or more carefully constrained as a hypothesis, and the resistance phenotyping/statistical reporting should be strengthened to match the journal's rigor expectations.

Response: Thank you for the constructive feedback. We are glad that the genetic identification and domain mapping are considered strong and compelling. In response to the mechanistic framing, we have added experimental data (Ca^{2+} influx, $LaCl_3$ blockade, subcellular localization) and explicitly labeled the channel-like mechanism as a hypothesis, avoiding overstatement. We have also strengthened phenotyping and statistical reporting with detailed sample sizes, statistical tests, and variance measures. We believe the revised manuscript now meets the rigor expected by Nature Communications.

Reviewer #3 (Remarks to the Author):

This manuscript reports the cloning and functional validation of the wheat stripe rust resistance locus *Yr26* (synonymous with *Yr24*/*YrCH42*/*YrG22*) and concludes that it encodes an atypical transmembrane "orphan" protein. The authors mapped the locus to a very small genetic interval, identified a single candidate transcript, and support causality using independent EMS loss-of-function mutants, BSMV-VIGS knockdown, CRISPR knockouts, and transgenic complementation into susceptible wheat. Using AlphaFold predictions, they identified several domains within the protein and propose that a C-terminal "four-helix beam" domain (Domain 3) is required for induction of cell death, based on cell-death assays in wheat protoplasts and *N. benthamiana* following expression of full-length *Yr26* or Domain 3. Finally, they analyzed natural variation and concluded that the functional resistant haplotype (*Yr26_h1*) is associated with a key

SNP, and that *Yr26* likely first appeared in cultivated emmer and was introgressed into bread wheat through breeding.

The cloning of the gene and the realization that it corresponds to several previously mapped resistance loci is important, and the discovery that it is unrelated to known classes of R genes is novel. The authors also convincingly show that cell-death activity maps to a C-terminal domain. However, the manuscript lacks mechanistic insight, and several issues need to be addressed and clarified, particularly regarding the induction of cell death in susceptible and non-host systems and the hypothesis that the gene first appeared in domesticated emmer.

Response: Thank you for your comments. Your recognition of the importance of cloning *Yr26* and the novelty of its structural divergence from known resistance gene classes is encouraging. We also thank you for identifying the key conceptual issues that needed to be addressed—particularly the apparent paradox between *Yr26*-triggered cell death in heterologous systems and its race-specific resistance, as well as the rigor of our evolutionary origin conclusions.

These comments have prompted us to substantially strengthen the mechanistic framework and revise our evolutionary interpretations. The major revisions we have made in response to your concerns include:

(1) Resolving the cell death vs. race-specificity paradox (Comments 1, 2, 9, 11)

We have added extensive new data that together support a revised mechanistic model. First, we screened 166 *Pst* isolates collected from Chinese fields during 2023–2025, identifying 26 that are virulent on *Yr26*, and confirmed that *Yr26* confers resistance only to avirulent Pst isolates, not to leaf rust or powdery mildew (Fig. 4a–c). Second, and critically, we discovered and corrected an error in our previous expression data: repeated experiments now consistently show that *Yr26* is transcriptionally induced by both avirulent and virulent Pst isolates, but not by leaf rust or powdery mildew (Fig. 4d). This finding fundamentally revises our understanding: race specificity is not determined at the level of transcriptional induction, but likely involves post-

translational mechanisms or pathogen-encoded suppressors that block HR execution in compatible interactions. Third, we demonstrate that *Yr26*-mediated immunity requires Ca^{2+} influx (Fig. 4i) and that the Ca^{2+} channel blocker $LaCl_3$ abolishes *Yr26*-induced cell death (Fig. 4j). These data collectively support a model in which *Yr26* functions as a *Pst*-specific, Ca^{2+}-dependent cell death executor, whose activity can be suppressed by factors specifically present in virulent races.

(2) Strengthening the evolutionary origin analysis (Comment 5)

We fully agree that our original conclusion—that *Yr26* "first emerged in domesticated emmer"—overstated the evidence and conflated gene origin with the origin of the functional resistance allele. To address this, we expanded our wild emmer (WEW) screening from 33 to 359 accessions, identifying five new WEW accessions carrying the *YR26* locus. These accessions harbor a previously unidentified non-functional haplotype, *Yr26_h6*, which contains additional SNPs and a 289-bp insertion relative to the functional Yr26_h1 allele (Supplementary Fig. 13). The presence of *Yr26* in wild emmer, together with its homologs in rye (diverged ~5.94 MYA), clearly indicates that the gene itself predates domestication. Accordingly, we have revised our conclusion to state that the functional resistance allele likely arose in cultivated emmer or an unsampled wild emmer lineage, rather than that the gene itself originated at this stage.

(3) Providing subcellular localization and protein-level validation (Comments 3, 4)

We have added subcellular localization data showing that *Yr26* (using the M15 S441N mutant, which retains partial function) localizes to the plasma membrane, with Domain 3 being necessary and sufficient for membrane localization (Supplementary Fig. 9). We also performed Western blot analysis confirming that full-length *Yr26* and all truncated/mutated variants used in cell death assays are stably expressed in *N. benthamiana*, ruling out the possibility that differences in cell death activity arise from differences in protein accumulation.

We believe these substantial additions—spanning expanded pathogen screening, corrected expression data, Ca^{2+} signaling assays, subcellular localization, protein

biochemical validation, and expanded evolutionary sampling—collectively address your concerns regarding mechanistic insight and evolutionary rigor. The revised manuscript now presents a more nuanced and data-supported model of Yr26 function and a more carefully qualified evolutionary narrative. We are grateful for your insightful critique, which has significantly improved the depth and accuracy of our study.

Main comments

1. Lack of sufficient mechanistic insight. The nature of resistance conferred by Yr26 remains unclear. The gene is associated with race-specific resistance, however expression of the gene in susceptible wheat as well as in tobacco leads to cell death. The authors argue that, unlike classical R genes, Yr26 does not interact with an effector, but rather operates as a protein whose activation or abundance is sufficient to trigger cell death, for example by activating downstream processes that culminate in cell death. According to this scenario, one would expect broad-spectrum resistance, potentially even resistance to multiple diseases, as reported for several non-canonical resistance genes. However, the resistance conferred by Yr26 is race-specific; The authors explain race specificity by proposing that Yr26 functions as a downstream executor of cell death whose transcription is selectively induced during incompatible interactions. If, as suggested, resistance is determined by gene activation, one would need to assume the existence of a fungal factor specific to avirulent races that selectively activates Yr26 expression. Such a factor has not been reported and would represent a novel mechanism of specificity. However, no race-specific effector, upstream receptor, or regulatory mechanism controlling Yr26 induction has been identified or proposed. Thus, while the proposed model is plausible, race specificity is inferred rather than mechanistically demonstrated.

Response: Thank you for your insightful and constructive comments. We greatly appreciate your deep thinking on the mechanistic of *Yr26*-mediated resistance. The

points raised are excellent and represent the central focus of our ongoing and future work. We have now added substantial new data in the revised manuscript to address these concerns, as detailed below.

To further clarify the race-specificity of *Yr26*, we screened 166 *Pst* isolates that were virulent to Avs collected from Chinese fields during 2023–2025 (Supplementary Table 5). Among them, 26 isolates were virulent to the near-isogenic lines Avs+*Yr24*/*Yr26*, confirming the natural occurrence of virulent isolates to *Yr26*. Moreover, susceptible and resistant isolates span a wide range of latitudes and longitudes with CYR32 and CYR34 were chosen to inoculate the transgene line T_3-17-5 (Fielder+*Yr26*) and wild type accession Fielder. These races/isolates displayed distinct virulence patterns to a set of *Yr* genes (Supplementary Table 1). Fielder is susceptible to all ten races/isolates. T_3-17-5 is resistant to the avirulent isolates and susceptible to the virulent isolates, further indicating that *Yr26* is effective to specific races/isolates (Fig. 4a). In addition, a leaf rust (*Puccinia triticina*, *Pt*) race THTT (Fig. 4b) and a powdery mildew (*Blumeria graminis* f. sp. *tritici*, *Bgt*) race E09 (Fig. 4c) were used to inoculate T_3-17-5 and Fielder and both of them exhibited susceptible responses, indicating that the *Yr26* does not confer broad-spectrum resistance to multiple pathogens

Expression analysis revealed that *Yr26* was induced by all ten tested *Pst* isolates, but not by THTT (*Pt*) or E09 (*Bgt*) (Fig. 4d), indicating that Yr26 expression is specifically induced by *Pst* and suggest the existence of a *Pst*-derived factor responsible for this induction.

Although expression of *Yr26* alone can induce cell death in wheat protoplasts and tobacco leaves, we observed no HR in T_3-17-5 leaves following infection with CYR34 and four independent virulent isolates (Fig. 4a). This finding indicates that Yr26-induced HR can be suppressed by specific *Pst* factors, suggesting that additional fungal components are involved in modulating the resistance response.

Notably, the current result showing CYR34-induced *Yr26* expression is contrary to our previous finding. We therefore repeated the CYR34 inoculation experiment twice and

consistently observed that CYR34 does induce *Yr26* expression. We re-evaluated our earlier data and concluded that the previous negative result was likely due to compromised inoculum vitality. This correction is critical, as it clarifies that *Yr26* expression can be induced by both avirulent and virulent isolates, and that race specificity is determined not merely by transcriptional activation but likely by additional post-transcriptional or post-translational mechanisms, or by the presence of suppressive factors in virulent isolates.

We thank you again for these comments, as they have prompted us to re-examine our work and gain a clearer understanding of the *Yr26*–*Pst* interaction. The correction regarding *Yr26* expression in compatible isolates is particularly important for future mechanistic studies. Accordingly, we have revised the manuscript.

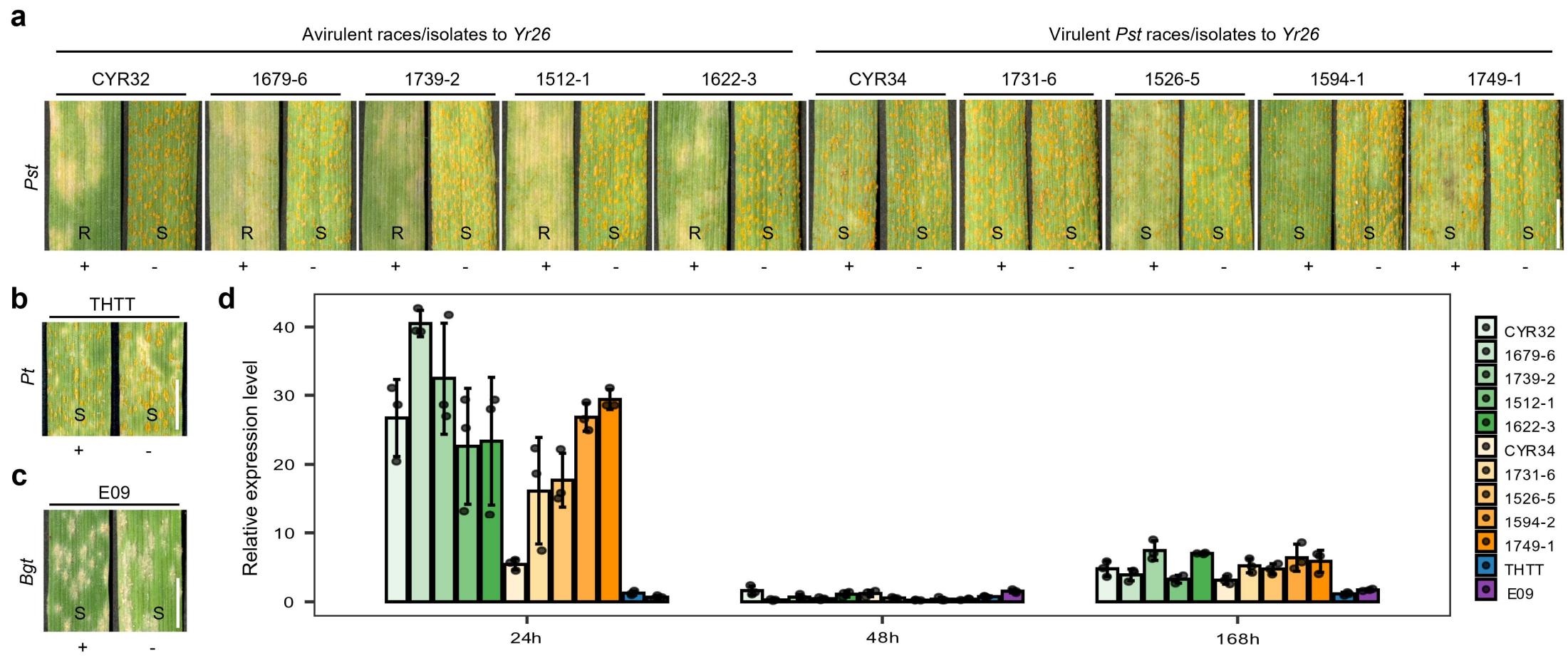

Fig.4 a-d *Yr26* exhibits race-specific resistance and is specifically induced by *Pst* infection.

a-c. Responses of the transgenic line T$_3$-17-5 (Fielder + *Yr26*) and the wild-type (WT) cultivar Fielder to different pathogens. (a) Ten diverse *Pst* races/isolates; (b) Leaf rust (*Puccinia triticina*, *Pt*) race THTT; and (c) powdery mildew (*Blumeria graminis* f. sp. *tritici*, *Bgt*) race E09. "+" and "–" indicate T$_3$-17-5 (carrying *Yr26*) and Fielder (lacking *Yr26*), respectively. R, resistant; S, susceptible. Scale bar, 0.5 cm.

d. Transcript levels of *Yr26* detected by real time quantitative reverse transcription (qRT-PCR) during 0-168 hpi in response to infection by virulent and avirulent *Pst* race CYR32 and CYR34 isolates. Mock inoculation was used as control. Samples inoculated with CYR32 at 0 hpi were used as calibrations to calculate the relative quantification (RQ) values using the delta-delta method with RQ = $2^{-\Delta\Delta Cq}$. The *TaEF-1α* was used as a normalizer. Data are shown as mean ± s.d. (n = 3 biological replicates). Each replicate is shown as a single data point. Green, avirulent *Pst* races/isolates; Orange, virulent *Pst* races/isolates; Blue, leaf rust race THTT; Purple, powdery mildew race E09.

2. Given that this is a central issue, it is essential to conduct a broader screen of pathogen races and report the resistance spectrum to additional stripe rust races, as well as to stem and leaf rusts, and possibly to other wheat pathogens.

Response: Thank you for your suggestion. we screened a total of 166 *Pst* isolates, as well as a leaf rust race (THTT) and a powdery mildew race (E09), on the Yr26 transgenic line T_3-17-5 and the wild-type accession Fielder. Fielder was susceptible to all tested pathogens. Due to the unavailability of stem rust isolates in our laboratory, we were unable to perform stem rust inoculation in this study. T_3-17-5 was susceptible to 26 out of the 166 *Pst* isolates (Supplementary Table 5), as well as to THTT and E09 (Fig. 4b-c), while it remained resistant to the remaining 140 *Pst* isolates. These results confirm the race-specificity of Yr26-mediated resistance.

3. Subcellular localization. Subcellular localization of Yr26 (and Domain 3) is not demonstrated in planta, which is critical for interpreting any proposed "channel-like" or membrane-associated function. The Discussion speculates about an ion-channel analogy (e.g. WTS), but this hypothesis is not experimentally tested. Such assays could be performed in *N. benthamiana*, since the protein induces cell death in this system.

Response: Thank you for this important comment and suggestion. We fully agree that demonstrating the subcellular localization of *Yr26* is critical for interpreting its proposed membrane associated function. We attempted to determine the subcellular localization of Yr26 via transient expression in wheat protoplasts and *N. benthamiana* leaves. However, multiple attempts at protein extraction and localization failed, likely due to the low expression level of *Yr26* or its hypersensitivity to necrosis. As an alternative approach, we took advantage of the M15 mutant (S441N), which carries a point mutation that compromises resistance but does not completely abolish the cell death function, suggesting that the M15 protein may retain normal biochemical activity (Fig. 4g-h). We therefore used the M15 sequence for subcellular localization analysis. The results showed that M15:Yr26^{S441N} and M15:Domain3^{S441N} localized to the plasma membrane, with TAWPI6-mCherry serving as a membrane marker (Koike et al. 2005). In contrast, Domain1+Domain2 (lacking Domain3) exhibited both nuclear and

cytoplasmic membrane localization (Supplementary Fig. 9). These findings provide further evidence that Yr26 functions as a transmembrane protein.

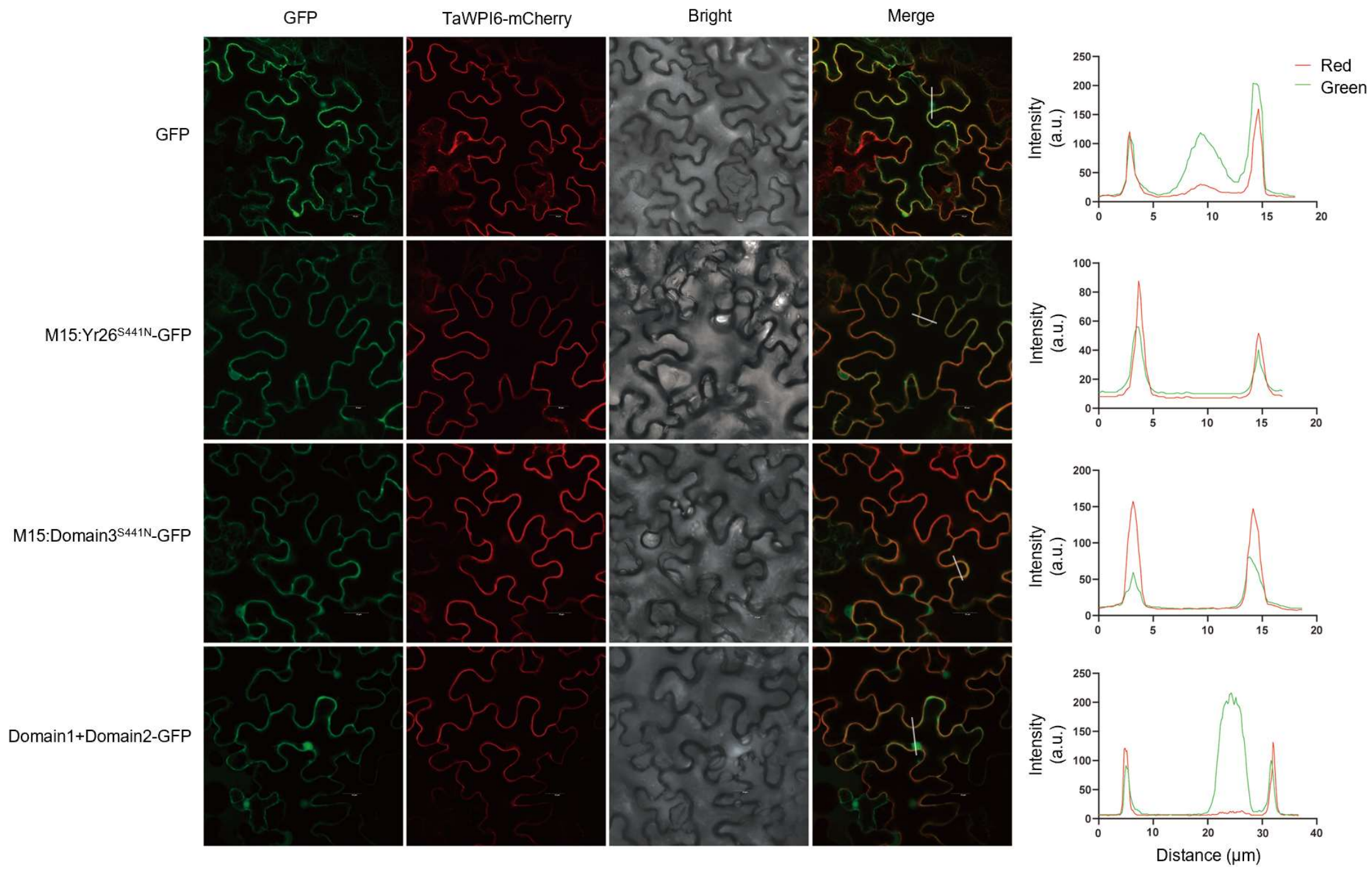

Supplementary Figure 9 Subcellular localization of Yr26 and its predicted domains in *N. benthamiana* epidermal cells. C-terminal GFP fusion proteins were generated using the pYJ-GFP-Nos vector and were transiently expressed in *N. benthamiana* leaves. TaWPI6-mCherry were co-infiltrated served as a cytoplasmic membrane localization marker. GFP (green) and mCherry (red) fluorescence signals were observed, along with bright-field (Bright) and merged (Merge) images. The fluorescence intensity profiles (right) quantify the relative fluorescence intensity (a.u.) along the white lines shown in the merge images, with distance measured in micrometers (μm). For each treatment, at least three independent cells ($n > 3$) were examined.

Reference:

Koike, M. et al. Molecular characterization of a cold-induced plasma membrane protein gene from wheat. *Molecular Genetics and Genomics* **274**, 445-453 (2005).

4. Protoplast assays. The protoplast cell-death assay uses luciferase as a proxy for viability, which is reasonable. However, for a transmembrane protein it would be important to additionally show expression-level normalization (protein accumulation), determine whether cell death correlates with membrane localization, and test whether Domain 3–induced cell death is abolished by mutations that disrupt predicted transmembrane helices (structure–function analysis).

Response: Thank you for your suggestion. We addressed the requirement of the

predicted transmembrane architecture for *Yr26* function using complementary genetic and biochemical approaches, which together provide robust structure–function evidence.

Deliberate truncation of the TM3 helix abolishes cell death activity. Domain disruption experiments in both wheat protoplasts and *N. benthamiana* leaves demonstrated that removing the TM3 helix eliminates cell death induction by both full-length Yr26 and Domain 3 (Fig. 4g–h; Supplementary Fig. 10b). This directly establishes that an intact TM3 helix is required for Yr26-mediated cell death.

Naturally occurring loss-of-function mutations independently confirm the structural requirement. Among the six EMS-derived susceptible mutants, Mut6 carries a premature stop codon that truncates 27 amino acids at the C-terminus, removing a portion of the TM3 region and completely abolishing resistance (Fig. 2f). Two additional missense mutations within Domain 3—G416R (Mut5/M65) and S441N (M15)—also eliminate or significantly weaken cell death induction (Fig. 4g–h). These independent mutations, recovered from unbiased forward genetic screens, converge on the same structural region, providing strong genetic validation that the integrity of Domain 3, and particularly its TM helices, is essential for *Yr26* function.

Protein expression normalization confirms functional differences are not attributable to differential accumulation. Western blot analysis verified that full-length *Yr26*, Domain 3, and all truncated/mutant variants were expressed as detectable proteins in *N. benthamiana* leaves (Supplementary Fig. 10c). Notably, full-length Yr26 and Domain 3 accumulated to markedly lower levels than other variants under equal sampling conditions—likely because these variants rapidly trigger cell death, leading to protein degradation. Despite their lower abundance, they induced the strongest cell death, confirming that functional differences reflect intrinsic structural properties rather than differential expression.

Collectively, the convergence of deliberate domain truncation and independent loss-of-

function mutations on Domain 3 establishes a clear causal relationship: disruption of the predicted transmembrane architecture abolishes *Yr26*-mediated cell death.

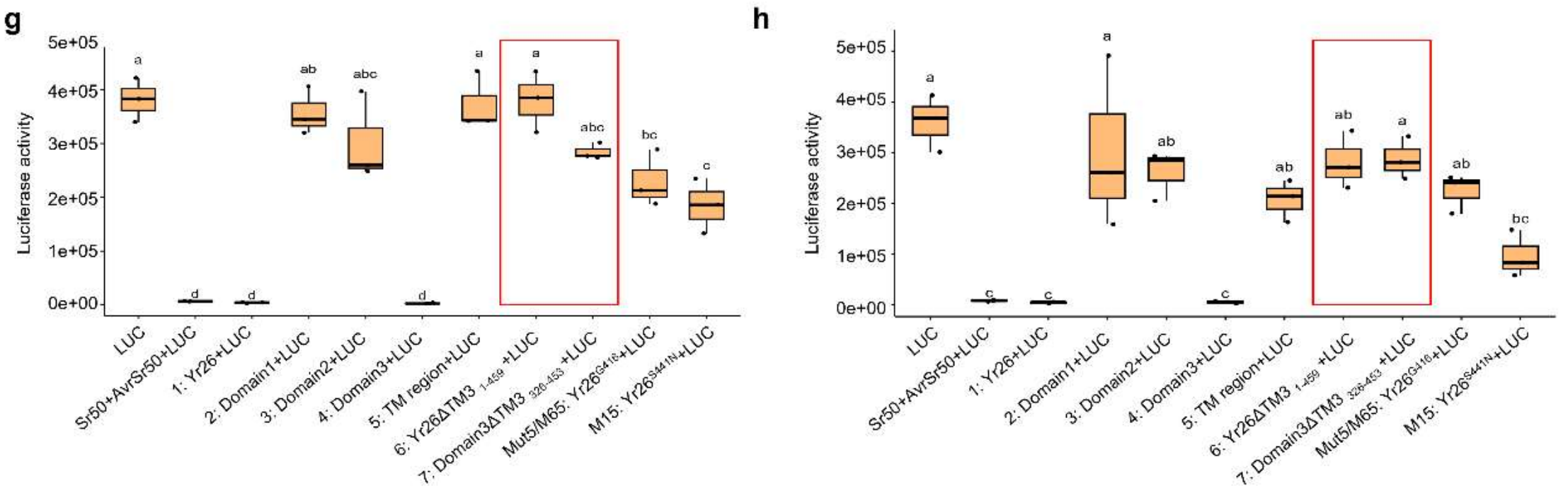

Fig. 4g-h. Luciferase activity of wheat protoplasts 16 h after co-transformation with indicated constructs and a construct carrying a luciferase reporter gene. (g) Resistant line 92R137; (h) susceptible line Fielder. Co-transformation of wheat stem rust resistance gene *Sr50* and corresponding avirulence gene *AvrSr50* known to induce necrosis in wheat protoplasts was used as the control. Luciferase activity values were measured from three biological replicates to assess cell viability. Error bars represent standard deviations of three biological replicates. Significance of differences were analyzed by one-way ANOVA. A *P*-value > 0.05 was considered not significant.

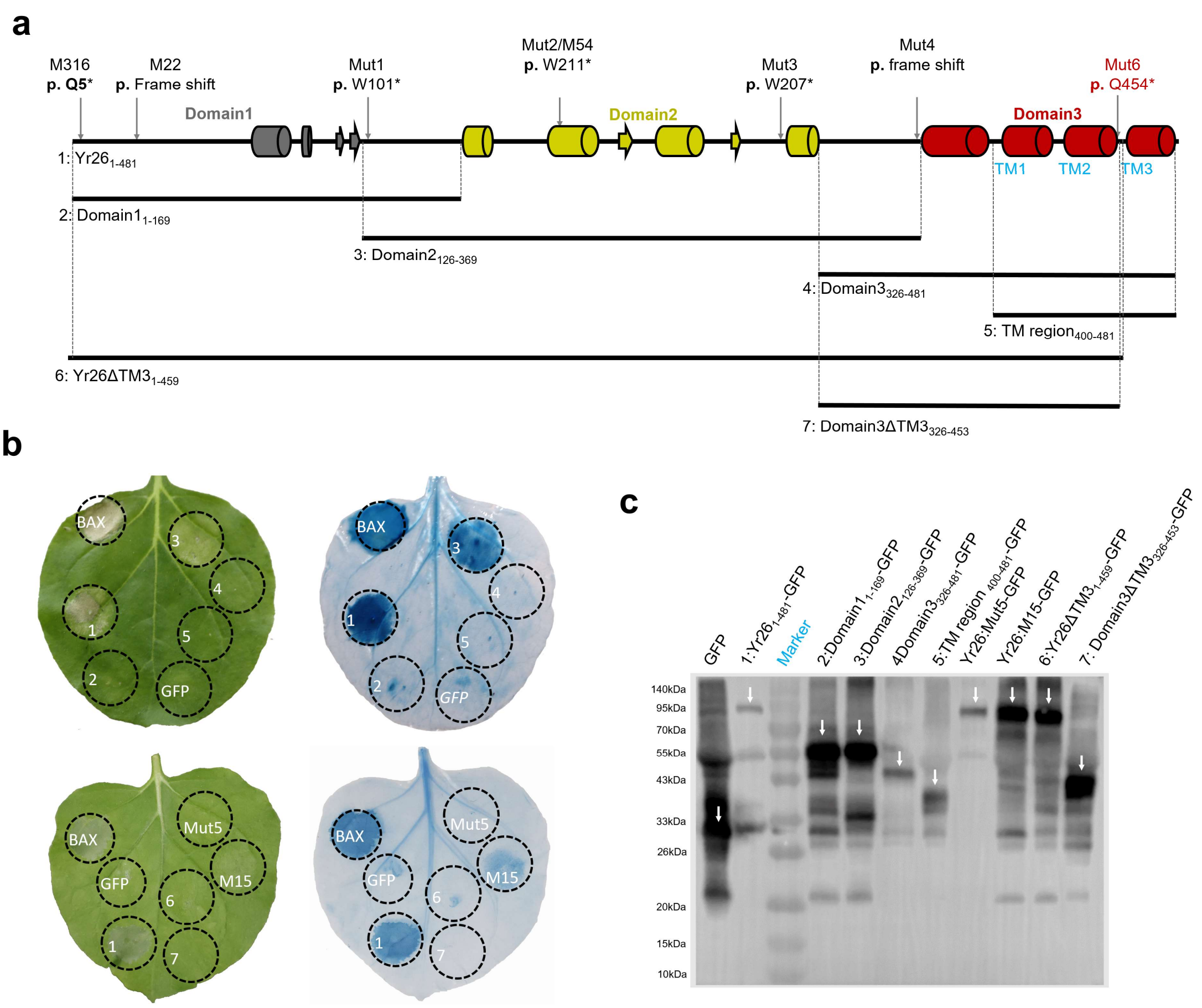

Supplementary Figure 10 *Yr26* induces cell death in *N. benthamiana*.

a. Secondary structure of *Yr26*. Cylinders, α-helices; arrows, β-sheets. Protein domains are illustrated by colors described above. Arrows indicate the position of the six loss-of-function mutations generated by EMS. Black bars and "Δ" represent constructs used to transform wheat protoplasts and remove the corresponding domain, respectively.

b. Transient expression of *Yr26* and its truncated structures (C-terminal 3×Flag-tagged) induced cell death in *N. benthamiana* under a Cub promoter at 48 h post inoculation. *Bax* (inducing cell death) and *GFP* were used as controls. Dead cells were identified by trypan blue staining. Similar results were obtained from three independent biological replicates (n = 3). Overexpression of full-length Yr26 or Domain 3 alone induced cell death, whereas deletion of the TM3 helix or introduction of Domain 3 point mutations (G416R, S441N) abolished or severely reduced this activity, indicating that an intact Domain 3 structure is essential for Yr26-mediated HR and pathogen containment.

c. Protein expression analysis of *Yr26* and its truncated/mutant variants as GFP fusion proteins. Protein extracts were separated by SDS-PAGE and detected by anti-GFP antibody. GFP alone used as control. The molecular weight of marker is shown on the left. White arrows indicate the expected bands of the fusion proteins.

5. Evolutionary origin of *Yr26*. Phylogenetic analysis indicates that lineages L1/L2 (including 92R137) are absent in wild emmer but present in cultivated emmer and in descendant lineages including durum and bread wheat. Based on this, the authors conclude that the gene “first emerged in domesticated emmer and was inherited into durum”. Several factors could affect the accuracy of this conclusion, including sampling bias and difficulties in presence/absence calling from short-read data, which is particularly problematic for orphan genes. More importantly, the presence of homologs in rye (which diverged from wheat more than 5 MYA) does not align with the hypothesis that the gene itself first appeared in cultivated emmer. A more appropriate interpretation of the data would be that the functional resistant allele likely arose in domesticated emmer (or in an unsampled wild lineage) and was subsequently selected and introgressed, rather than that the gene itself “appeared” at this stage. This interpretation is also more consistent with the authors’ own suggestion of precursor or non-functional alleles and a multistep evolutionary model. I would also recommend more robust validation of wild emmer “absence,” such as coverage-based thresholds, assembly-based confirmation in multiple WEW genomes, or targeted PCR across a broader WEW collection. Finally, if the authors want to propose that the gene originated in domesticated emmer, the mechanism underlying such gene emergence should be explicitly addressed.

Response: Thank you for your thoughtful and constructive comments. We agree that sampling bias and presence/absence calling from short-read data can affect the accuracy of evolutionary conclusions, particularly for orphan genes. To more rigorously investigate the origin of *Yr26*, we expanded our screening to include 359 wild emmer (WEW) accessions kindly provided by Prof. Lin Huang at SAU. Based on the *DM2* functional marker, we identified five new accessions carrying the *YR26* locus. Haplotype analysis revealed a novel haplotype in these accessions, designated *Yr26_h6* (Supplementary Table 4). Compared with the functional allele *Yr26_h1*, *Yr26_h6* contains, in addition to SNP1-4 and the 12-bp indel, five additional SNPs and a 289 bp insertion (Big indel1) (Supplementary Fig. 13). All five accessions carrying *Yr26_h6* are susceptible to both CYR32 and CYR34 (Supplementary Table 4), indicating that this haplotype does not confer resistance. These findings identify a non-functional *Yr26* haplotype in wild emmer and provide new evidence regarding the evolutionary origin of *Yr26*, suggesting that the functional *Yr26* allele may have evolved before domestication. We have incorporated these new data into the revised manuscript and have revised the conclusion to state that functional *Yr26* allele originated from cultivated emmer or unsampled wild emmer accessions.

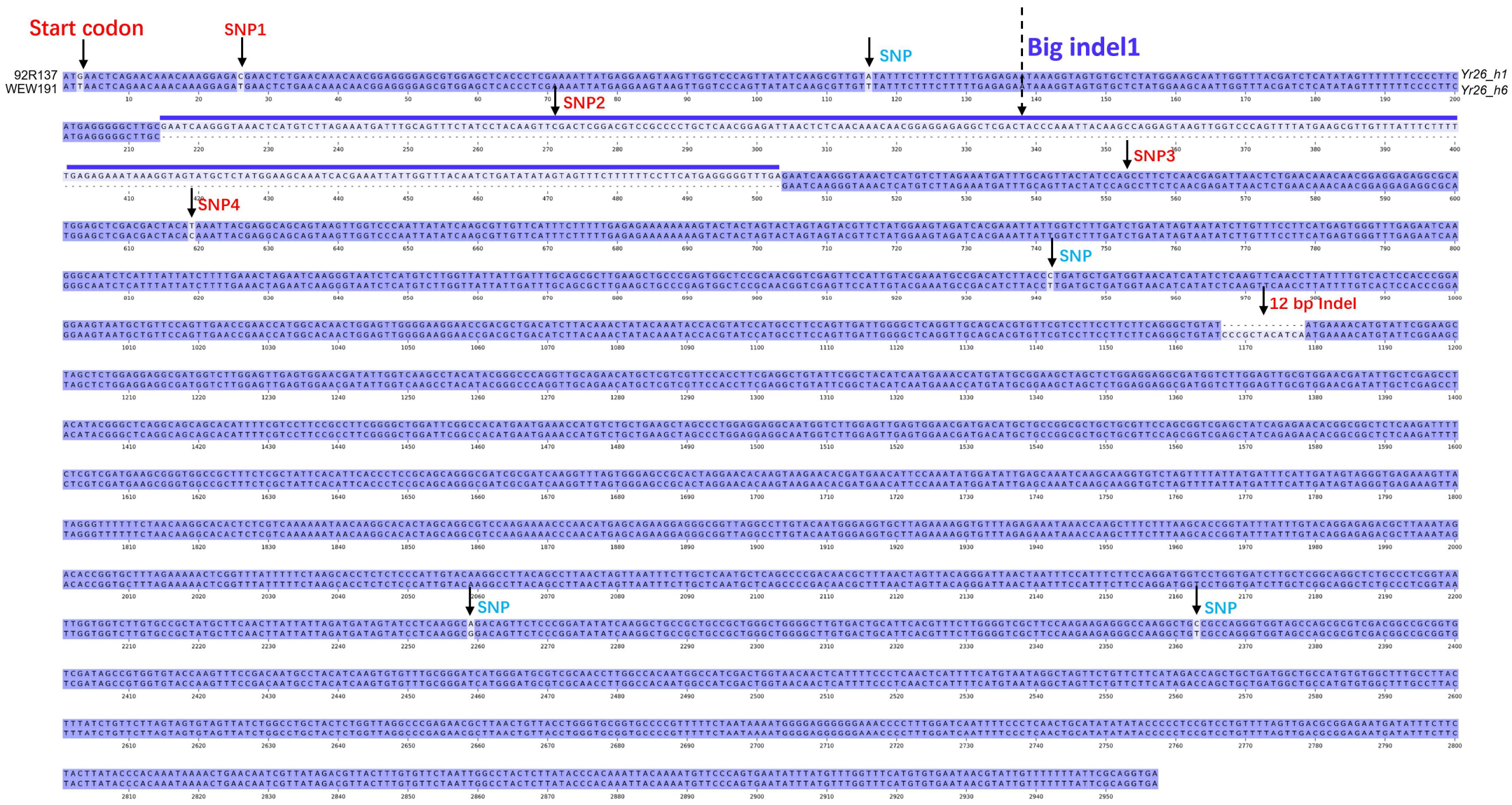

Supplementary Figure 13 Alignment of *Yr26_h1* in 92R137 and *Yr26_h6* in WEW191. Blue bar, Indel1. SNPs in haplotypes *Yr26_h1* – *h5* are marked by black arrows with red font. In contrast, SNPs unique to the *Yr26_h1* and *Yr26_h6* comparison are indicated by black arrows with blue font.

Additional comments

6. Line 106: How was this isolate selected?

Response: Thank you for your comment. CYR32 and CYR34 are both prevalent *Pst* races in China, each with a frequency exceeding 10% in major wheat-growing regions. CYR32 is avirulent to *Yr26* and has been widely used in genetic mapping studies for *Yr26* (Zhang et al. 2013; Wu et al. 2018). CYR34, in contrast, is was the first identified race virulent to *Yr26* (designated V26), and it has been responsible for the breakdown of *Yr26*-mediated resistance in China since approximately 2011 (Han et al. 2015). Accordingly, we have revised the sentence in the manuscript (Line 106) as "To characterize *Yr26*-mediated resistance, seedlings of lines 92R137 (+*Yr26*), Yangmai158 (YM158, -*Yr26*), and a pair near-isogenic lines NIL-R (+*Yr26*)/NIL-S (-*Yr26*) were inoculated with *Pst* race CYR32 that is avirulent to *Yr26* (Supplementary Table 1)." Detailed information regarding CYR32 and CYR34 has also been added to Methods the revised manuscript.

Reference:

Han, D. et al. Emerging *Yr26*-virulent races of *Puccinia striiformis* f. *tritici* are threatening wheat production in the Sichuan basin, China. *Plant Disease* **99**, 754-760 (2015).

Wu, J. et al. SNP-based pool genotyping and haplotype analysis accelerate fine-mapping of the wheat genomic region containing stripe rust resistance gene *Yr26*. *Theoretical and Applied Genetics* **131**, 1481-1496 (2018).

Zhang, X. et al. Fine mapping of wheat stripe rust resistance gene *Yr26* based on collinearity of wheat with *Brachypodium distachyon* and rice. *PloS one* **8**, e57885 (2013).

7. Line 117: The text does not align with the DAB results shown in Figure 1. The results at 48 hpi show strong DAB staining in the resistant interaction, suggesting an early response. However, the text states that the results indicate that "Yr26 inhibited post-infection fungal development". A time-course analysis of ROS accumulation starting at 12hpi should be conducted, and the text revised accordingly. Additionally, quantitative measurement of ROS would be more appropriate than reliance on DAB staining alone. The authors may also consider PI staining to determine the onset of cell death.

Response: Thank you for your insightful comment. We agree that a time-course analysis of reactive oxygen species (ROS) accumulation is necessary to accurately interpret the

timing of the resistance response, and that quantitative measurements would strengthen the conclusions. Accordingly, we have conducted additional histological analyses and quantified key metrics during the *Pst* infection process in NIL-R and NIL-S.

Specifically, we performed time-course diaminobenzidine (DAB) staining to monitor ROS accumulation from 12 hpi to 72 hpi. Strong ROS accumulation was observed at infection sites in NIL-R compared to NIL-S at 48 hpi and 72 hpi, whereas no significant difference was detected at 12 hpi and 24 hpi (Fig. 1b-c). These results indicate that ROS burst occurs as a relatively early but not immediate response following infection.

In addition, we quantified *Pst* fungal biomass in wheat leaf tissues. The results showed comparable fungal development between NIL-R and NIL-S during early infection (1–4 dpi) (Fig. 1d-e). However, at 5–7 dpi, NIL-R exhibited a markedly reduced fungal biomass compared to NIL-S (Fig. 1c). These quantitative data, including ROS accumulation dynamics and fungal biomass measurements, provide robust evidence supporting that *Yr26* inhibits post-infection fungal development during the late infection stage (5–7 dpi), following an early ROS burst at 48–72 hpi.

Regarding the suggestion of PI staining to determine the onset of cell death, we attempted this experiment but were unable to obtain reliable results our experimental conditions, likely due to suboptimal staining conditions in wheat leaf tissues. Therefore, this study focused on ROS accumulation and fungal biomass quantification as functional readouts of resistance.

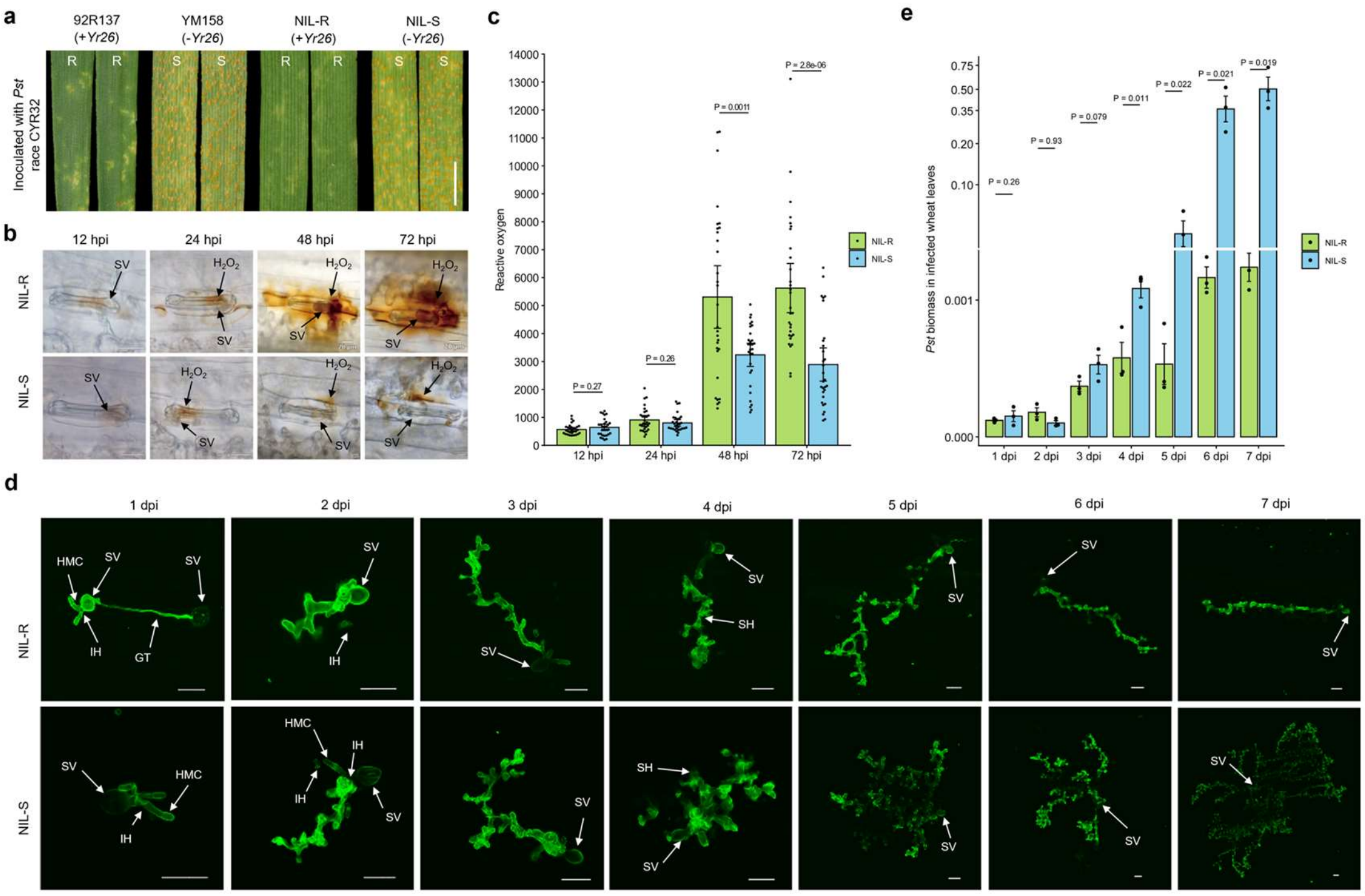

Fig. 1 Resistance response of *Yr26* to avirulent *Puccinia striiformis* f. sp. *tritici* (*Pst*) race CYR32

a. Wheat lines 92R137 (+*Yr26*), YM158 (-*Yr26*), and the near-isogenic line pair NIL-R: +*Yr26*; NIL-S: -*Yr26* inoculated with avirulent *Pst* race CYR32 at 14 days post inoculation (dpi). R: resistant, S: susceptible. Scale bar, 1 cm.

b-c. Histology and statistic of reactive oxygen production in *Yr26* near-isogenic lines inoculated with CYR32 at 12 hpi, 24 hpi, 48 hpi and 72 hpi. SV: substomatal vesicle. Data are shown as mean ± s.d. (n = 30 independent infection sites). Each infection site is shown as a single data point. Statistical significance was determined using two-tailed Student's t-tests and a P value > 0.05 was considered not significant.

d. Histology and cytology of fungal development during incompatible (resistant) and compatible (susceptible) interactions at 1-7 dpi. S, urediniospore; SV: substomatal vesicle; GT, germ tube; IH, infection hypha; HMC, haustorial mother cell; H, haustoria. $n > 30$ independent infection sites. Scale bars, 40 μm.

e. Biomass of *Pst* race CYR32 in infected leaves of *Yr26* near-isogenic lines. Data are shown as mean ± s.d. ($n = 3$ biological replicates). Each replicate is shown as a single data point. Statistical significance was determined using two-tailed Student's t-tests and a $P > 0.05$ was considered not significant.

8. Line 231: Please specify that the cultivar used for the protoplast assay was cv. Fielder, which is susceptible. It would have been informative to perform the same assay in a resistant line as well.

Response: Thank you for your comment and suggestion. We repeated the same protoplast assay in the resistant line 92R137 (carrying *Yr26*). The results were highly

consistent with those obtained in Fielder (Fig. 4g-h), indicating that the Yr26-mediated immune response is conserved across different wheat genotypes and is not dependent on the genetic background. We have specified the cultivars (92R137 and Fielder) used for the protoplast assay and have added these results to the revised manuscript.

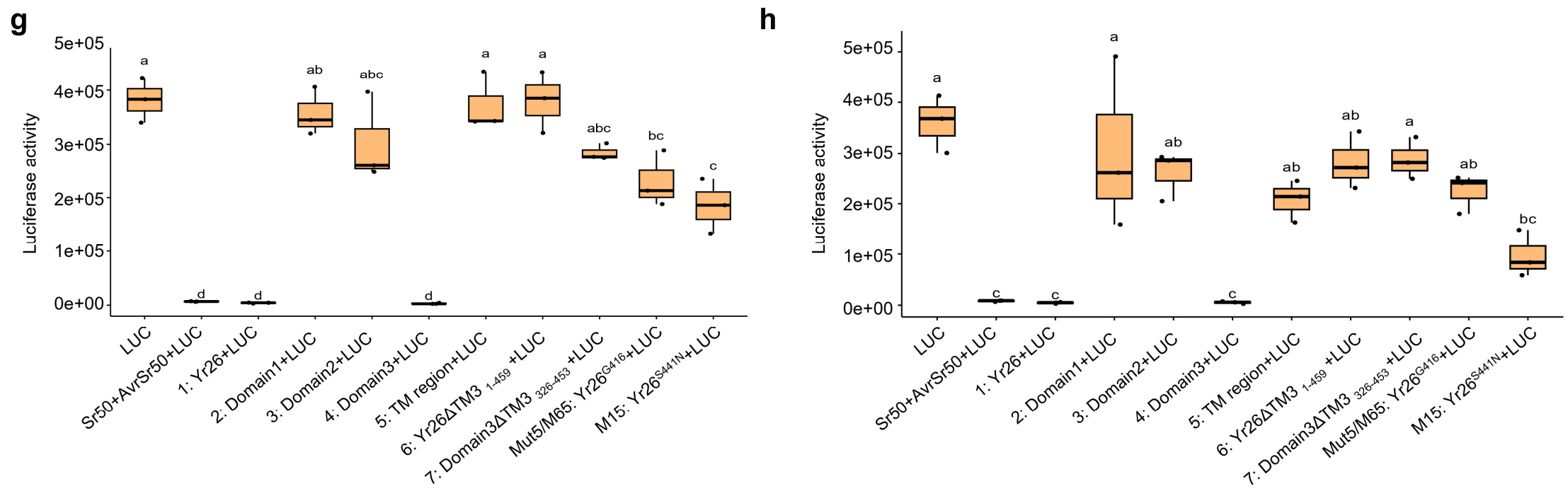

Fig. 4g-h. Luciferase activity of wheat protoplasts 16 h after co-transformation with indicated constructs and a construct carrying a luciferase reporter gene. (g) Resistant line 92R137; (h) susceptible line Fielder. Co-transformation of wheat stem rust resistance gene *Sr50* and corresponding avirulence gene *AvrSr50* known to induce necrosis in wheat protoplasts was used as the control. Luciferase activity values were measured from three biological replicates (n = 3) to assess cell viability. Error bars represent s.d. of three biological replicates. Significance of differences was analyzed by one-way ANOVA followed by Tukey's multiple comparisons test. $P >$ 0.05 was considered not significant.

9. Lines 257–269: Only two races were tested-CYR32 (avirulent) and CYR34 (virulent). This is insufficient to draw general conclusions about race specificity or to determine whether CYR34 virulence reflects effector loss, suppression, or bypass. Additional races should be tested.

Response: Thank you for your constructive comment. We have additionally performed virulence assays against a panel of 166 *Pst* races, together with resistance evaluations against leaf rust and powdery mildew, as well as expression analysis of *Yr26* in response to different elicitors (detailed in our response to Comment 1). The results revealed that *Yr26* expression is specifically induced by Pst regardless of race virulence and suggests a conserved inducing factor in *Pst*. Furthermore, Yr26 overexpression induced necrosis in *N. benthamiana* and wheat protoplasts, but virulent races failed to induce necrosis, implying they may encode suppressors of Yr26-mediated immunity.

10. Lines 343–345: The sentence referring to "trigger cell death in intact

transmembrane domains” is unclear. The data are insufficient to support the claim that these domains “are strictly upregulated during infection leading to hypersensitive responses.”

Response: We thank the reviewer for pointing out the unclear wording. To address this, we performed additional domain disruption experiments in both wheat protoplasts and *N. benthamiana* leaves. The results showed that disruption of the C-terminal TM3 helice eliminates *Yr26*-mediated cell death (Fig. 4g-h; Supplementary Fig. 10b). Accordingly, we revised conclusion to “Overexpression of full-length *Yr26* or the isolated intact Domain 3 region triggered cell death in wheat protoplasts and *Nicotiana benthamiana* leaves, whereas deletion or disruption of the C-terminal TM3 helices abolished this activity. These results establish Domain 3 as the critical functional module governing Yr26-mediated HR” in the re-submitted manuscript.

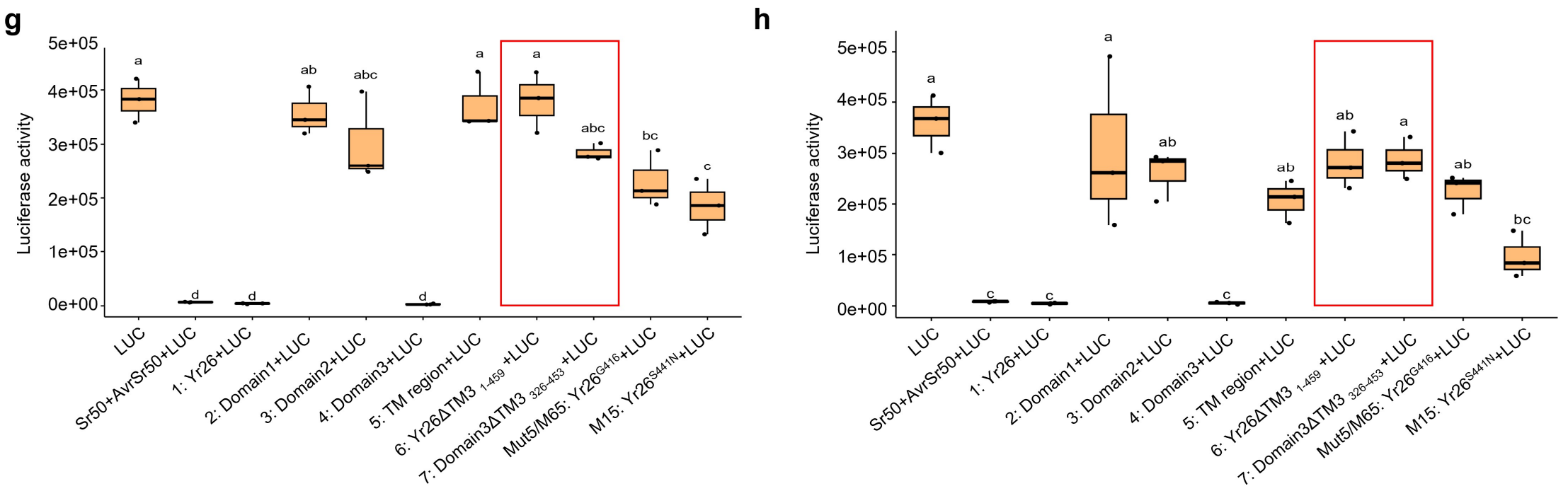

Fig. 4g-h. Luciferase activity of wheat protoplasts 16 h after co-transformation with indicated constructs and a construct carrying a luciferase reporter gene. (g) Resistant line 92R137; (h) susceptible line Fielder. Co-transformation of wheat stem rust resistance gene *Sr50* and corresponding avirulence gene *AvrSr50* known to induce necrosis in wheat protoplasts was used as the control. Luciferase activity values were measured from three biological replicates (n = 3) to assess cell viability. Error bars represent s.d. of three biological replicates. Significance of differences was analyzed by one-way ANOVA followed by Tukey’s multiple comparisons test. P > 0.05 was considered not significant.

We agree that the current data were insufficient to support the original claim that these domains "are strictly upregulated during infection leading to hypersensitive responses.", and we removed this statement from the revised manuscript.

11. Lines 355–356: No evidence is provided to support the proposed mechanism (e.g., measurements of Ca^{2+} flux or ion efflux). It is also unclear how the response is initiated

and whether an effector or other specificity determinants are involved.

Response: Thank you for your comments. We measured the Ca^{2+} influx in mesophyll tissue of the transformation line T_3-17-5 (Fielder+*Yr26*) and WT accession Fielder at 1dpi and 5dpi following inoculation with CYR32 (Fig. 4i). Plants treated with electrolytic fluorinating solution served as the control. In the control treatment, the Ca^{2+} influx rate in T_3-17-5 (-48.8 and -88.4 pico mol cm^{-2} s^{-1}) was slightly higher than in WT (-38.4 and -59.9 pico mol cm^{-2} s^{-1}), but both remained at relatively low levels at 1 dpi and 5 dpi (Fig. 4i; Supplementary Table 5). Upon CYR32 inoculation, Ca^{2+} influx rate increased dramatically in both T_3-17-5 (-519.3 and -813.0 pico mol cm^{-2} s^{-1}) and WT (-425.4 and -612.5 pico mol cm^{-2} s^{-1}), indicating that *Pst* infection induces Ca^{2+} influx. Compared to the WT, T_3-17-5 exhibited approximately 22.08% and 32.73% faster Ca^{2+} influx rate at 1 dpi and 5 dpi, respectively, suggesting that *Yr26* activates Ca^{2+} influx signals upon *Pst* challenge. Furthermore, when the Ca^{2+} blocker $LaCl_3$ (2 mM) was applied during expression of Yr26 in *Nicotiana benthamiana*, the cell death typically triggered by Yr26 was abolished, confirming the Ca^{2+} plays an important role in the Yr26-mediated immune response (Fig. 4j).

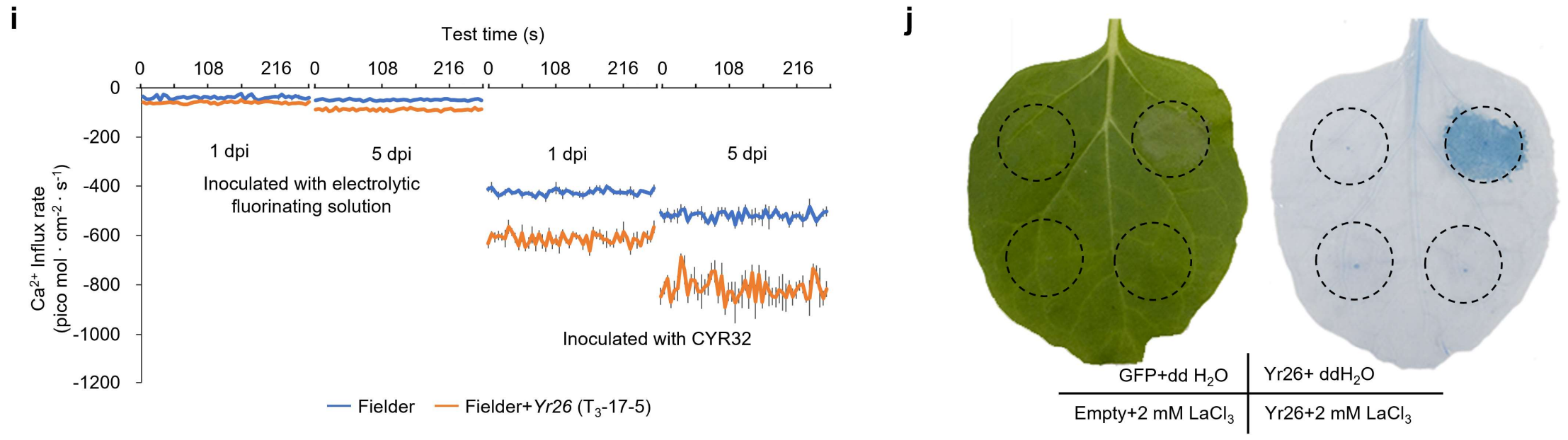

Fig. 4i-j Yr26-mediated resistance requires calcium influx, as shown by NMT analysis and $LaCl_3$ blockade

i. Ca^{2+} influx rate in transgene line T_3-17-5 (Fielder+*Yr26*) and WT line Fielder leaves infected with *Pst* race CYR32 at 1dpi and 5 dpi. Leaves inoculated with electrolytic fluorinating solution served as mock control. For each treatment, Ca^{2+} influx rate was continuously monitored for approximately 5 minutes using Non-invasive Micro-test Technology (NMT), and four independent leaves ($n = 4$) were measured.

j. Suppression of *Yr26* overexpression induced cell death by the calcium channel blocker $LaCl_3$ in *N. benthamiana* leaves. Agrobacterium cultures harboring the indicated constructs (GFP, empty vector, and *Yr26*) were infiltrated into *N. benthamiana leaves*. Eight hours after *Agrobacterium* infiltration, the infiltrated region was further treated with either water or 2 mM $LaCl_3$, a calcium

channel blocker. Cell death phenotypes were assessed at 2 days post-infiltration (dpi) using trypan blue staining (dead cells are stained blue). Expression of *Yr26* alone induced a clear cell death phenotype, whereas co-infiltration with $LaCl_3$ abolished this response. No cell death was observed in the GFP, empty vector, or empty vector plus $LaCl_3$ control regions. Similar results were obtained from three independent biological replicates (n = 3).

Taken together, these data demonstrate that *Yr26*-mediated immunity requires Ca^{2+} influx. However, we acknowledge that direct evidence for *Yr26* itself forming a pentameric cation channel as WeiTsing is currently lacking. We have therefore tightened the discussion to clearly state that the channel-like mechanism remains a hypothesis based on structural prediction (AlphaFold), transmembrane architecture, and functional Ca^{2+} dependency, rather than a demonstrated activity. These observations raise the possibility that Yr26 may function as part of an ion channel complex facilitating Ca^{2+} influx, analogous to WTS. And direct structural and electrophysiological evidence is required to test this hypothesis.

Based on the virulence assays against 166 *Pst* races and the resistance evaluation against leaf rust and powdery mildew, combined with the expression analysis of Yr26 in response to different elicitors (as shown in our response to Comment 1), we found that Yr26 can be specifically induced by *Pst* regardless of whether the race is virulent or avirulent. This observation suggests the existence of a conserved factor in *Pst* that triggers Yr26 expression. Furthermore, although Yr26 overexpression is sufficient to induce necrosis in *N. benthamiana* and wheat protoplast, no necrosis was observed upon infection with virulent *Pst* races. This discrepancy implies that virulent races may encode one or more effector proteins capable of suppressing Yr26-mediated immune responses. Therefore, we propose that Yr26 expression can be induced by both avirulent and virulent isolates, and that race specificity is determined not merely by transcriptional activation but likely by additional post-transcriptional or post-translational mechanisms, or by the presence of suppressive factors in virulent isolates.

12. Lines 416–420: This sentence requires revision.

a) Only a single isolate was tested.

b) The study does not substantially expand the mechanistic repertoire of disease-

resistance genes, as mechanistic insight is limited.

c) It is unclear how the study represents an advancement in understanding the role of newly evolved genes in wheat plant–pathogen interactions

Response: Thank you for these critical comments. We agree that the original statements on lines 416–420 were overextended and not fully supported by the data. We have therefore revised this section substantially based on our new results. Specifically, "In summary, we isolated the all-stage resistance gene *Yr26* that encodes a unique predicted transmembrane protein and showed that it was a recently evolved orphan gene within the *Triticeae* lineage and delineate a multi-step trajectory through which a non-functional ancestral sequence acquired race-specific resistance function. The gene triggers a Ca^{2+}-dependent hypersensitive response upon recognition of avirulent *Pst* isolates, and that its C-terminal Domain 3 is both necessary and sufficient for cell death induction. These findings expand the repertoire of functionally validated disease resistance genes, provide a case that orphan genes can evolve *de novo* to fulfill critical roles in plant-pathogen interactions, and offer a valuable diagnostic marker for marker-assisted breeding. Future work aimed at elucidating the molecular recognition mechanism, determining the high-resolution structure, and combining *Yr26* with other resistance genes should accelerate the development of durable stripe rust resistance in wheat".

13. Line 863: Please describe the methods itself (DAB and FITC WGA staining)

Response: Thank you for your suggestion. The detailed methods for DAB and WGA staining have been described independently in the revised manuscript.

DAB staining for H_2O_2 detection To assess the accumulation of H_2O_2 as a cellular response to *Pst* infection, a diaminobenzidine (DAB) staining assay was performed. Infected wheat leaves were excised and treated with a 1.5 mg/mL DAB solution (3,3'-diaminobenzidine; Sigma-Aldrich, USA). The samples were then incubated under light at room temperature for 6 hours to allow the peroxidase-mediated polymerization of DAB, which forms a reddish-brown precipitate at sites of H_2O_2 accumulation. More

than 30 infection sites were observed for each treatment.

WGA-Alexa 488 staining for fungal structures To visualize fungal hyphae and infection structures within *Pst*-infected wheat leaves, samples were first fixed and decolorized in a mixture of acetic acid and ethanol (1:1) for 3 days. Following decolorization, the leaf samples were stained with wheat germ agglutinin (WGA) conjugated to Alexa-488 (Invitrogen, Carlsbad, CA, USA). WGA binds specifically to chitin and other glycoconjugates present in fungal cell walls. The stained samples were observed under blue-light excitation (excitation wavelength, 450–480 nm; emission wavelength, 515 nm) using a confocal microscope (Olympus BX-53). More than 30 infection sites were observed for each treatment.

Reviewer #1 (Remarks to the Author):

This revised manuscript made significant revision based on the comments of the reviewers. The authors performed additional experiments and provided strong new information to support their findings. I was satisfied with the scientific meaning and content of the revision. I have no further comments for the revised manuscript.

Response: We are very grateful to you for the positive assessment and for confirming that the revisions and additional experiments satisfactorily addressed all previous concerns.

Reviewer #2 (Remarks to the Author):

The revised manuscript is substantially improved and now addresses several of the major concerns raised previously. The genetic evidence identifying Yr26/Yr24/YrCH42/YrG22 as the same gene is strong and convincing, supported by fine mapping, BAC-based candidate identification, EMS mutant analysis, BSMV silencing, CRISPR knockout, and transgenic complementation. The manuscript is also strengthened by the expanded haplotype analysis, the more careful evolutionary interpretation, and the newly added data supporting a Ca^{2+}-dependent component of Yr26-triggered cell death. In particular, the addition of Ca^{2+} influx measurements in the Yr26 transgenic line and the suppression of Yr26-triggered cell death by $LaCl_3$ meaningfully reinforce the proposed functional model, while the rebuttal appropriately acknowledges that direct evidence for Yr26 itself acting as a cation channel is still lacking.

Response: We sincerely thank you for the positive feedback, and we carefully noted the remaining limitation regarding direct channel evidence in the Discussion, as suggested.

It is appreciated that the authors have improved the framing of several previously overstated points. The revised manuscript now better resolves the issue of the identity of Yr24/Yr26/YrCH42/YrG22, more cautiously interprets the evolutionary origin of the functional resistant allele after additional screening of wild emmer accessions, and provides new evidence that the resistant Yr26_h1 allele should be distinguished from

non-functional or uncertain haplotypes. The added VIGS and F2-based analyses of Yr26_h5 are especially helpful in showing that the resistance phenotype in the corresponding accession is likely due to other gene(s), not Yr26_h5 itself.

Response: We are grateful for the positive feedback and are glad that the revised interpretations and the *Yr26_h5* analyses were helpful and appropriate.

A few points still need attention. First, the revised text still contains several internal inconsistencies and residual duplicated passages, especially in the sections describing Yr26 expression under avirulent versus virulent races and the interpretation of Domain 3 function. These need to be carefully reconciled so that the final narrative is fully consistent. Second, although the Ca^{2+}-related evidence is a real improvement, the manuscript should continue to avoid wording that could be interpreted as demonstrating that Yr26 itself forms a Ca^{2+} channel or pore. The current data support a Ca^{2+}-dependent mechanism and are consistent with such a possibility, but they do not yet prove it. Third, the statement that SNP4 is likely the key causal polymorphism distinguishing Yr26_h1 from Yr26_h2 is plausible but still inferential and should remain framed as such. Finally, the manuscript should be checked carefully to ensure that all figure legends fully match the improved statistical reporting described in the rebuttal.

Response: We sincerely thank you for these thoughtful and constructive suggestions. We carefully addressed each point as follows:

(1) Internal inconsistencies and duplicated passages We thoroughly reconciled the text regarding Yr26 expression under different races and Domain 3 function. Duplicated passages were removed, and the narrative is now fully consistent.

(2) Ca^{2+} We revised the language throughout to ensure no statement implies that Yr26 itself forms a channel or pore. The mechanism is now described as "Ca^{2+}-dependent" without overinterpretation.

(3) SNP4 causality The description of SNP4 was reframed as a plausible candidate rather than a proven causal polymorphism, with appropriate cautionary language added.

(4) Figure legends All figure legends were double-checked and updated to match the improved statistical reporting.

Reviewer #3 (Remarks to the Author):

The authors have done impressive additional work that fully addresses all comments of this reviewer. They performed a substantial amount of new experimental work, including correcting the expression profiles, adding rigorous protein controls, significantly expanding the evolutionary analysis, and successfully tracking real-time cellular calcium dynamics and protein localization. The manuscript text and figures were revised accordingly and are now much stronger. I have no further comments.

Response: Thank you very much for the thoughtful review. We are pleased that all previous concerns were fully addressed.

Reviewer #2 (Remarks to the Author):

The revised manuscript is substantially improved and now addresses the major concerns raised previously. The evidence identifying Yr26/Yr24/YrCH42/YrG22 as the same gene remains strong and convincing, supported by fine mapping, BAC-based candidate identification, EMS mutant analysis, BSMV silencing, CRISPR knockout, and transgenic complementation. The manuscript is also strengthened by the expanded haplotype and evolutionary analyses, the clarified race-specificity framework, and the added Ca^{2+}-related experiments. In particular, the revised Results and Discussion now present a coherent model in which Yr26 is specifically induced by Pst, confers race-specific resistance, and triggers a Ca^{2+}-dependent cell-death response, while appropriately acknowledging that direct evidence that Yr26 itself functions as a channel is still lacking.

The manuscript also appears to have resolved the previous internal inconsistencies, especially regarding Yr26 expression under avirulent versus virulent races and the interpretation of Domain 3 function. The evolutionary interpretation is more careful than before, and the statement about SNP4 is now framed as a likely rather than proven causal change. These are important improvements.

Response: We sincerely appreciate the time and expertise you have devoted to reviewing our manuscript. We are encouraged by your positive recognition of our revisions and have carefully followed your suggestions to further refine the interpretation of our findings.

Only a small number of issues remain. First, the Abstract still states that the C-terminal four-helical bundle domain is critical for triggering cell death "by mediating Ca^{2+} influx," which is somewhat stronger than the more appropriately cautious wording used later in the manuscript. Based on the current evidence, a Ca^{2+}-dependent mechanism is supported, but direct mediation of Ca^{2+} influx by Yr26 itself is not yet demonstrated. Second, the front matter would benefit from one final wording adjustment so that the statements about the emergence of Yr26 are fully aligned with the more careful

evolutionary interpretation presented later for the functional resistant allele.

Response: We greatly appreciate the reviewer's rigorous and meticulous reading of our manuscript. In response to the two remaining wording issues, We have revised the Abstract to state that the C-terminal four-helical bundle domain triggers a Ca^{2+}-dependent cell-death response, without claiming direct mediation of Ca^{2+} influx by Yr26 itself. In the Introduction, we revised the final sentence to refer specifically to the 'Yr26 functional resistant allele' and to state that it 'likely first emerged in emmer wheat,' thereby fully aligning the front matter with the more cautious evolutionary interpretation presented in the Results.
