# Supplementary material for: A transmembrane protein confers the Yr26/Yr24/YrCH42/YrG22-mediated stripe rust resistance in wheat
Source: Nat Commun. 2026 Aug 24;17:10389. doi: 10.1038/s41467-026-76091-5 (PMC13627085; doi:10.1038/s41467-026-76091-5)
Supplement: Supplementary file 4 [file 41467_2026_76091_MOESM3_ESM.pdf]

Description of Additional Supplementary Files

File Name: Supplementary Data 1

Description: Virulence formulae of *Puccinia striiformis* f. sp. *tritici* (*Pst*) races used in this study.

File Name: Supplementary Data 2

Description: Molecular markers used in the fine mapping of *YR26*.

File Name: Supplementary Data 3

Description: Annotated genes in the *YR26* target interval in the Chinese Spring reference genome and 92R137 contig02.

File Name: Supplementary Data 4

Description: List of 166 *Puccinia striiformis* f. sp. *tritici* (*Pst*) isolates collected from fielder in China during 2023-2025

File Name: Supplementary Data 5

Description: Ca^{2+} influx rate in transgene line T_3-17-5 (Fielder+*Yr26*) and wild-type cultivar Fielder.

File Name: Supplementary Data 6

Description: Tetraploid and synthetic-derived wheat genotypes used in discovery of *YR26* haplotypes.

File Name: Supplementary Data 7

Description: Genotypes at the *DM2* marker and phenotypes to *Puccinia striiformis* f. sp. *tritici* (*Pst*) race CYR32 in the DEW075/D026 F_2 population.

File Name: Supplementary Data 8

Description: Modern Chinese wheat genotypes in this study.

File Name: Supplementary Data 9

Description: DNA and protein sequence identity of *YR26* and its homologs in assembled genomes with *Yr26_h1*.

File Name: Supplementary Data 10

Description: Genotypes of wheat accessions used in this study based on public re-sequencing data.

File Name: Supplementary Data 11

Description: Statistics of *YR26* lineages in tetraploid and hexaploid wheat.

File Name: Supplementary Data 12

Description: Sequences of primers used in this study.
